# Nano- and microparticles at fluid and biological interfaces

**DOI:** 10.1088/1361-648X/aa7933

**Published:** 2017-08-11

**Authors:** S Dasgupta, T Auth, G Gompper

**Affiliations:** 1 Mechanobiology Institute, National University of Singapore, Singapore 117411, Singaporesabyadg.softbio@gmail.com; 2 Institut Curie, CNRS, UMR 168, 75005 Paris, France; 3 Theoretical Soft Matter and Biophysics, Institute of Complex Systems and Institute for Advanced Simulation, Forschungszentrum Jülich, 52425 Jülich, Germanyt.auth@fz-juelich.deg.gompper@fz-juelich.de; 4 Present address: Department of Physics, University of Toronto, Toronto, Ontario M5S1A7, Canada

**Keywords:** membranes, nanoparticles, capillary interactions, lipid bilayers, emulsions, viruses, interfaces

## Abstract

Systems with interfaces are abundant in both technological applications and biology. While a fluid interface separates two fluids, membranes separate the inside of vesicles from the outside, the interior of biological cells from the environment, and compartmentalize cells into organelles. The physical properties of interfaces are characterized by interface tension, those of membranes are characterized by bending and stretching elasticity. Amphiphilic molecules like surfactants that are added to a system with two immiscible fluids decrease the interface tension and induce a bending rigidity. Lipid bilayer membranes of vesicles can be stretched or compressed by osmotic pressure; in biological cells, also the presence of a cytoskeleton can induce membrane tension. If the thickness of the interface or the membrane is small compared with its lateral extension, both can be described using two-dimensional mathematical surfaces embedded in three-dimensional space. We review recent work on the interaction of particles with interfaces and membranes. This can be micrometer-sized particles at interfaces that stabilise emulsions or form colloidosomes, as well as typically nanometer-sized particles at membranes, such as viruses, parasites, and engineered drug delivery systems. In both cases, we first discuss the interaction of single particles with interfaces and membranes, e.g. particles in external fields, non-spherical particles, and particles at curved interfaces, followed by interface-mediated interaction between two particles, many-particle interactions, interface and membrane curvature-induced phenomena, and applications.

## 1. Introduction

Interfaces are present in all systems with two or more phases. This can be interfaces between fluid and gas phases, but also interfaces between immiscible fluids, such as oil and water, and interfaces between fluid and solid phases. Popular applications for particles at interfaces are Pickering emulsions and bijels [1–5]. Already in the early 20th century, particles have been found to assemble at interfaces and to stabilize emulsions [1, 3]. The stabilization depends on particle size, shape, softness, and surface heterogeneities, e.g. generated by functionalization.

Membranes are ubiquitous in biological cells. While the plasma membrane encloses the entire cell, membranes also compartmentalize cells and thereby define organelles. Transmembrane transport is essential for the communication both inside a cell as well as of cells with their environment [6, 7]. The interaction of particles and pathogens with biological membranes—and therefore also their cellular uptake and intracellular transport—crucially depends on the particle size, shape, softness, and surface functionalization.

**Figure 1.**
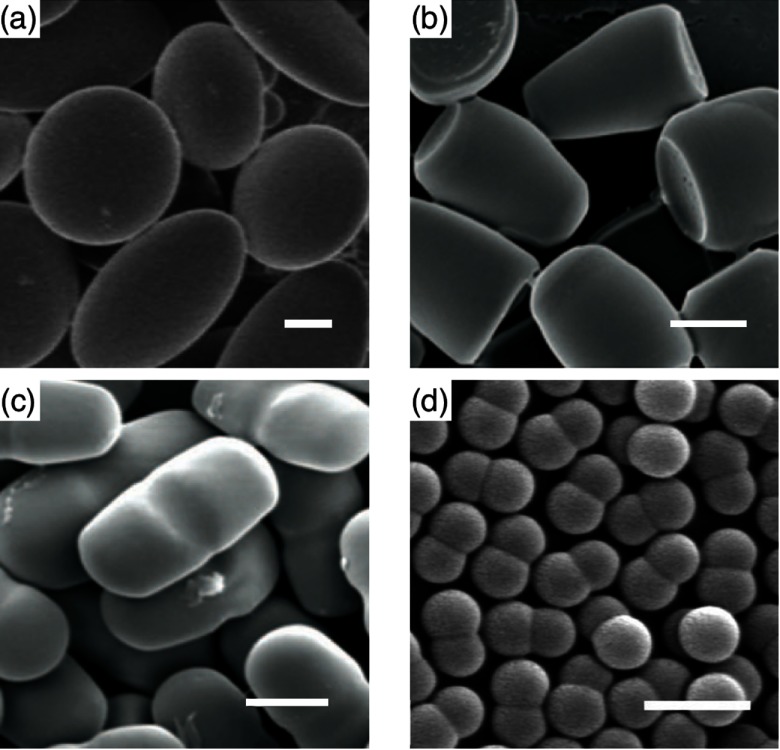

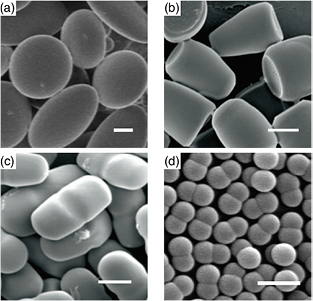

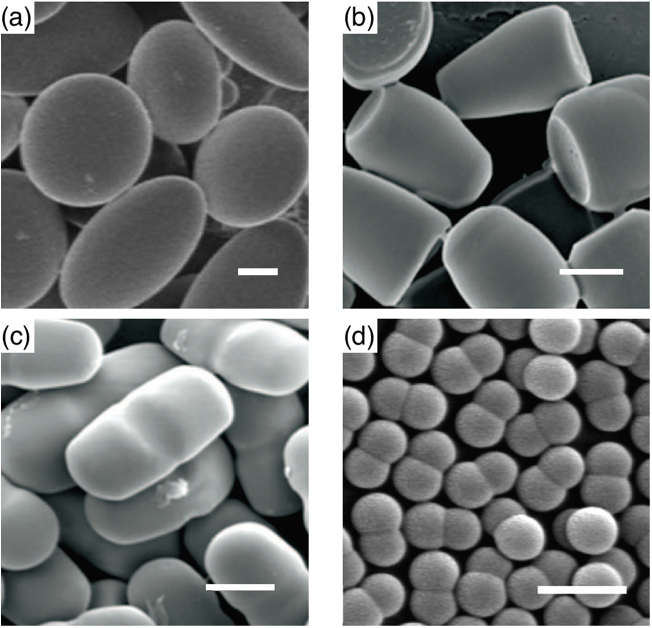
Examples for microparticles: (a) oblate, disk-shaped, (b) bullet-shaped, and (c) pill-shaped polymeric particles. The length of the scale bars corresponds to . Adapted with permission from [25]. Copyright © 2007 National Academy of Sciences. (d) Dumbbell-shaped polymeric particles. The length of the scale bar corresponds to . Adapted with permission from [26]. Copyright © 2010 American Chemical Society.

**Figure 2.**
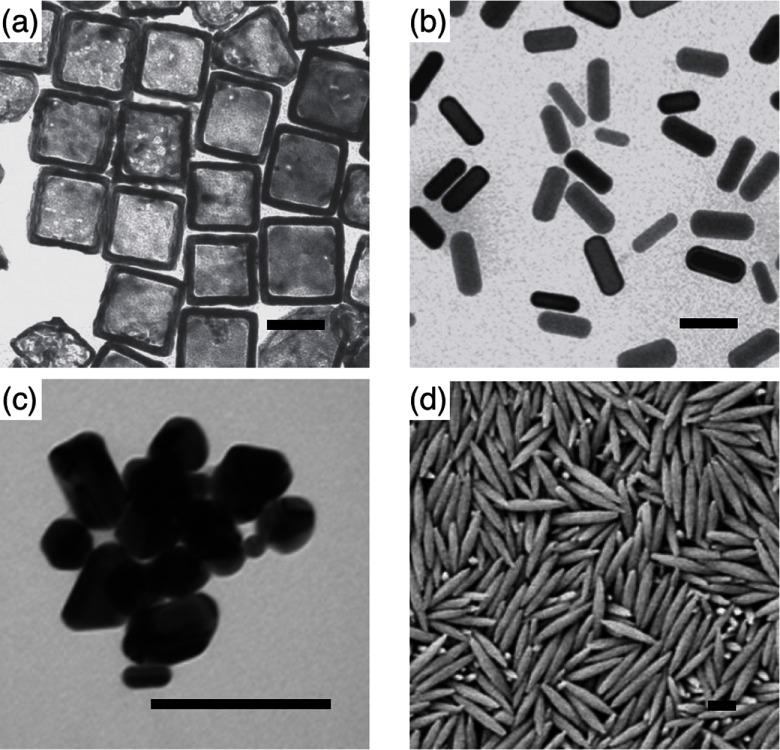

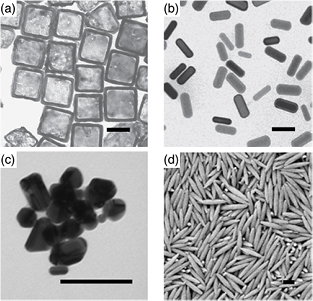

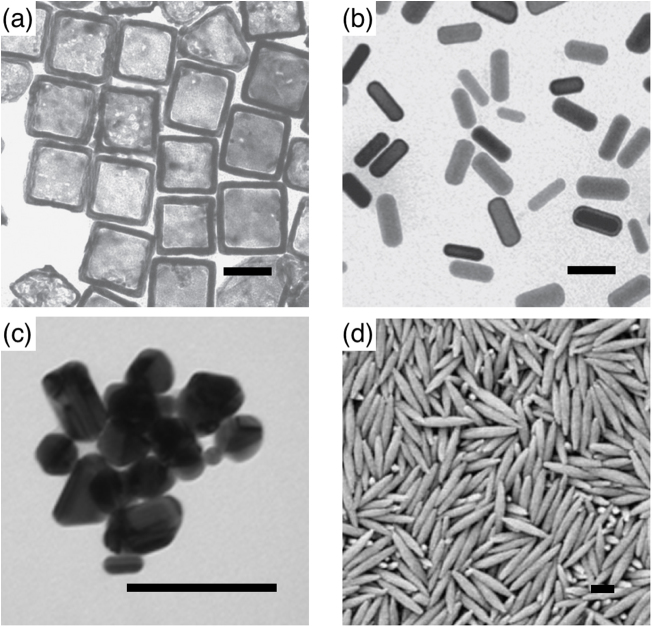
Examples for nanoparticles: (a) Cube-like and (b) rod-like gold nanoparticles. The length of the scale bars corresponds to . Adapted with permission from Macmillan Publishers Ltd: *Nat. Nanotechnol*. ([27]), copyright © 2011. (c) Silver nanoparticles with irregular shapes. Adapted with permission from [19]. OA CC BY 4.0. (d) Hematite nanospindles. The length of the scale bar corresponds to . Reprinted with permission from [30]—Published by The Royal Society of Chemistry. CC BY 3.0.

Nowadays a whole zoo of micro- and nanoparticles can be fabricated from various materials, with engineered shapes and surface functionalizations. The particles can be used for applications in food science [8–10], cosmetics [8, 9, 11], as antimicrobials [12, 13], and in nanomedicine [14–16]; therefore systematic studies and a careful consideration of potentially toxic effects are required [17–21]. Figure 1 shows examples for oblate, bullet-shaped, pill-shaped, and dumbbell-shaped microparticles that are made from a polymeric material. Figure 2 shows cube-like, rod-like, irregularly-shaped, and spindle-like metal and metal-oxide nanoparticles. All these particles can also be considered as model systems for ‘particles’ found in nature. For example, the malaria parasite is micrometer-sized and has an egg-like shape [22, 23]. Milk contains casein micelles with sizes below  that stabilize fat globules [24]. Viruses resemble roundish, filament-like, and bullet shapes with sizes below  [25–27]; in particular, the filamentous Ebola and Marburg viruses are of much interest due to their enhanced virulence that leads to high mortality rates [28, 29].

**Figure 3.**
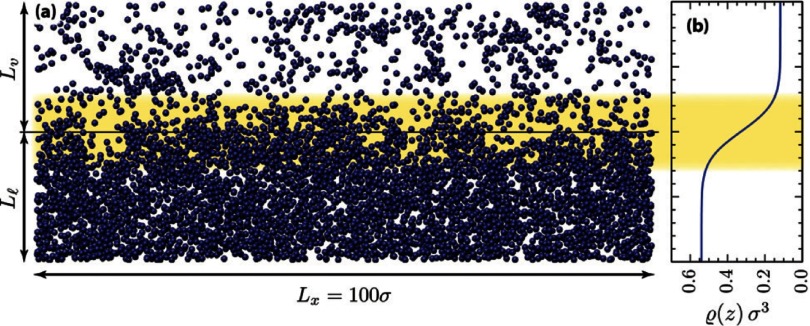

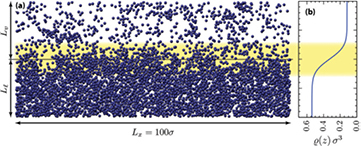

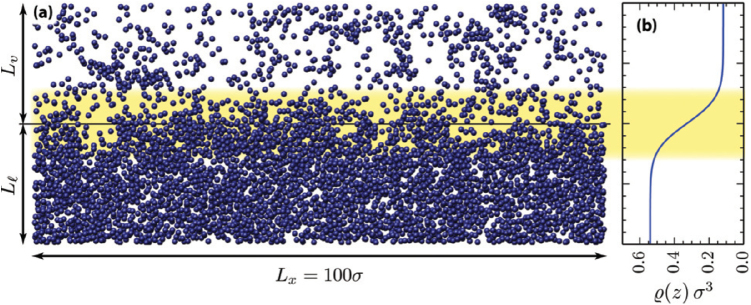
Simulation snapshot of the liquid-vapour interface for a Lennard-Jones fluid with pair potential  at . (a) A vertical slice of width  and height  is shown with the interface region highlighted. (b) Corresponding mean density profile . Reproduced with permission from [31]. © EPLA. All rights reserved.

Fluid interfaces are rough on the molecular scale and can be analytically well described by a hyperbolic tangent-shaped density profile [31, 33], see figure 3. However, the typical interface width is much smaller than the sizes of the particles that we consider. In order to study the interaction of particles with interfaces, the interface can therefore be thought of as mathematical surface with its physical properties characterized by an interface tension.

**Figure 4.**
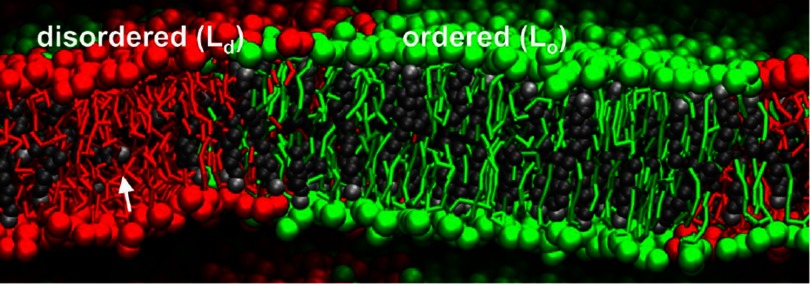

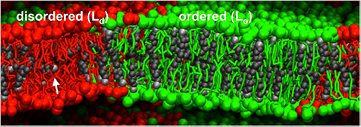

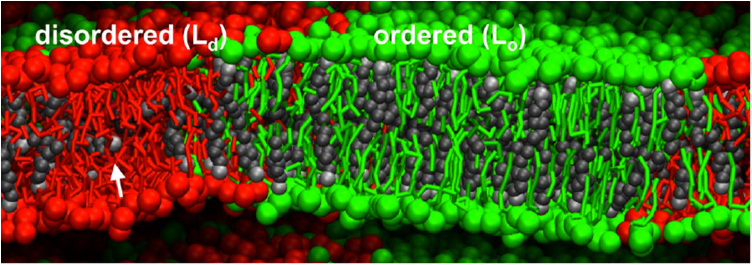
Structural and dynamic properties of the membrane domains. Side view of the planar di-PC/di-PC/cholesterol 0.42:0.28:0.3 system, revealing the molecular organization in both the  and  phases. The white arrow points to a cholesterol oriented in between the monolayer leaflets. Reprinted with permission from [32]. Copyright © 2008 National Academy of Sciences.

Lipid-bilayer membranes often consist of many components, e.g. different lipids and cholesterol, and biological membranes usually also contain membrane proteins. This can lead to phase separation within the membrane, as shown in figure 4. The typical thickness of a lipid bilayer membrane is  and cannot be neglected for small nanoparticles with sizes of few nanometers. For nanoparticles with radii of  and above, the membrane can be described as mathematical surface with curvature-elastic properties.

The possiblity to model both fluid interfaces and biological interfaces (membranes) using mathematical surfaces is our motivation for discussing particles at interfaces and at membranes in a single review article. For biological interfaces, we will focus on sufficiently large particles for which a continuum model is not only feasible, but also more appropriate than an atomistic model. The deformation energy of the surface can then be calculated using the Helfrich Hamiltonian [34],

1

Here, the interface conformation is characterized by the two principal curvatures at each point of the interface, *c*_1_ and *c*_2_, that enter the Hamiltonian via the mean curvature  and the Gaussian curvature . The total deformation energy is obtained by integration over the entire interface area *S*. Tension *γ*, bending rigidity *κ*, spontaneous curvature *c*_0_, and Gaussian saddle splay modulus  describe the mechanical and elastic properties of the interface. Equation (1) applies to biological interfaces if the bending energy contribution dominates, and to fluid interfaces if the energy is given only by the tension term.

For spherical particles at planar fluid interfaces, the particle size, the interface tensions between the particles and the two phases, as well as the interface tension between both phases characterize the system. If the interface tensions between the particles and both phases are identical, the particles attach to the interface because their presence reduces direct contact between both phases without any additional costs for the contact of the particles with the fluids. For high-tension interfaces and micrometer-sized particles, such as for silica particles at oil–water interfaces, the attachment energy gain can be as high as  [41]. Therefore, such particles are irreversibly adsorbed to the interface. If the interface tensions of the particles with both fluids differ, the attachment energies of the particles can be strongly reduced.

For particles attached to membranes, the membrane elastic properties in equation (1) and the adhesion energy between particles and membranes

2 characterize the system. Here, *S*_ad_ is the membrane area adhered to the particle, and the adhesion strength *w* for the contact interaction can be mediated by van der Waals forces, by electrostatic interactions, and by specific adhesion (receptor-ligand bonds). For small spherical particles attached to nearly planar membranes and weak adhesion strengths, wrapping may not be energetically favourable, while for spherical particles with a radius of  and for a high adhesion strength  the energy gain through wrapping can be as high as . Particle shape can strongly alter the attachment energy.

Table 1 provides an overview of typical attachment energies of spherical particles to interfaces and membranes. For particles at interfaces, we assume that they have equal interface tensions with both fluid phases and that they are therefore half immersed in each phase. For particles at membranes, we assume that they are attached to a membrane with half of their surface area and that there is no deformation energy cost for the membrane surrounding the particles^5^5Our estimates are based on the overview of adhesion strengths provided in [42]..

**Table 1.** Estimated attachment energies *E*_A_ (i) for spherical particles of radius *r*_p_ attached to an oil–water interface with a typical interface tension  [35], assuming that the interface tensions of the particle with both phases are identical and (ii) for spherical particles that are half-wrapped by a membrane with bending rigidity  [36, 37]. We use the particle-membrane adhesion strength  between DMPC and silica ( is reported in [38]), the adhesion strength  for receptor-ligand bond-mediated interaction ( is reported in [39]), and the adhesion strength  between DOPC/DOPG and glass ( is reported in [40]). The particle-membrane interaction is only attractive beyond a threshold particle radius.

System		
Oil–water interface		
Oil–water interface		
DMPC-silica		
DMPC-silica		
DMPC-silica		
Receptor-ligand		
Receptor-ligand		
DOPC/DOPG-glass		
DOPC/DOPG-glass		

Interface deformations induced by particles lead to interface-mediated interactions and self-assembly; many-particle systems minimize the deformation energies also with respect to the particle positions. For example, capillary forces between micrometer-sized ellipsoidal particles increase with increasing aspect ratio and the energy gain at particle contact can be as high as 10^4^– already for ellipsoid aspect ratios of 2–3 [43, 45]. Membrane-mediated binding energies of few  have been calculated for spherical particles at membranes [44, 46, 47]^6^6Contrary to particles, for spherical-cap inclusions at membranes only metastable bound states have been reported [48].. Table 2 provides an overview of interface-mediated and membrane-mediated bond energies for particles at contact.

**Table 2.** Estimated interface-mediated and membrane-mediated bond energies *E*_b_ for particles at contact for (i) micrometer-sized spherical particles and ellipsoidal particles with aspect ratio  and  at planar oil–water interfaces with interface tension  [35] and for (ii) spherical particles with radius  attached to a vesicle with membrane bending rigidity  [36, 37]. Based on [43] and [44].

System	
Spherical particles	—
Ellipsoidal particles with	
Ellipsoidal particles with	
Cuboidal particles with	
Spherical particles at membranes	

Structures on the micrometer scale can readily be observed using light microscopy. In order to access the nanometer scale, more sophisticated techniques, such as electron microscopy or super-resolution microscopy, have to be employed [49–54]. Whereas Ramsden and Pickering have reported on particle-stabilised emulsions already in the early 20th century [1, 3], images of nanoparticles, such as bacteriophages, have only been reported in 1940s, soon after electron microscopy has become available [55]. Since the very early studies of particles at fluid and biological interfaces, observation techniques and abilities to engineer particles have continuously advanced. Besides these experimental developments, also mesoscopic and atomistic modeling and computer simulation techniques and speed have rapidly developed. Both allows the characterization of particles at interfaces with increasing accuracy; this review article provides an overview of recent achievements. For both fluid and biological interfaces, we will discuss similar aspects: attachment to the interface, mechanisms for deforming the interface, particle orientation at the interface for nonspherical particles, long-range and short-range interactions, many-particle interactions, and applications. Many concepts can easily be transferred between systems with fluid and with biological interfaces. We stress this parallelity by the analogous structure of the sections for both systems. Section 2 is devoted to particles at fluid interfaces, section 3 to particles at biological membranes. The overview of the experimental studies of self-assembly of microparticles at fluid interfaces may not only benefit scientists investigating such systems, but also those interested in nanoparticles at biological membranes, where systems are less accessible experimentally and fewer studies are therefore available.

## 2. Particles at fluid interfaces

**Figure 5.**
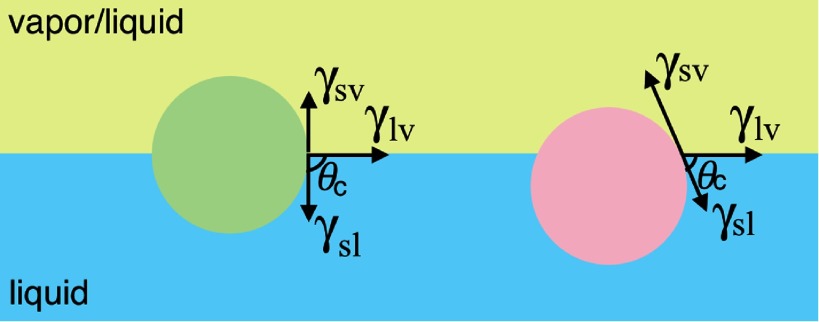

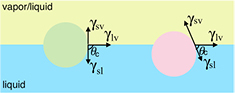

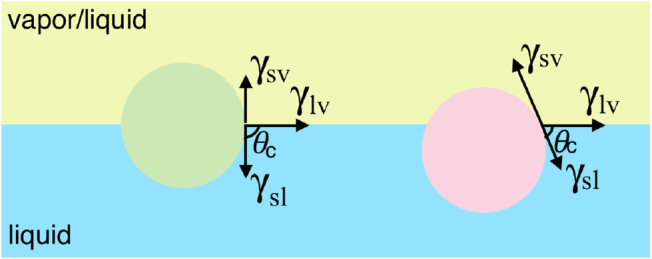
Spherical particles at a fluid-fluid or fluid-gas interface in a system with a solid spherical particle (s), a fluid (l), and a gas/second fluid (g). The contact angle  for the green particle is , while the contact angle for the red particle is smaller than . The three interface tensions , ,  are sketched for each particle using arrows. They indicate the force balance given by the Young–Dupré equation that holds for all points along the three-phase contact lines.

Particles adsorb at fluid interfaces because they reduce the interface area and thereby also the total interface energy. Here, we have to account for the interface energies between the three phases, liquid , vapor (or a second liquid) *v*, and solid *s*. The system can be characterized by the three interface tensions , , and , see figure 5. The force balance at the contact line, where the liquid-vapor interface is located at the particle, is given by the Young–Dupré equation,

3 which defines the contact angle , see figure 5. The energy difference to bring a spherical particle with radius *a* from the bulk phase to the interface is the trapping energy [56]

**Figure 6.**
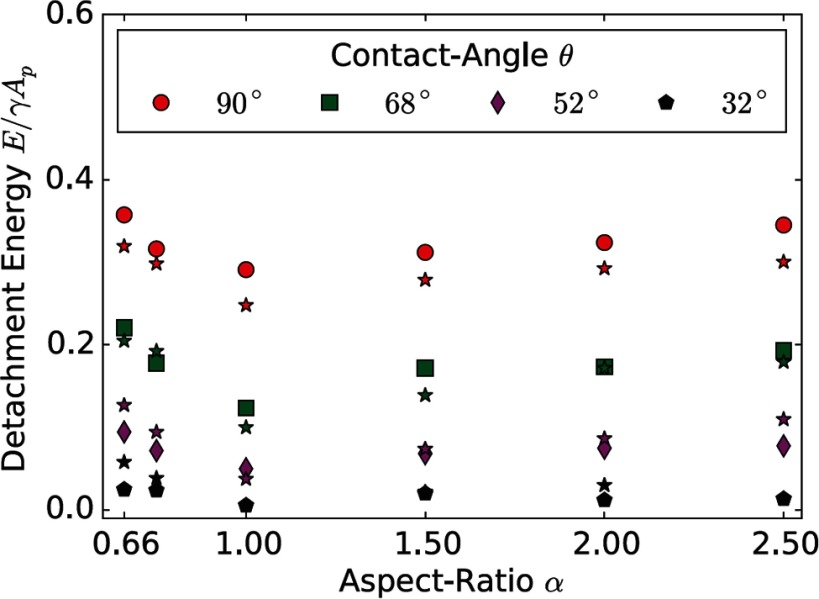

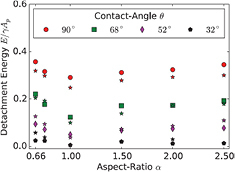

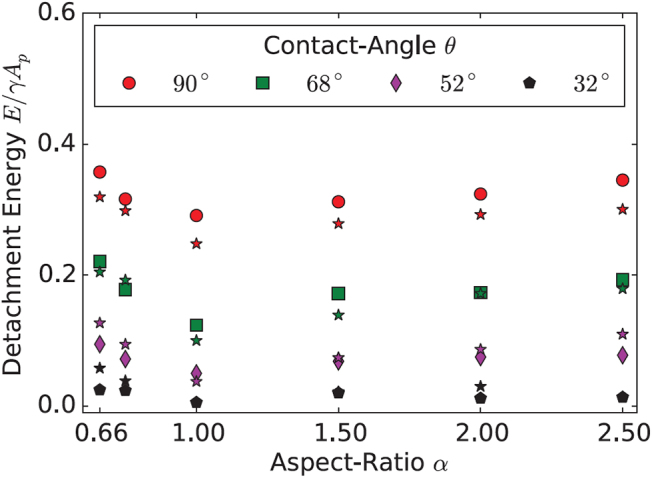
Trapping energies for spheroidal particles at flat interfaces. Numerical data obtained using Lattice Boltzmann simulations is compared with thermodynamical calculations (). Reprinted from [56] with the permission. Copyright (2014), AIP Publishing LLC.

4 where *τ* is an effective line tension and *l*_c_ is the length of the contact line. For micrometer-sized particles, the contribution of the line tension is negligible; the trapping energy is proportional to the liquid-vapor interface tension  and decreases with decreasing contact angle, see figure 6.

For small particles with sizes below one micrometer line tension becomes relevant [57], for large particles with sizes of several micrometers gravity has to be taken into account [58–60]. The Eötvös or Bond number compares the contributions of interface tension and gravity to the total energy,

5 where  is the density difference of the two phases, *g* is the gravitational acceleration, and *L* is a characteristic length of the particle. Similarly, the energies due to line tension and surface tension are compared using the dimensionless number

6

For micrometer-sized particles and typical density differences between, for example, silica and water,  [61]; for line tensions and the corresponding surface tensions measured for polystyrene and poly(methyl methacrylate) particles at oil–water interfaces, – [57]. In the following sections, we mainly discuss systems with  and , where both gravity as well as line tension can be neglected.

The estimate for  indicates that line tension may not always be entirely negligible for small micrometer-sized and large nanometer-sized particles. Line tension may qualitatively alter the behaviour of non-spherical particles at interfaces: for example, ellipsoidal and cylinderical particles may undergo orientational changes in order to minimise the length of the contact line. In [57], contact angles have been measured for spherical particles and for prolate ellipsoidal particles with various aspect ratios obtained by deforming initially spherical particles. Here, an apparent decrease of the contact angle with increasing aspect ratio of the particles has been measured. This can be attributed to a line tension and a contact angle that is independent of particle shape [57]. The experiment predicts an effective line tension  that also includes experimentally-observed heterogeneities in the contact line. Other values for line tensions reported in the literature are in the  to  range [62].

One of the key reasons for the stability of colloidal assemblies at interfaces and of Pickering emulsions are the very high trapping energies for the particles [56, 63], see table 1. Calculations for non-spherical particles show that the trapping energies depend only weakly on particle shape and increase for both oblate and prolate deformations of spherical particles, see figure 6. At curved interfaces, the trapping energies of spherical particles have been shown to depend on the interface curvature and therefore on the Laplace pressure difference at the interface [64]. For spherical particles and a contact angle , the trapping energy decreases with increasing interface curvature. For contact angles , the trapping energy either increases or decreases depending on the sign of the contact angle and on whether the interface is curved towards or away from the particles.

To satisfy the Young–Dupré equation locally at every point on the three-phase contact line, see figure 5, the contact lines and therefore also the interfaces are often not planar for non-spherical particles. Also particle-surface inhomogeneities, either due to roughness or chemical surface patterning, e.g. for Janus particles, may induce contact-line undulations. Furthermore, imposed fields, such as gravity for large -sized particles or electromagnetic fields for electrically-charged or for magnetic particles, may lead to interface deformations. The capillary forces due to the system’s tendency to reduce the total interface area between liquid and vapor is the origin of the capillary forces between particles, see table 2; very strong bond energies of  for micrometer-sized particles correspond to interface height perturbations of only  [43].

The importance of hydrodynamic interactions for the dynamics of particles at interfaces is characterized by the capillary number

7 with fluid viscosity *η* and typical particle velocity *v*. For typical velocities of  for particles with sizes of up to few micrometers in water [65], we find . Therefore, for the systems discussed in this review article , such that hydrodynamic interactions can be neglected. Particle velocities can therefore be directly related to the forces acting on the particles and the friction by the fluid.

Self-assembly of colloidal particles at fluid interfaces is governed by both direct interactions, such as van der Waals and electrostatic interactions, and indirect interactions, such as forces due to overlap of interface distortions, popularly coined as capillary interactions [62, 66]. In 1980, Pieranski reported a two-dimensional colloidal crystal of spherical polystyrene colloids with a triangular lattice structure at an air-water interface induced by an asymmetric charge distribution [63], forced into two dimensions by the interface. A large number of more recent studies on colloids at interfaces that assemble due to interface-mediated and direct interactions show a rich variety of two-dimensional structures.

An overview of both single-particle and many-particle systems at interfaces is provided in the remainder of this section. In sections 2.1–2.4, we focus on various aspects of the interaction of single particles with interfaces, while in sections 2.5 and 2.6, we discuss two-particle and many-particle interactions, respectively. Section 2.7 focuses on the special case of particles at interfaces that are half immersed into an ordered fluid. We finally discuss applications in section 2.8.

### 2.1. Contact line deformation-induced interface deformation

An undulating contact line at a particle distorts a surrounding planar fluid interface. For a cylindrically-symmetric system, such as a spherical particle at an interface, the Young–Laplace equation that describes the interface deformation is best expressed in cylindrical coordinates [67],

8

The height profile  of the interface can then be expressed as product of a function of the radial coordinate *r* and a function of the angular coordinate *ϕ* around the sphere,

9

The general solutions,

10 and

11 are characterized by the contact line at the particle, described by , , and . The interface deformation decays faster with increasing distance from the particle the larger *m*, i.e. the larger the number of undulations along a circle around the particle.

The contact-line undulations can be expressed as multipole expansion,

12 where the contact radius *r*_c_ is often similar to the particle radius, *H*_*m*_ are the expansion coefficients, and  the phase angles. The first two terms of the expansion vanish, because without additional external forces the particles are at their optimal height and orientation at the interface. The lowest multipole contribution and the most important contribution for long-ranged interactions is the quadrupolar deformation with  [67],

13 where *H*_2_ characterizes the undulation amplitude of the contact line. The long-ranged interactions between two particles in the far field therefore decay with , where *d* is the interparticle distance.

Experimentally, imprinted contact-line shapes have to be distinguished from mere surface roughness. Whereas surface roughness often leads to temporary and random pinning of the contact line, engineered contact-line deformations are stable and well controlled. For instance, undulating plates allow the engineering of the interface deformation and the tuning of the capillary interaction between particles from attractive to repulsive [68, 69]. However, also nanometric roughness on microspheres or disc-like particles leads to strong capillary interactions due to contact-line pinning [67, 70–72]. The temporary nature of contact-line pinning can be observed for instance for metastable orientations of dumbbell-shaped particles at interfaces; the distribution of particle orientations relaxes towards the globally stable particle orientation when the system ages [73].

### 2.2. Interface deformation by non-spherical particles

**Figure 7.**
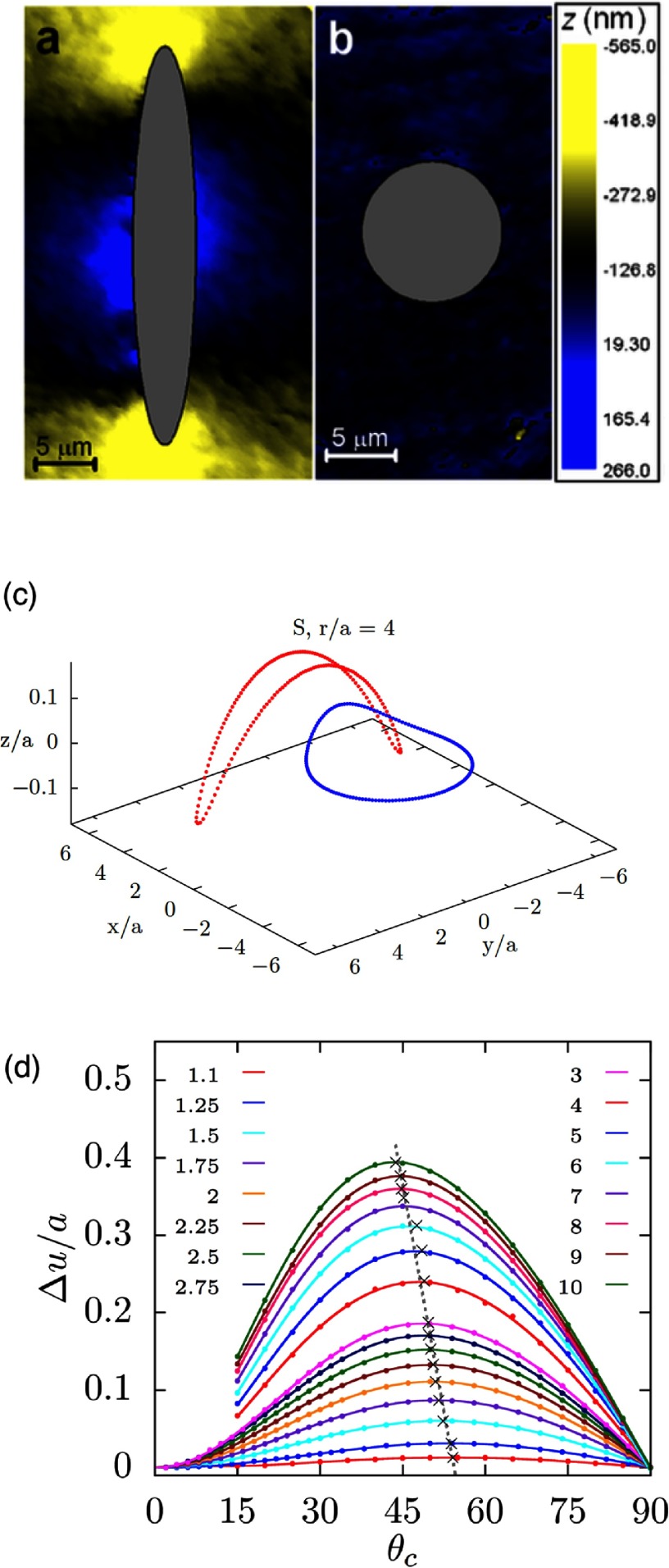

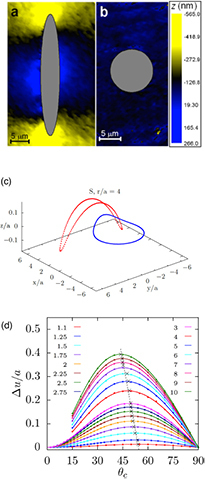

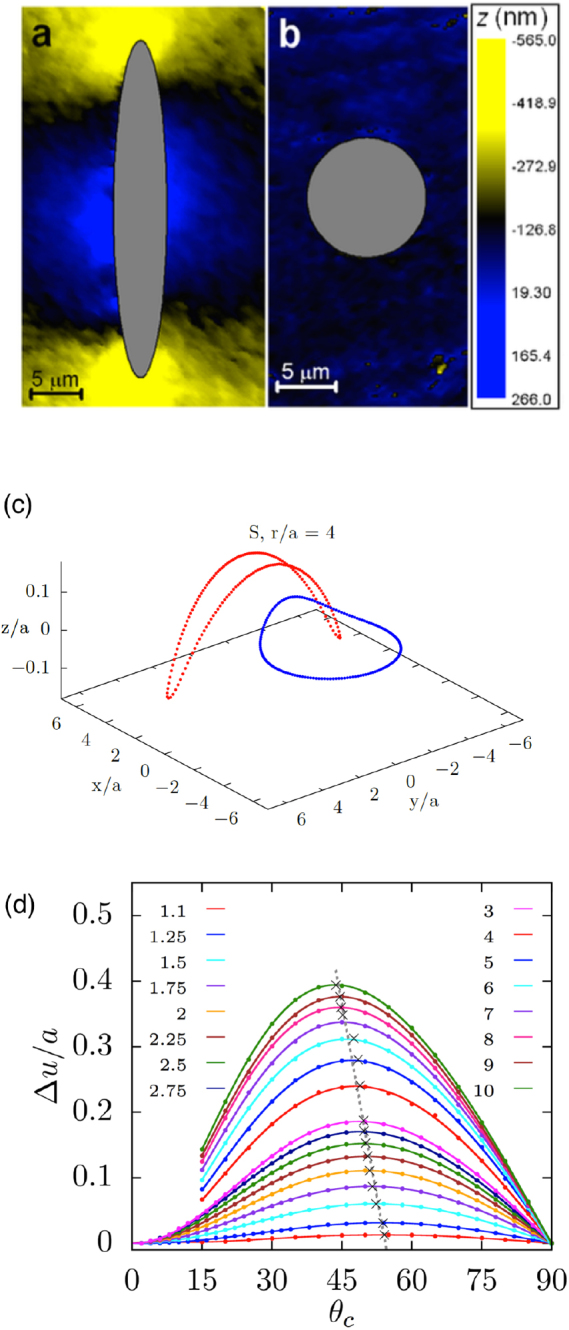
Characterization of interface deformations around particles. (a) Experimental plots of the interface distortions  around floated particles obtained from phase shifting interferometry sequences. (a) Ellipsoid. (b) Sphere. The particle bodies were artificially colored in gray. Reprinted with permission from [74]. Copyright © 2006 by the American Physical Society. (c) Contact line deformations for a spherical particle of radius  next to an ellipsoidal particle of aspect ratio  for contact angle  for both particles. The spherical particle approaches the ellipsoidal particle at the side. Reprinted with permission from [43]. Copyright (2014) American Chemical Society. (d) Maximal height difference  of the contact line for ellipsoidal particles with several aspect ratios . The numerical data are normalized by the half the length of the minor axis *a* and plotted as function of the contact angle . The maximum value of , depicted by  ×, shifts to smaller contact angles with increasing particle aspect ratio, as indicated by the grey lines that serve as guides to the eye. Reprinted with permission from [43]. Copyright (2014) American Chemical Society.

In 1992, Lucassen first suggested that particles with complex shapes can induce interface distortions in absence of gravity [69]. He systematically calculated the interaction between sinusoidal interface deformations. However, the most common interface distortion due to non-spherical particles is the quadrupolar deformation, which is found for all elongated particles with homogeneous surface functionalization at planar interfaces in the far field [65, 74, 75]. For ellipsoidal particles, quadrupolar interface deformations can also be found in the near field [43, 75, 76]. For contact angles , the interface is pulled down at the tips and pulled up near the long sides of the particle—and reverse for the inverse-wetting condition with , see figure 7(a). For ellipsoidal particles and —and for spherical particles—a planar interface remains undeformed.

Strength and nature of the interface distortions can be characterized using different quantifications: contour maps of the interface distortion, see figures 7(a) and (b), shapes of contact lines, see figure 7(c), differential heights  of the interface between the crest and trough along the contact lines, see figure 7(d), or height profiles along cross-sections, see figure 9(e). For well-defined particle shapes, in particular for ellipsoidal particles and quadrupolar interface deformations, the characterization of the interface deformation fields is uniquely determined by , where *z*_max_ is the maximum and *z*_min_ the minimum height along the contact line.

The height difference  for ellipsoidal particles vanishes for  and for  and assumes a maximal value  for  for ellipsoids with aspect ratios  [43], see figure 7(d). The position of the peak shifts from larger to smaller contact angles with increasing aspect ratios of the particles [77], while the height of the peak increases from  for  to  for . Studies are available for ellipsoidal particles [43, 74, 75], for cylindrical [65], for cuboidal particles [43], and for rounded box-like particles [70]. The height difference along the contact line of elongated particles can be thought of as a measure for their capillary interaction strength. Systematic theoretical and experimental calculations thereby provide routes to tailor capillary assembly of multiple particles.

**Figure 8.**
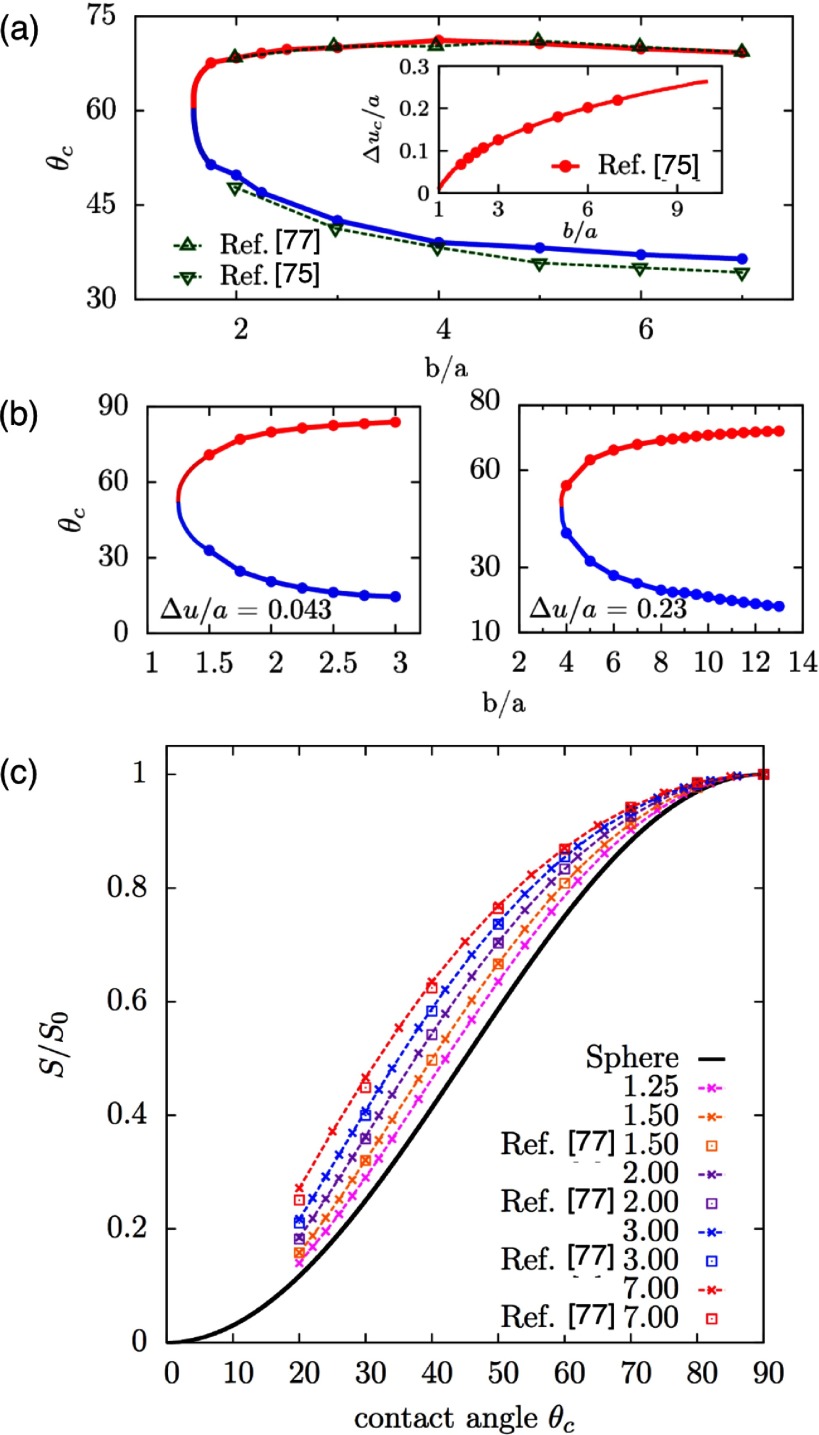

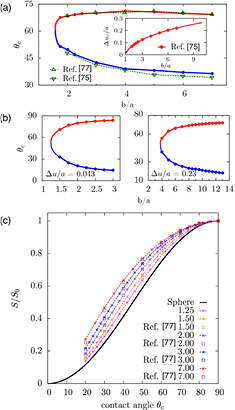

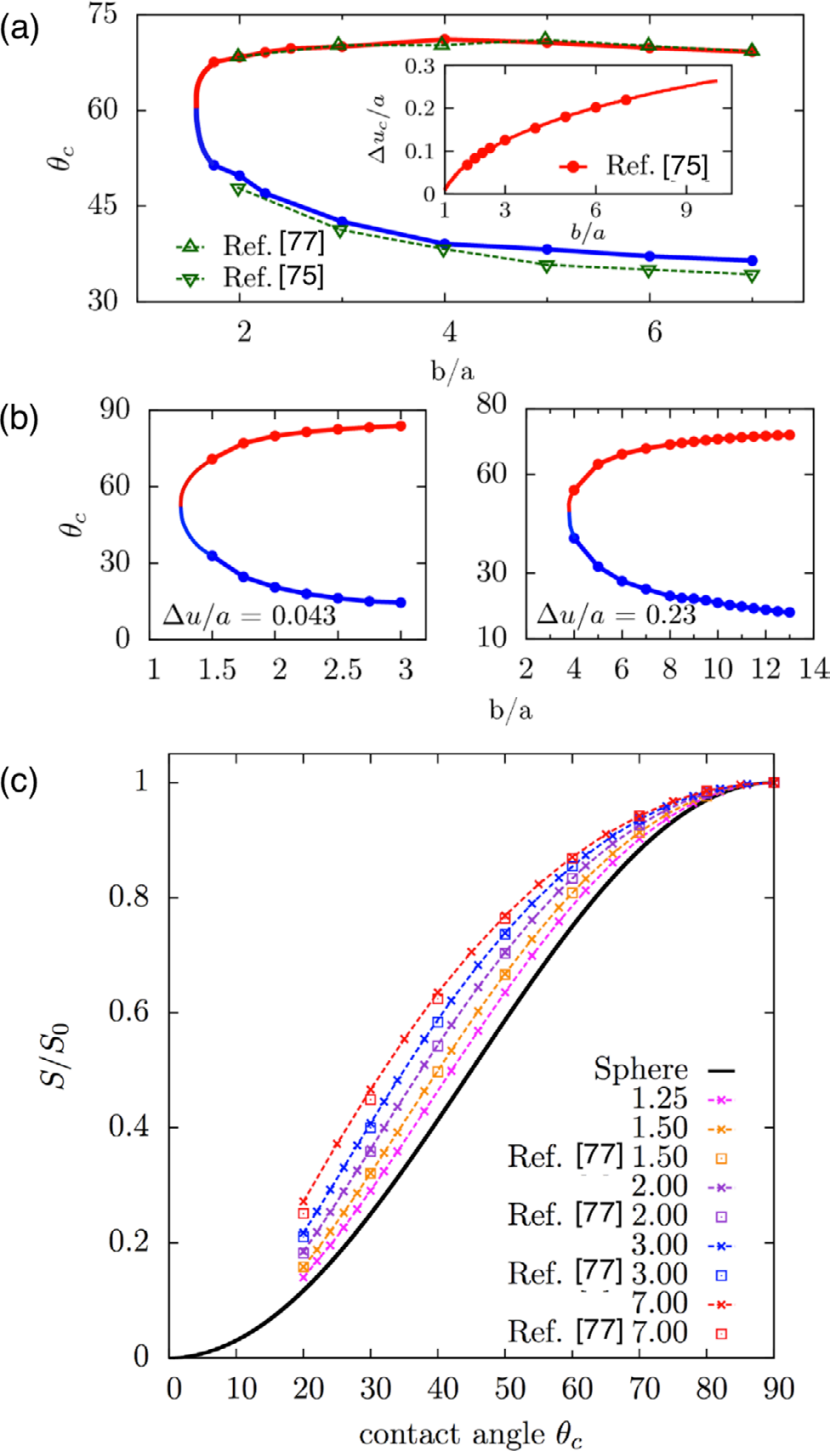
Calculations of contact angles for ellipsoidal particles. (a) Contact angles that correspond to experimentally measured values [74] for  (shown in the inset) for several aspect ratios , see figure 7(c). The upper branch (red) is the solution for large , while the lower branch (blue) is the solution for small . (b) Contact angle branches as function of the aspect ratio for  (left) and  (right). (c) Ratio of projected area enclosed by the contact line for the contact angle  to the projected area enclosed by the contact line for , , for ellipsoidal particles with aspect ratios in the range . The data from [43] is plotted together with numerical data taken from [77]. For a spherical particle,  varies as , as shown by the solid line above. For all contact angles between  and ,  attains higher values for ellipsoidal particles in comparison to the analytical estimate obtained for a spherical particle. Adapted with permission from [43]. Copyright (2014) American Chemical Society.

The experimental determination of contact angles using light microcopy is difficult, because the same interface deformation can be derived for two different contact angles. This ‘contact-angle mystery’ arises because the differential interface height distortion, i.e. , does not show a monotonic increase with the contact angle , see figure 7(d). Due to the non-monotonic profile for ellipsoidal particles, for each value of  there are two possible values for , see figures 8(a) and (b). However, the ‘mystery’ can be resolved by measuring a second quantity, such as the excess area  plotted in figure 8(c), i.e. the ratio of the projected area by the contact line and the projected area at  [74, 77].

In figure 8, results from theoretical contact-angle calculations for ellipsoidal particles with various aspect ratios are plotted together with corresponding experimental measurements of  and with  as function of the contact angle. The lower branch for smaller  has already been reported in [74], the upper branch more recently in [77]. Only with the knowledge of both, experimental values for  and for , the correct branch of contact angle solutions can be singled out. A new technique using electron microscopy provides means to measure contact angles more directly than optical microscopy. This so-called freeze-fracture shadow-casting cryo-SEM (FreSCa) has been used to measure contact angles for wetting of spherical and ellipsoidal micro- and nanoparticles at liquid-liquid interfaces [57].

**Figure 9.**
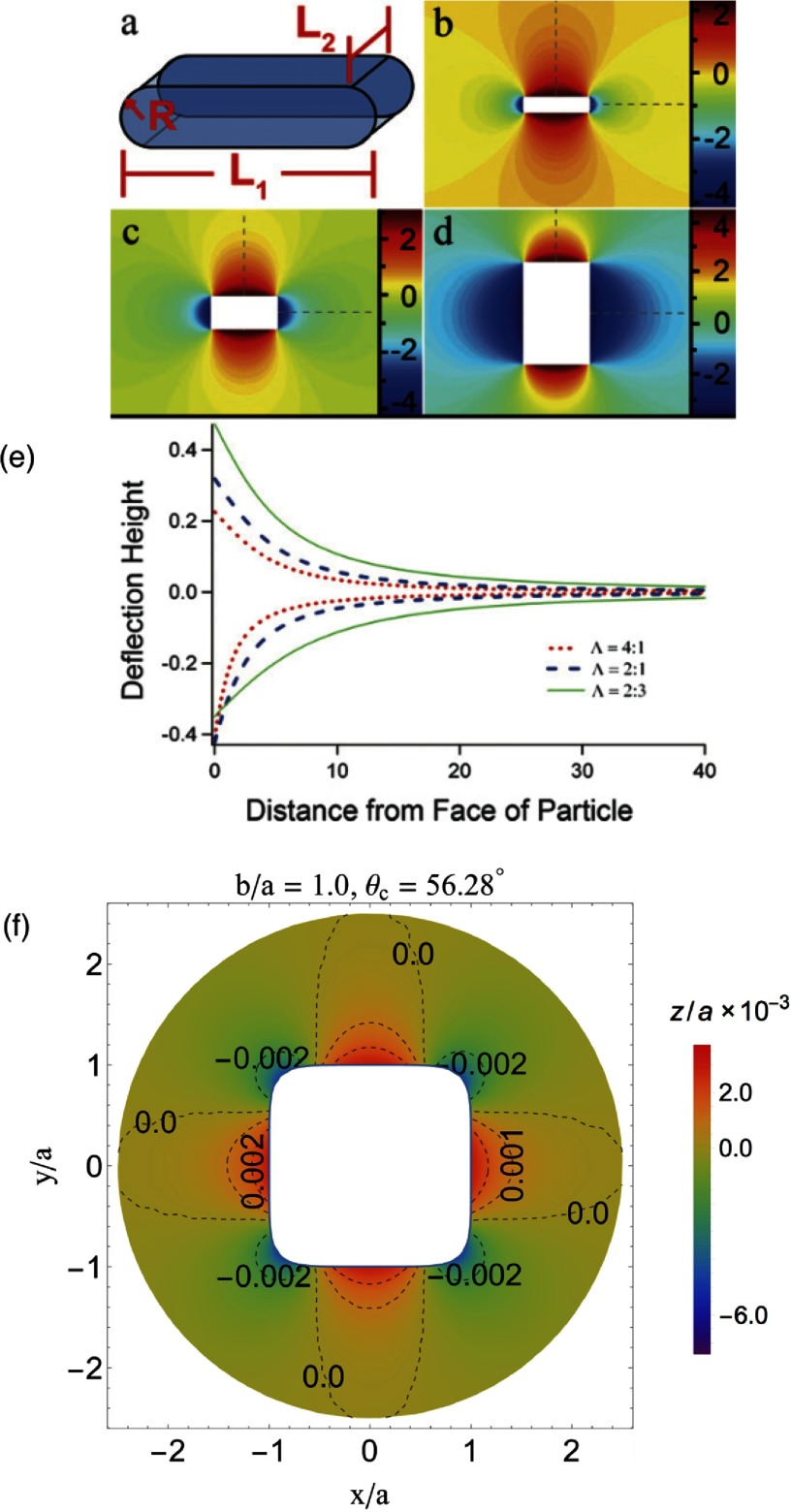

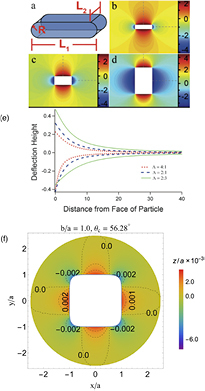

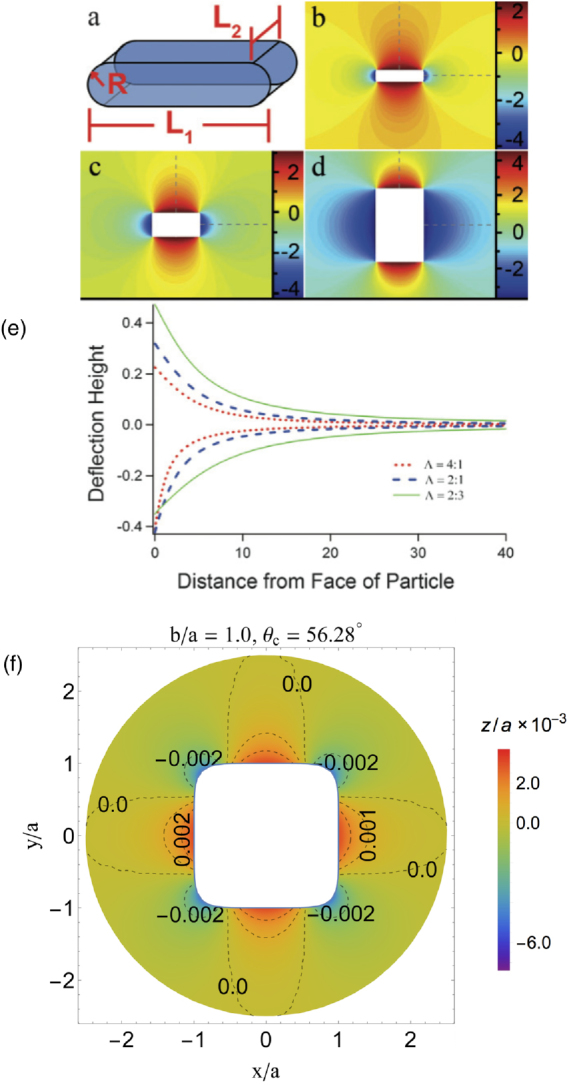
Interface deformations for rounded box-like and cube-like particles. (a) Particles have planar ends of length , curved sides of length , and radius of curvature *R*. Contour plot of the equilibrium configuration of the interface for particles with  and (b)  (c)  (d) (e) Surface shape profile as a function of distance from the middle of the curved faces () and flat faces () for different values of . The heights are scaled by the length scale R. Reprinted with permission from [78] copyright of The Royal Society of Chemistry (f) Deformation profiles of an interface around a particle with Hauser’s cube shape at contact angle  that corresponds to maximum particle-induced interface distortion. Reprinted with permission from [43]. Copyright (2014) American Chemical Society.

For more complex particle shapes, the connection between interface deformation and particle aspect ratio can be different from the case of ‘simple’ elongated particles. For example, figures 9(a)–(e) shows deformations around rounded box-like particles, where rises and dips depend on local particle shape rather than aspect ratio. Cuboidal particles can produce octupolar distortion fields with eight lobes (rises and dips), see figure 9(f). For rounded cylinders, there can even be multiple branches of possible contact angles due to the multiple peaks in the  variation for certain aspect ratios (unpublished results). Table 3 provides an overview of the dominant multipole contributions for interface deformations and corresponding systems, several of them are discussed in more detail in the following.

**Table 3.** Dominant multipole contributions to the interface deformations induced by particles. The interface deformations are determined by particle shape, size, surface functionalization, and external fields.

Deformation	System	References
Monopole	part. in thin films	[79–82]
	part. in grav. fields	[58, 59, 83–88]
Dipole	elong. magn. part.	[89, 92]
Quadrupole	elongated particles	[43, 45, 70, 75]
Hexapole	elong. Janus part.	[93–95]
	cube-like particles	[96–98]
Octupole	cube-like particles	[43, 96]

### 2.3. Field-induced interface deformation

We discuss here gravity, buyoancy, and thin films in part 1 and non-planar interfaces in part 2.

#### 2.3.1. Gravity, buyoancy, and thin films.

The presence of graviational forces leads to floatation forces that cannot be neglected in the regime of large absolute values of the Bond number [59, 83–88, 89]. Particles either sink into the interface if they are heavier than the fluid or float up if they are lighter than the fluid (buyoancy). Gravitational forces therefore induce dominant monopole interface deformations, so-called ‘capillary charges’, see equation (23) [90]. Some fascinating outcomes of the interplay of capillarity and gravity are water striders [99], meniscus-climbing insects [83, 84], and the cheerios effect in cereal bowls [59].

**Figure 10.**
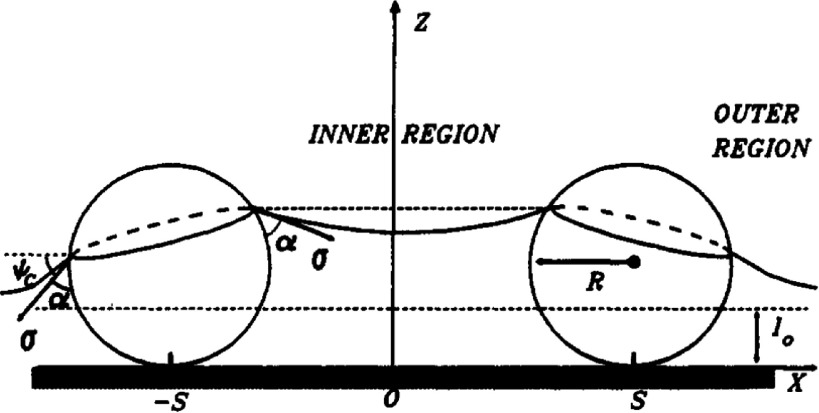

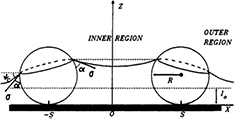

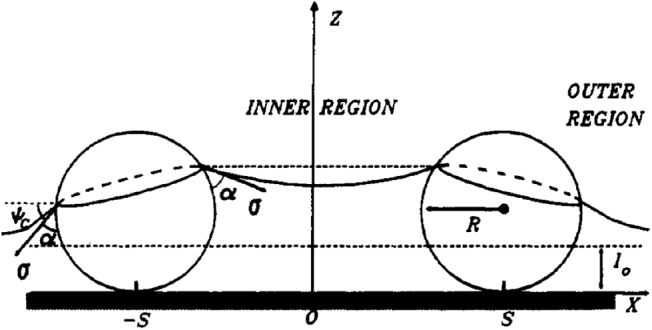
Two spherical particles partially immersed in a liquid layer on a horizontal substrate. The deformation of the liquid meniscus gives rise to interparticle attraction. Reprinted with permission from [79]. Copyright (1992) American Chemical Society.

Monopole interface deformations are obtained as well for particles in thin films, where instead of gravitation the small thickness of the film compared with the particle size induces interface deformations [79, 100], see figure 10. The ideal interface positions on the particle are incompatible with the film thickness, which leads to immersion forces. For a thin film of fluid at a solid interface, the horizontal projection of the immersion force is [79]

**Figure 11.**
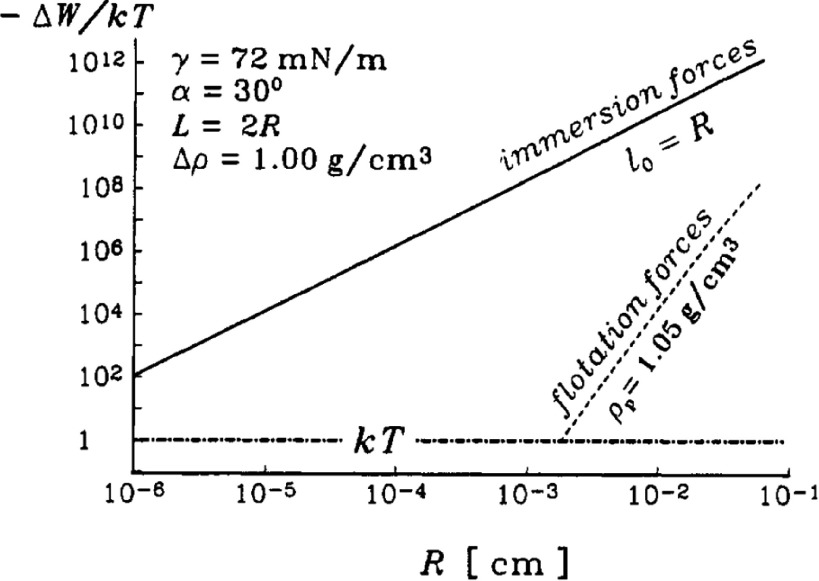

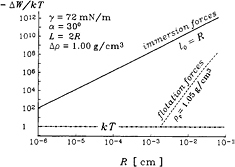

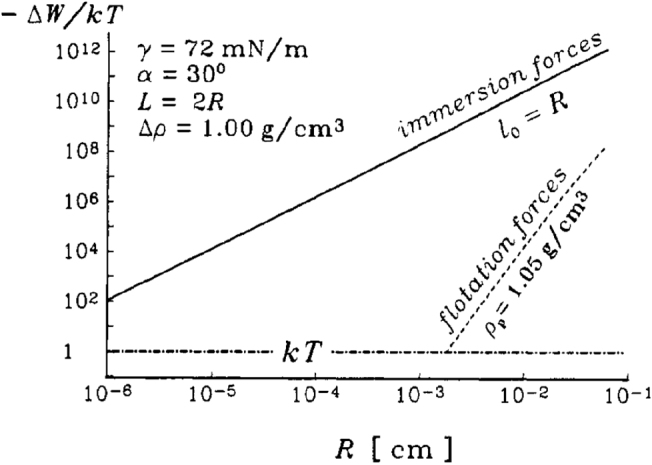
Comparison between immersion and floatation capillary forces between two spherical latex particles in a water film on a glass substrate: dependence of the capillary interaction energy, , on the particle radius, *R*. The distance between the particles is , the contact angle , and the liquid-vapor interface tension . See figure 10 for a sketch of the system. Reprinted with permission from [79]. Copyright (1992) American Chemical Society.

14 with the radius *r*_c_ of the three-phase contact lines at the particles, the mean meniscus slope angle  at the contact line, and the distance *d*_cc_ between the particles. This expression holds for , where  is the density difference between the fluid and the gas. Figure 11 compares the floatation forces between particles with immersion forces for polystyrene latex particles in a water film on a glass substrate; in this example, the floatation forces decrease faster with increasing interparticle distance than the immersion forces.

**Figure 12.**
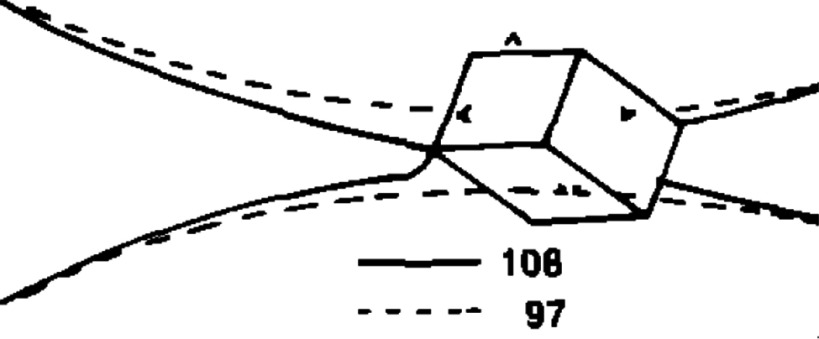

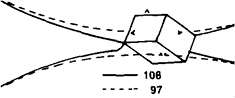

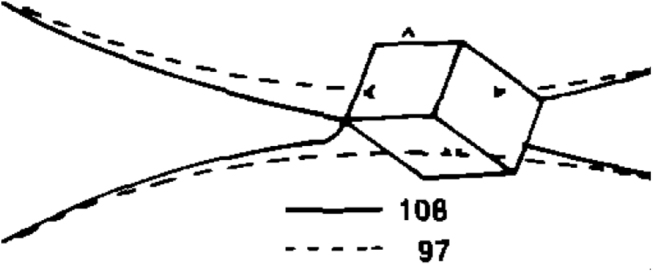
Frames of a recording of an approximately -sized hydrophobic galena particle with  rupturing a film of distilled water. The dashed line indicates the interface position in frame 97, both interfaces join at the sharp edge of the galena particle in frame 108. The time difference between both frames is . After frame 108, the film ruptures. Adapted from [80], Copyright (1982), with permission from Elsevier.

As an instructive example, we discuss froth floatation as an application in more detail [101]. Here, grains of one solid are carried away with the froth, while grains of a different solid sink to the bottom of the floatation system. The selective adsorption of a collector chemical onto a mineral in a flotation plant determines—among others—the attachment of the mineral to bubbles used for separation. The stability of the froth with the particles depends mainly on the properties and the amounts of particles [80]. The so-called capillary pressure due to the liquid that drains from the film because of gravity measures the pressure when the film ruptures. This critical pressure increases if particles stabilise the film, e.g. spherical particles with contact angles far below , and decreases if particles destabilise the film, e.g. particles with sharp edges. Whether a particle stabilizes or destabilizes a film furthermore depends on the orientation that the particle assumes at the interface [102], see section 2.4. Figure 12 shows how a thin film ruptures due to the presence of a non-spherical sharp-edged particle.

#### 2.3.2. Curved interfaces.

**Figure 13.**
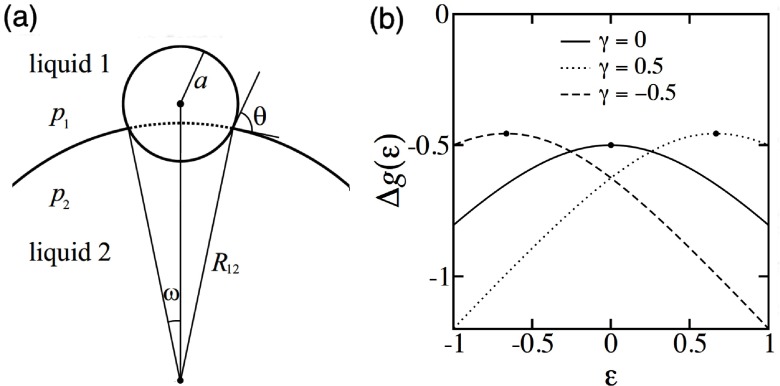

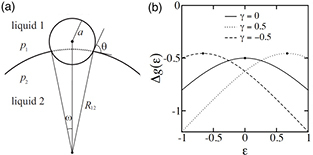

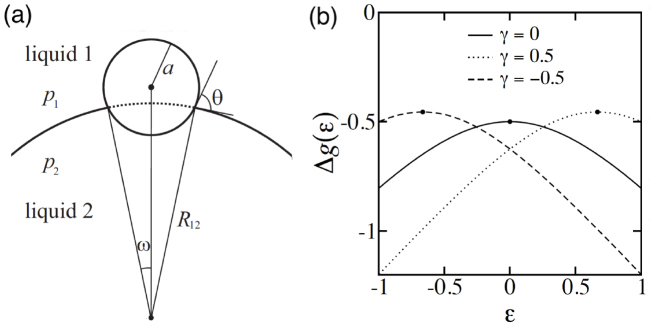
Spherical particles at curved interfaces. (a) Schematic of spherical colloids of radius *a*, trapped at a droplet. (b) Theoretical estimates of normalized interface energy as function of droplet curvature. Reprinted with permission from [64]. Copyright (2006) AIP publishing.

Particles at curved interfaces experience forces not only due to the presence of the interface, but also because of the Laplace pressure. Curvature gradients of the interface lead to lateral forces, and curved interfaces modify interface-mediated interactions between particles [61, 64, 71, 72, 103, 104], see figure 13; vice versa, particles at high densities can induce spontaneous interface curvature [105]. Mean curvature *H* and deviatoric curvature  determine the trapping energy [104]

15 where *E*_F_ is the trapping energy at the flat interface, see equation (4).

For a spherical colloid of radius *a* on a spherical droplet of radius *R*_d_ (i.e.  and ), this implies , which is the interface curvature-induced contribution. This term is evaluated by considering contributions due to both the interface energies and the work done against the Laplace pressure. A similar estimate for the trapping energies attempted previously in [106] without including the work done by the Laplace pressure resulted in the correction term to be . This result is consistent with equation (15) when the contribution due to the pressure term, , is added to it [104].

For a minimal surface with mean curvature , such as for a catenoidal interface shape, the correction term compared with the flat interface depends only on the deviatoric curvatue . The trapping energy of a colloidal particle on a catenoidal minimal surface is thus .

On an interface with varying curvature, the lateral force on the trapped particle is given by [104]

16

Thus, spherical particles on interfaces with curvature gradients experience forces depending on both the absolute values and the gradients of the mean and deviatoric curvature to move towards regions of high mean and deviatoric curvatures (and therefore also high Gaussian curvatures because ). Recent experiments of microspheres on interfaces with different curvatures have evaluated these forces to be of the order  fN for silica beads with radii  at oil–water interfaces () with contact angle  and, for interface, curvatures of the order of  [35].

Theoretical estimates of capillarity at curved interfaces—which neglect pinning—suggest that capillary forces for particles that are smaller than the capillary length

**Figure 14.**
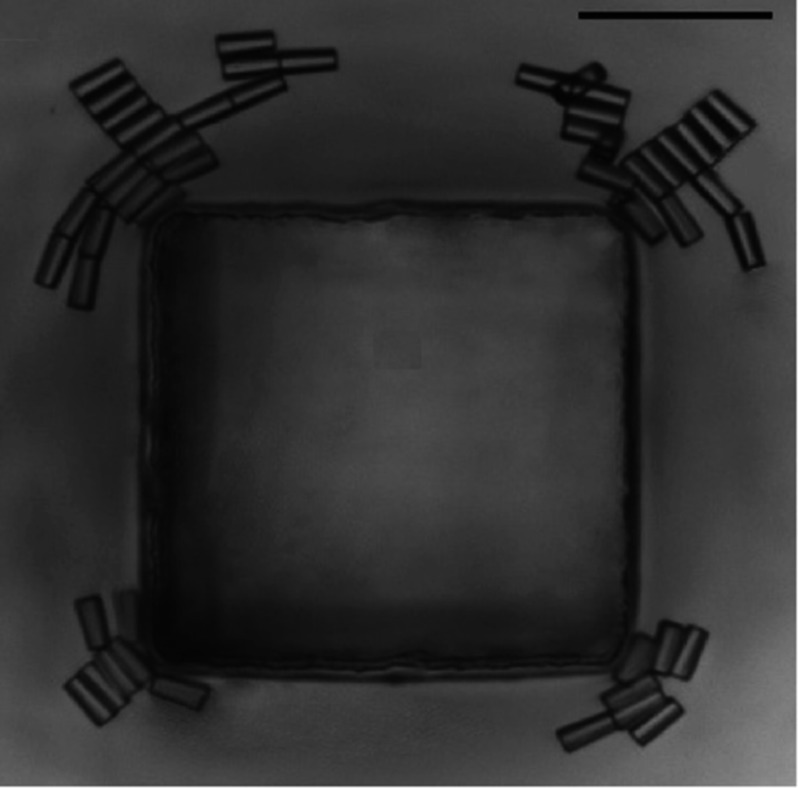

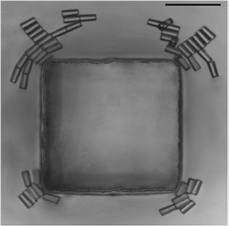

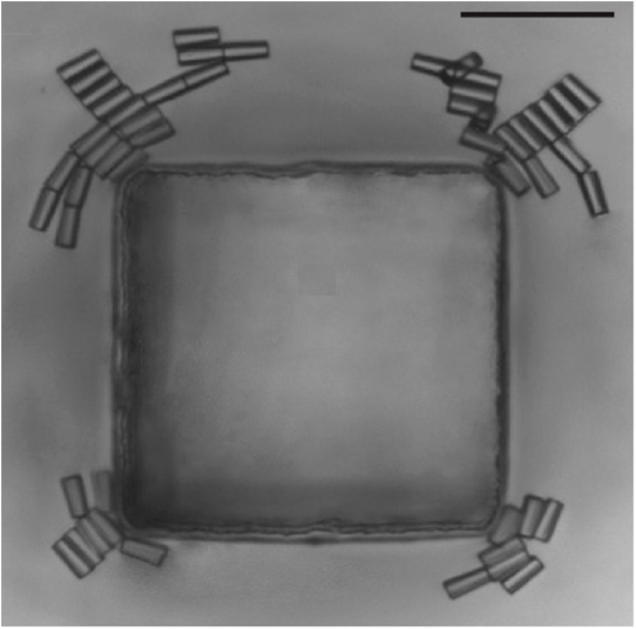
Particle migration near a square micropost. Complex structures formed by aggregated particles near the corners of the micropost. The scale bars corresponds to . Reprinted with permission from [103].

17 where  is the density of the fluid, are proportional to both *a*^4^ and the gradient of the Gaussian curvature,  [35]. This has been verified in experiments where large microposts induce a curvature gradient and cause smaller particles to migrate along the deformed interface to assemble in regions of high Gaussian curvature, see figure 14.

Experimental evidence further suggests that capillary migration on curved interfaces can be enhanced by contact-line pinning. Contact-line undulations, which lead to finite local interface curvatures, induce capillary forces of the order – , which are much larger compared with the weak forces due to capillarity for perfectly smooth spherical particles. The experiments demonstrate that rough microspheres, microdisks, and rod-like particles move along deterministic trajectories to regions of maximum deviatoric curvature [71, 72, 103]. Non-spherical particles do not only migrate translationally by sensing the background interface curvature, but also orient themselves to align their long axes, such that the excess area is minimized. For example, cylindrical particles reorient either parallel or perpendicular to the groove as the interface curvature changes from concave to convex [107].

### 2.4. Non-spherical shapes, surface heterogeneities, and particle orientations

For non-spherical particles, the orientation of the particles at interfaces, as well as particle shape, size, and surface properties have to be taken into account to determine adsorption energies and interface deformations. Cube-like particles can be oriented in a corner-top or face-top orientation depending on the contact angle. Elongated particles in a magnetic field or elongated Janus particles in their stable orientations can be oriented with their long axes tilted with respect to the interface.

#### 2.4.1. Cube-like particles.

**Figure 15.**
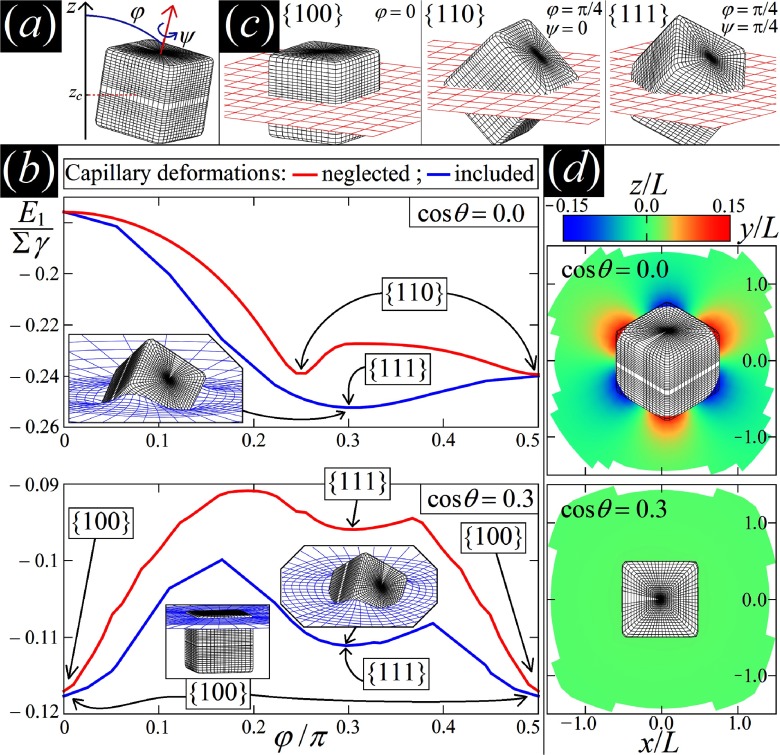

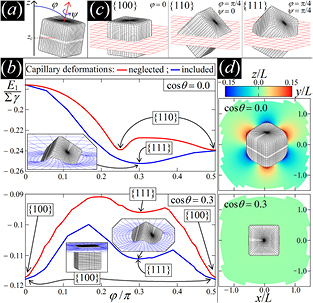

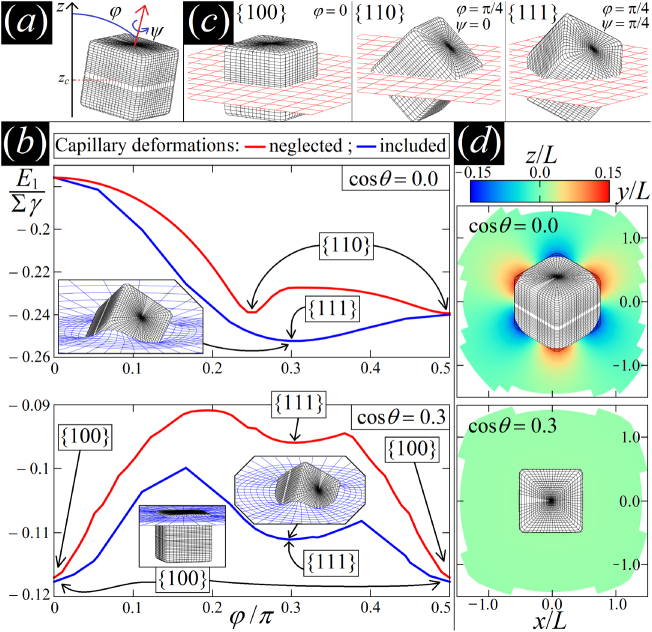
Orientations of cube-like particles at interfaces. (a) Configuration of a cubic particle at a fluid-fluid interface. (b) Adsorption energy of a single cube (side L) at a fluid-fluid interface, in units of the product of the cube surface area and the interface tension, , minimized over the center of mass height and the internal Euler angle *ψ* as function of the polar angle *ϕ* for contact angles  and . The blue and red lines include and neglect capillarity, respectively. The labels , , and  indicate the cube’s orientation in each minimum of the energy. The insets show, for the equilibrium configurations, a 3D view of the interface shape (blue grid) close to the particle (black grid), as calculated by our method. (c) 3D illustration of the , , and  orientations of a cube, where the red grid represents a plane parallel to the flat interface. (d) Contour plots of the deformed-interface height profile for the global minimum-energy configuration of the cube. For , a hexapolar deformation emerges, while for , the interface is essentially undeformed. Reprinted with permission from [97]. Copyright (2016) by the American Physical Society.

One way to estimate the adsorption energy of a particle at an interface is to calculate the area of the fluid interface that is ‘cut out’. This estimate can be exact if the interface tensions between particle and both phases are equal. For a particle that is symmetric with the interface acting as a mirror plane, the surrounding interface remains planar. However, in general particle-induced deformations of the surrounding interface can change adsorption energies and stable particle orientations even qualitatively. Figure 15(a) sketches the various angles by which a cube-like particle can be rotated. Figure 15(b) shows deformation energy calculations for the contact angles  and . For  the flat-interface approximation predicts the edge-top orientation to be globally stable, while accounting for the deformation of the surrounding interface the corner-top orientation is found to be globally stable.

Figure 15(c) shows specific orientations for a cube-like particle at an interface. For a contact angle , the globally stable state is the face-top orientation, while for  the globally stable state is the corner-top orientation. Figure 15(d) shows the predicted interface deformations for both cases using the same scale: hexapolar for the corner-top orientation and almost planar for the face-top orientation. Capillary interactions between the cubes are therefore expected to be much stronger in the corner-top orientation compared with the face-top orientation. For particles with complex shapes, such as cube-like particles, a multitude of kinetically stabilised orientations and corresponding multipolar interface deformations is expected to be observed in experiments.

#### 2.4.2. Janus particles.

**Figure 16.**
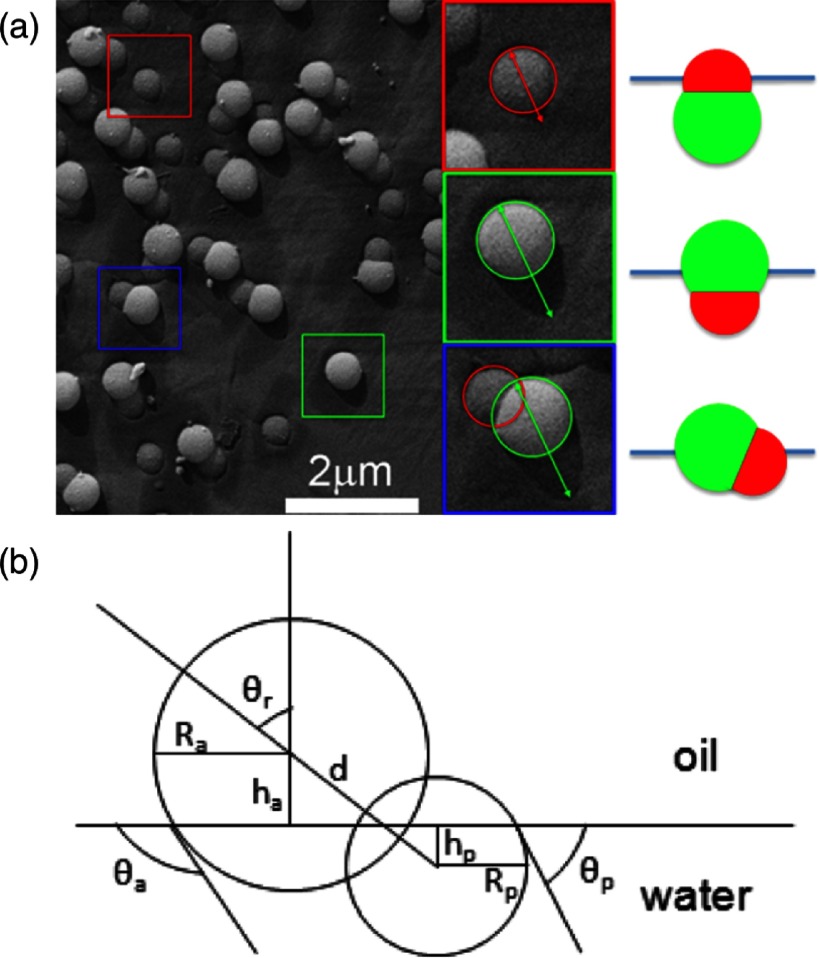

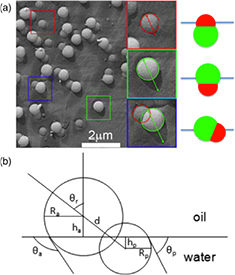

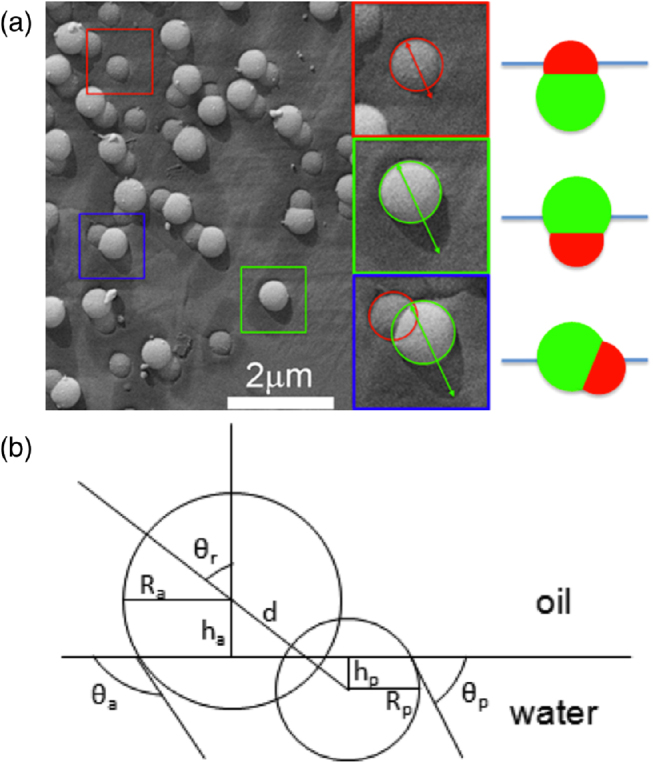
Amphiphilic dumbbell particles at an oil–water interface. (a) In experiments, three dumbbell orientations are found: only the hydrophilic sperical particle intersects the interface, only the hydrophobic spherical particle intersects the interface, and both spherical particles intersect the interface. The circles and arrows indicate the size of the particle crossection at the interface and the shadow length obtained by freeze-fracture, shadow-casting (FreSCa) cryo-scanning electron microscopy, that can be used to measure the contact angle. (b) Schematic diagram highlighting the geometry of the dumbbells at a liquid-liquid interface. Reprinted with permission from [73]. Copyright (2014) American Chemical Society.

**Figure 17.**
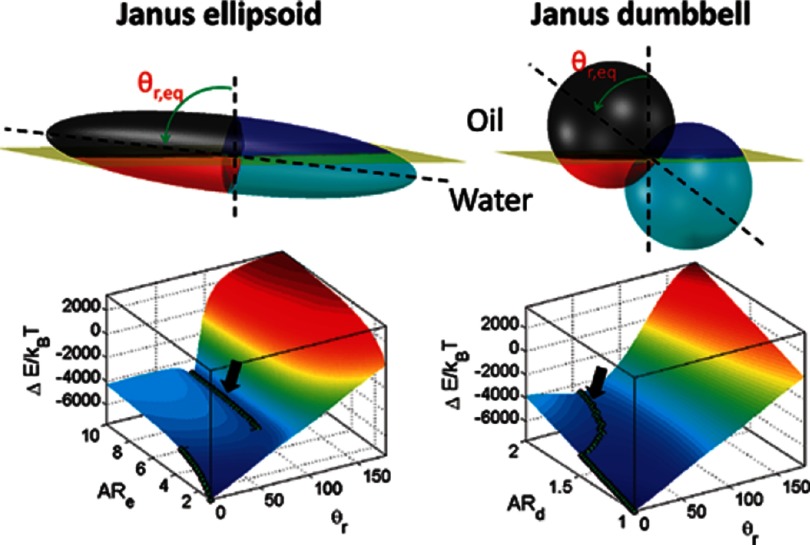

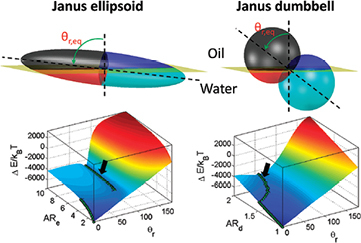

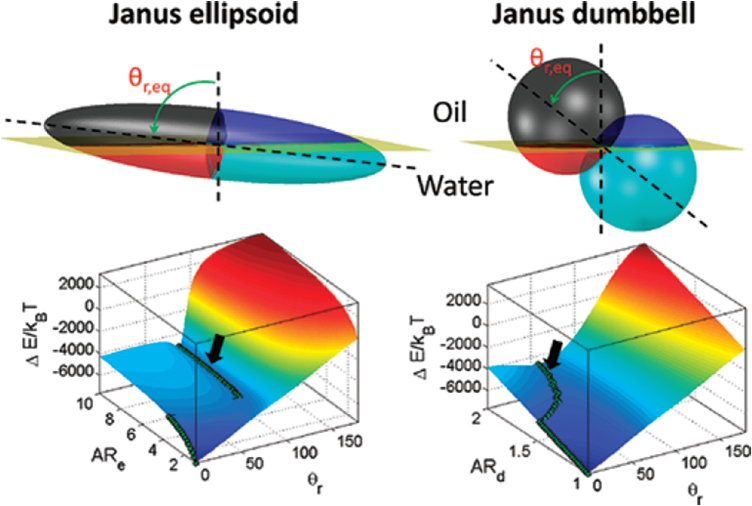
Janus ellipsoid and Janus dumbbell at an interface with corresponding energy landscapes for particle orientations. While the dumbbell smoothly transitions to the perpendicular orientation with increasing aspect ratio, the ellipsoid jumps from a small tilt angle to the perpendicular orientation beyond a critical aspect ratio. Reprinted with permission from [108]. Copyright (2012) American Chemical Society.

A simple non-spherical particle with a stable tilted state is a Janus dumbbell, which consists of two spherical particles made from different materials, see figures 16 and 17. These particles do not deform planar interfaces, because the contact line around each of the spherical particles is a circle. Figure 16(a) shows the experimentally observed orientations for dumbbell particles that consist of an hydrophobic spherical particle of radius *R*_a_ and a hydrophilic spherical particle of radius *R*_p_. In their lowest-energy orientation, the spherical particles intersect the interface at different heights. The tilt angle of the dumbbell in the lowest energy state is [73]

18 see figure 16(b). Here  is the contact angle at the polar particle,  is the contact angle at the apolar particle,  is the relative size of the two lobes, and  is the aspect ratio.

With decreasing aspect ratio, the tilt angle of the Janus dumbbell increases. For aspect ratio , only one of the spherical particles is in contact with the interface. However, also for cases where the tilted orientation of the dumbbell is predicted to have the lowest energy, a substantial fraction of particles are found experimentally in one of the kinetically stable orientations, where only one of the spherical particles intersects the interface [73]. The lowest-energy orientation, where both spheres are at the interface, coexists with two kinetically stable orientations where only one of the spheres is at the interface. Because the energy for an orientation where only one spherical particle is in contact with an interface does not depend on the tilt angle, an arrest in such a state could be caused by surface roughness. In these trapped states, the particles can therefore reorient to the tilted orientation only by diffusion.

**Figure 18.**
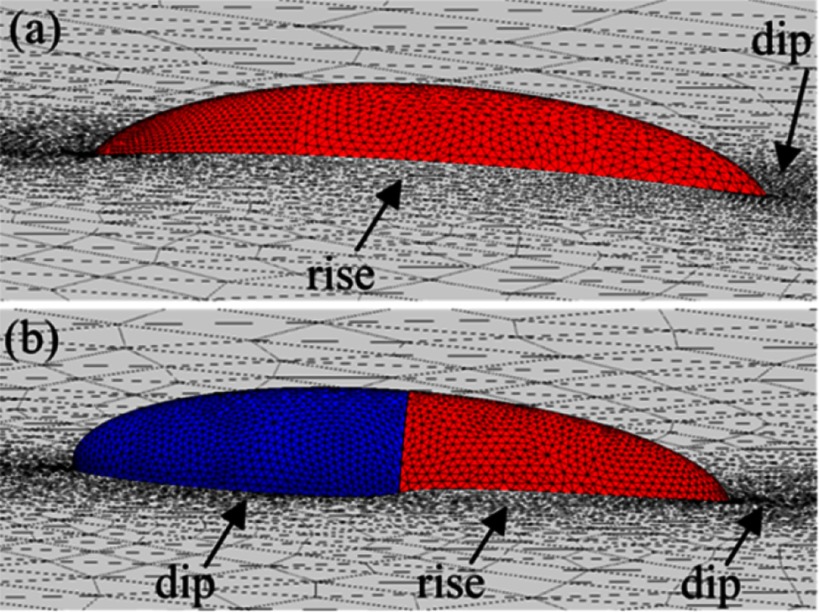

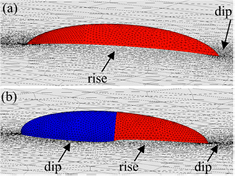

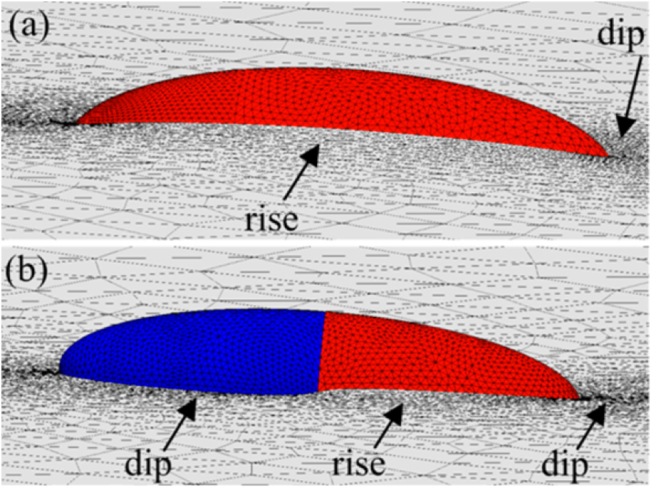
An ellipsoidal particle with (a) homogeneous surface properties and (b) a Janus ellipsoid at an interface. Around an ellipsoid with homogeneous properties and contact angles below  the interface is suppressed at the tips and pulled up at the sides of the particle. Around a Janus ellipsoid the interface can experience a hexapolar deformation. Reprinted with permission from [93]. Copyright (2013) American Chemical Society.

Unlike Janus dumbbell particles, Janus ellipsoidal particles that are half hydrophobic and half hydrophilic do not show a continuous transition of their the orientation between a tilted long axis and a perpendicular long axis to the interface, see figures 17 and 18. Energy landscapes for various orientations of an ellipsoidal and a dumbbell particle are shown in figure 17. The lowest energy states for different aspect ratios are indicated in the figure using green symbols; the discontinuous transition in the particle orientation is clearly visible. The tilted orientation is only stable above a threshold aspect ratio and the orientation of the particle orientation jumps to the perpendicular orientation below this aspect ratio [108]. These energies for various particle orientations have been calculated numerically, under the assumption of a flat interface surrounding the particles.

**Figure 19.**
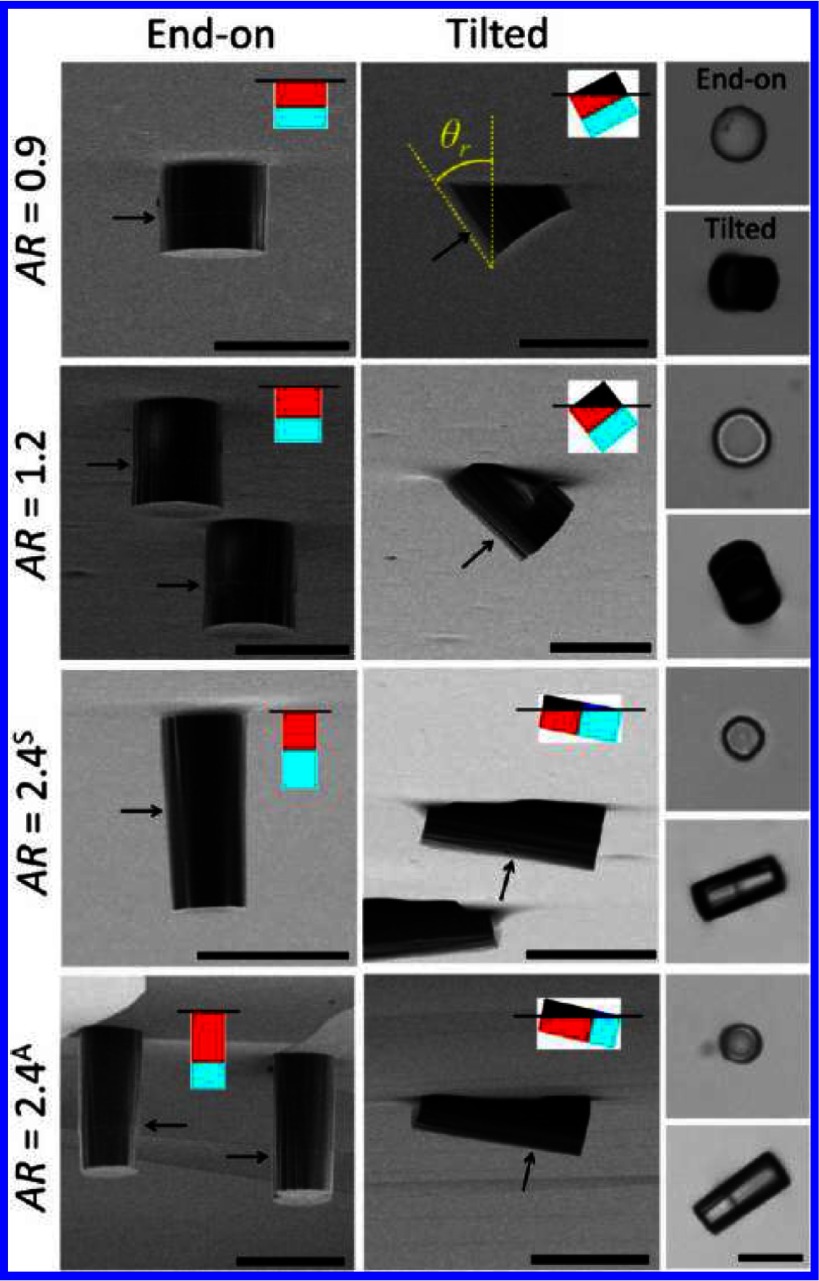

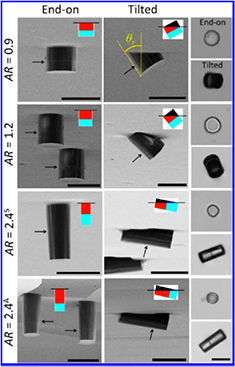

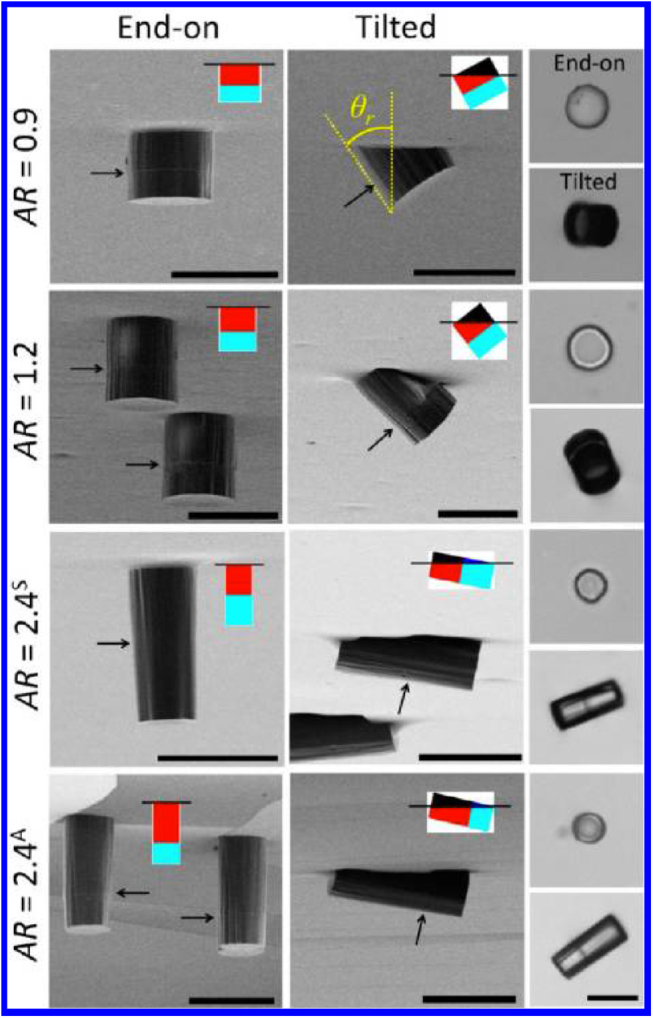
Configurations of double-hydrophilic Janus cylinders at the air-water interface. The first and second columns are SEM images of Janus cylinders embedded in PDMS slabs prepared by the gel trapping method. Arrows indicate the location of the wettability separation line. The schematic representations show the side view of particle configurations at the air-water interface where four colors represent four different particle-fluid surfaces: weakly polar surface in air (black), weakly polar in water (red), strongly polar in air (blue, rarely shown), and strongly polar in water (cyan). The third column shows the corresponding optical microscopy images. The scales bars are . Reprinted with permission from [94]. Copyright (2013) American Chemical Society.

Whereas for ellipsoidal particles with homogeneous surface properties the deformations of the surrounding interface are quadrupolar with a dip at the tips and a rise at the long sides for contact angles below , see section 2.2, the deformations of the interface around Janus ellipsoids can show both a dip and a rise along their long sides [93], see figure 18. Similar interface deformations have been observed for double-hydrophilic Janus cylinders with aspect ratios , , and  [94], see figure 19. The cylinders are found in kinetically trapped end-on orientations as well as globally stable tilted orientations. The cylinders have asymmetric hydrophilicity unlike most studies where the Janus particles have amphiphilic properties [94]. In their tilted orientations, they induce hexapolar interface deformations that lead to capillary interaction. Complex particle assemblies have been observed due to the multi-lobed deformations and the non-determininistic assembly.

#### 2.4.3. Particles in magnetic fields.

**Figure 20.**
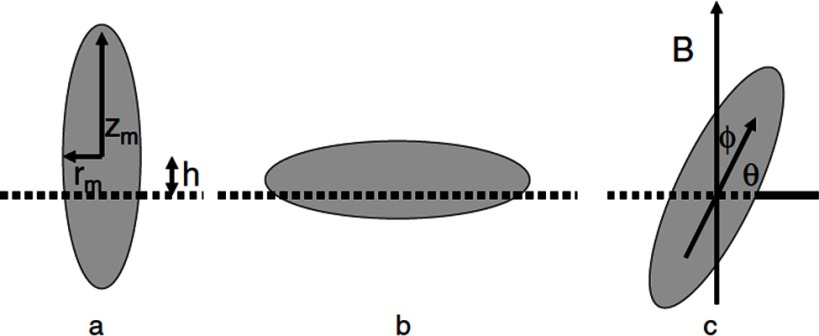

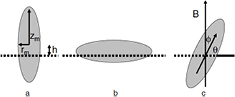

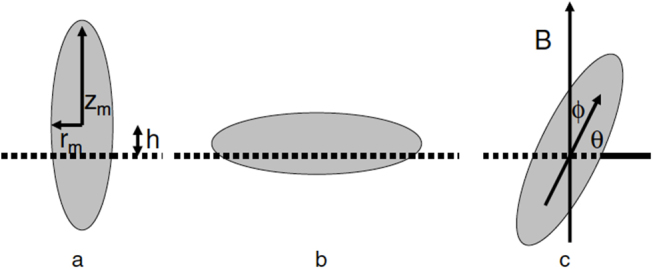
Sketch of an ellipsoidal particle adsorbed at a liquid-liquid interface in (a) vertical, (b) horizontal, and (c) tilted orientation. The variables defining the geometry and orientation of the particle are discussed in the text. The direction of the magnetic field is indicated by *B*. Reprinted from [41]. © IOP Publishing Ltd. All rights reserved.

An external magnetic field can be used to tune the orientation of prolate ellipsoidal magnetic particles at fluid interfaces [41, 92, 109], see figure 20. The field tends to align the magnetic dipole with the direction of the applied magnetic field,

19 where  and  are the dipole and the field, respectively. The angle *ϕ* indicates the particle orientation and  represents the field-dipole strength. The total free energy of the particle at the interface, neglecting particle-induced interface deformations, can therefore be written as

20 where *S* is the area of the interface ‘cut out’ by the particle, and  is the area of the particle in contact with the vapor/the second liquid.

**Figure 21.**
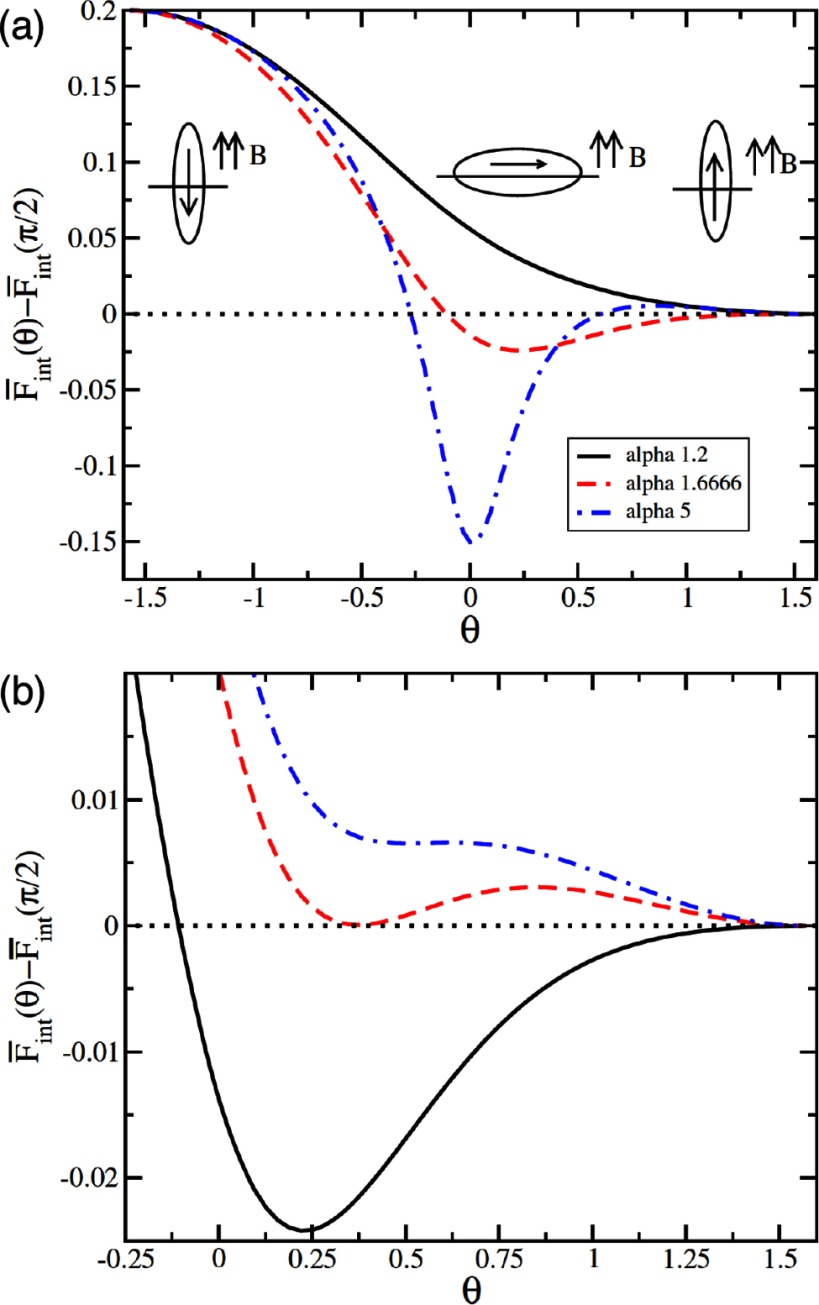

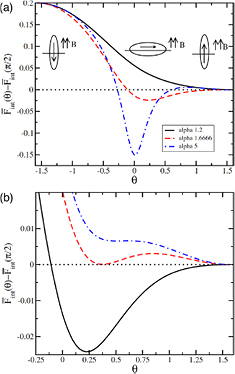

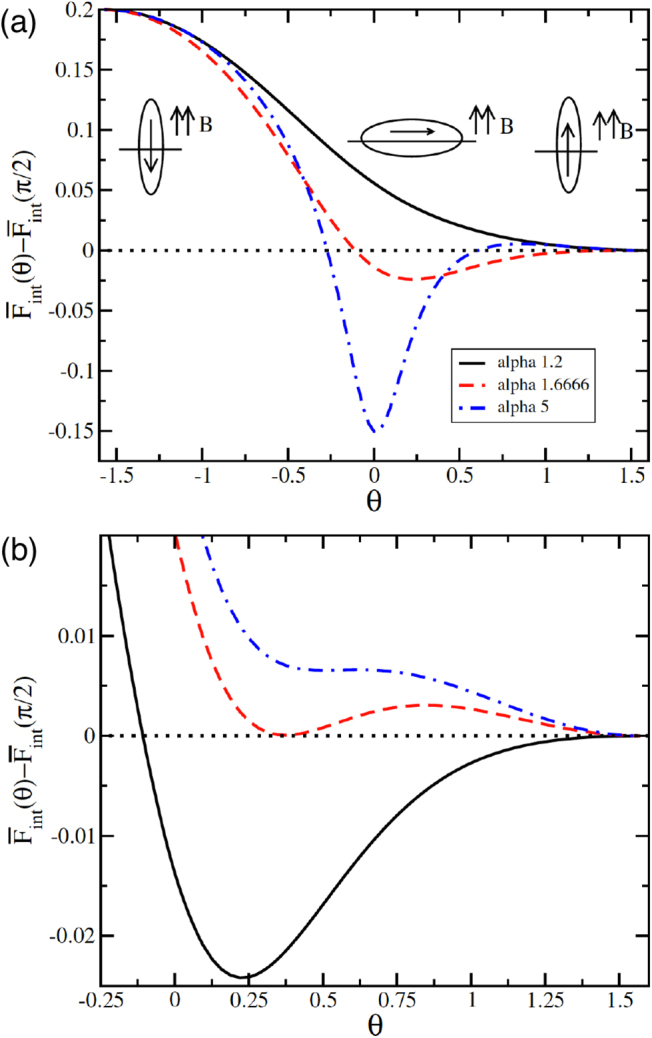
Energy of a magnetic ellipsoidal particle at an interface as function of its orientation. (a) The results for particle aspect ratios , , and  correspond to a representative field strength . (b) Energies for an ellipsoidal nanoparticle with aspect ratio  for different external field strengths , , and . Reprinted from [41]. © IOP Publishing Ltd. All rights reserved.

If the field is oriented normal to the interface and if the direction of the permanent magnetic dipole coincides with the major axis of the ellipsoid, the analytical estimation of the tilt energy predicts that at a critical field strength the particle ‘jumps’ from a tilted orientation to a vertical orientation. Here,  and  correspond to vertical and horizontal particle orientations, respectively. In figure 21, the energies of magnetic prolate ellipsoidal particles are shown as function of their orientation angle for various aspect ratios and field strengths. For particles with aspect ratio  and length of the minor axis a  =  , the energy barrier between vertical and tilted states can be several hundred , such that the particles are trapped in one of both orientations.

**Figure 22.**
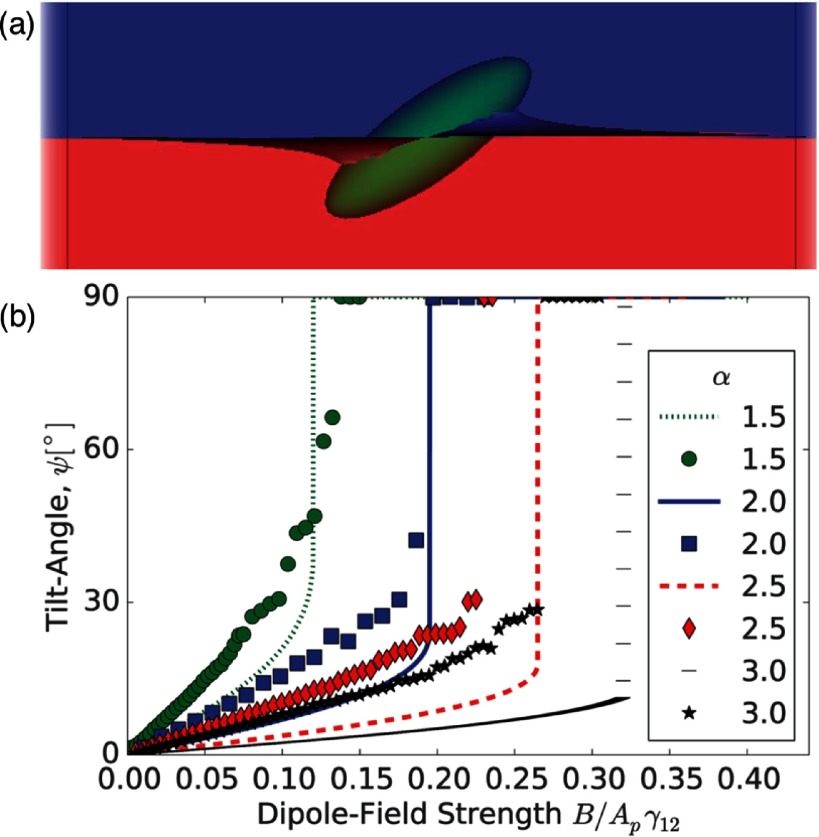

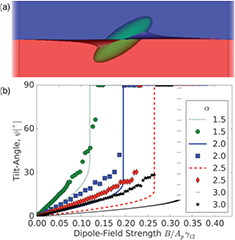

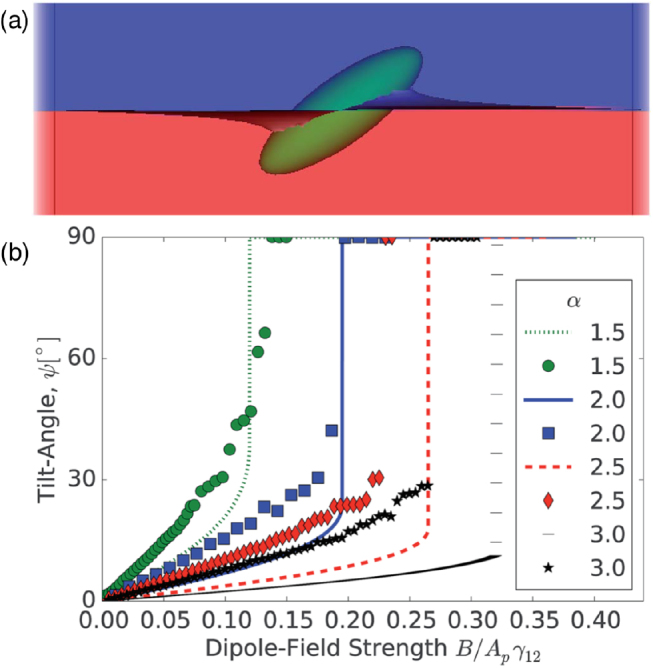
Magnetic ellipsoidal particles in an external field. (a) Simulation snapshot of an ellipsoidal particle with aspect ratio  unter the influence of a dipole field . (b) Comparison of the tilt angle of the particle obtained from the analytical, planar-interface approximation in [41] and the numerical data in [91]. Although the numerical data qualitatively confirms the prediction of a discontinuos transition, there are quantitative deviations in particular at higher aspect ratio. Reprinted from [89] with permission from The Royal Society of Chemistry.

Lattice-Boltzmann simulations have been used to investigate the orientations of magnetic ellipsoidal particles in external fields including the deformations of the surrounding interface [91]. The simulations show that the interface deformations around the particles significantly affect the tilt angles for a given dipole strengths, altering the properties of the reorientation transition. Figure 22 shows a simulation snapshot of the deformed interface and a plot of the tilt angle for various field strengths together with the analytical approximation discussed above. The simulation snapshot shows that the interface deformations remove more interface than in the planar-interface approximation, which lowers the free energy. Deviations from the approximate result are found mainly for high aspect ratios.

The discontinuous transitions allows the switching of the particle orientations using external magnetic fields, which could find applications for instance using its dynamically-tunable optical properties for electronic readers [110]. Furthermore, also the dipolar interface deformations can be switched on and off and thus lead to switchable capillary interactions between particles adsorbed at fluid interfaces.

### 2.5. Long-range interface-mediated interactions

**Figure 23.**
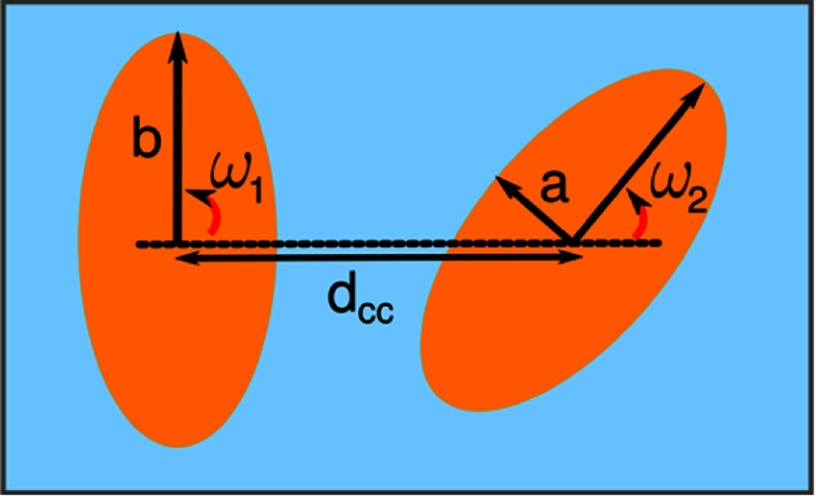

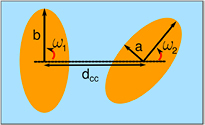

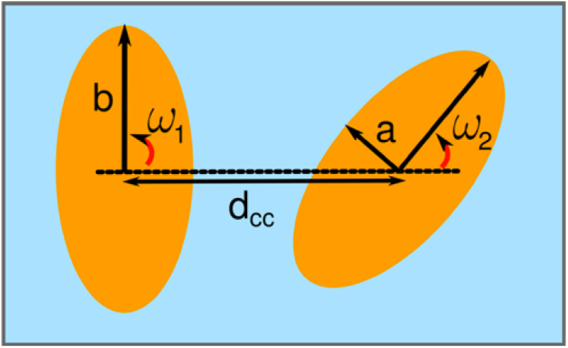
Two ellipsoidal particles at an interface. The angles  and  indicate the orientation of the particle 1 or 2, respectively, with respect to the vector joining the centers of the two particles. The center-to-center distance is given by  for a particle of aspect ratio . Reprinted with permission from [43]. Copyright (2014) American Chemical Society.

Overlaps of interface deformations around particles lead to interface-mediated interactions, also called (lateral) capillary forces. Capillary interaction energies between two particles, see figure 23, can be calculated based on the difference between the changes of the fluid-vapor interface area for placing two particles at given center-of-mass separation *d*_cc_ and for placing two isolated particles at a planar interface. The corresponding capillary interaction energy is [67]

21 where  is the change of the fluid-vapor interface area around the interacting particles, and  and  are the changes of the interface areas around the single particles. Interface-mediated interactions follow power laws in the far field and are thus long-ranged. In the near field, the interactions strongly depend on the particle shapes. Because the dynamics occurs in the Stokes regime for small capillary numbers, see equation (7), the particle velocities are proportional to the interaction forces and can therefore be extracted for example from videos. Analytical solutions for the interaction forces are available in the far field, while numerical calculations have to be used in the near field.

#### 2.5.1. Far-field interactions.

For large distances between particles, the deformation field of particle  can be assumed to be small at the position of particle  and vice versa. The capillary interaction energy can then be calculated using the superposition of the interface deformations around the single particles [58, 67, 111]

22 where *h*_A_ and *h*_B_ are the interface height deformation fields due to particle  and , respectively. The lowest multipole contribution for a given system, see table 3, dominates the interaction.

For sufficiently large particles for that gravity or buyoancy have to be considered, the capillary forces act between two monopole interface deformations [58]

23 with the capillary length defined in equation (17), the mass density  between the upper and the lower phase, and the capillary charges . The angle  is the slope of the interface at radius *r*_*i*_, the radius of the contact line. For ,  with , particle mass density , fluid mass density , and upper fluid/gas mass density .

Stamou *et al* first presented an analytical result for the pair potential between two quadrupolar deformation fields in polar coordinates [67]. In this quadrupolar approximation, the energy between two ellipsoidal particles is

24 see figure 23. The maximal contact line deformations  depend on the contact angles and the aspect ratios of the particles, see figure 7. The angles  and  measure the orientations of the particles with respect to the line joining their centers of mass. For  the particles are in side-by-side (S-S) orientation, for  in tip-to-tip (T-T) orientation. Equation (24) predicts attraction for S-S and T-T orientation, and repulsion for tip-to-side (T-S) orientation with  and . Interestingly, the magnitudes of the interaction potentials are equal in S-S, T-T, and T-S orientation for equal center-of-mass distances. Kralchevsky *et al* extended the multipole approach to multipoles of arbitrary orders [105]. Whereas for weak deviations from circular contact lines a polar multipole expansion is most appropriate, for ellipsoidal particles with higher aspect ratios the appropriate choice are elliptical multipoles [75].

**Figure 24.**
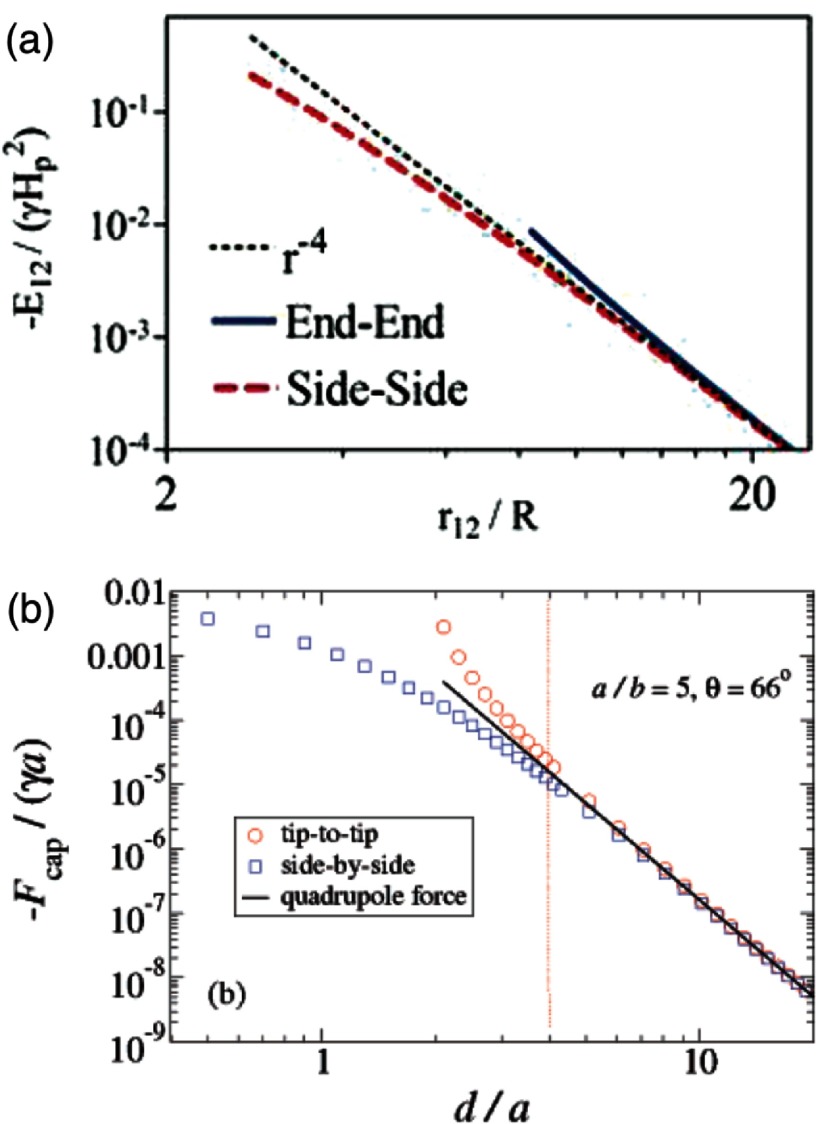

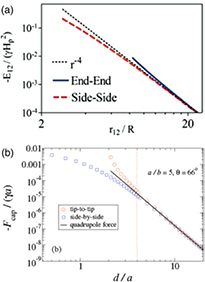

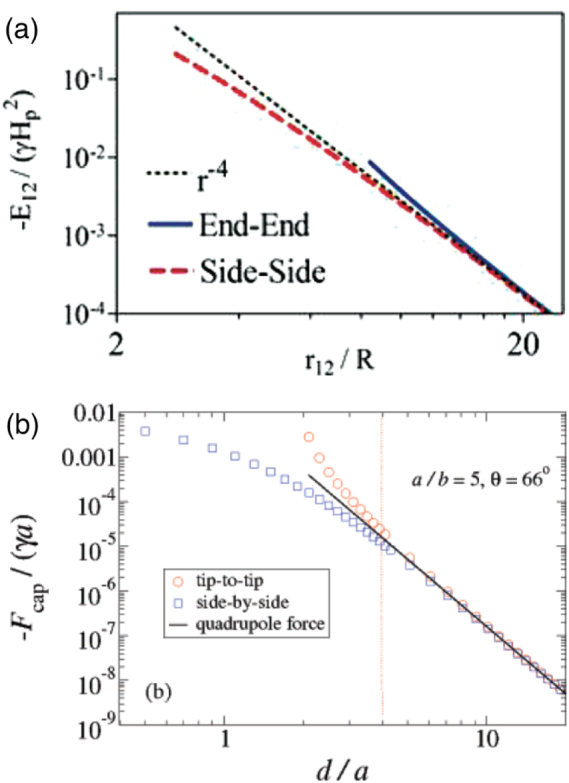
Interaction energies and capillary forces. (a) Interaction energy between two elliptical quadrupoles for two neighbouring cylindrical particles approaching each other in side-by-side and end-to-end orientation. Reprinted with permission from [65]. Copyright (2010) American Chemical Society. (b) Capillary forces evaluated for two similar ellipsoidal particles using superposition of elliptical quadrupoles for aspect ratio 5 and contact angle 66° for varying inter particle separation. Reprinted with permission from [75]. OA CC BY 4.0.

Figure 24 shows interaction potentials for cylindrical particles and forces between two ellipsoidal particles using the superposition approximation. For large distances, the expected dependences of potential and force with the interparticle distance  for elongated particles,  and , are observed for both cylindrical and ellipsoidal particles [65, 75]. Deviations are observed for small interparticle distances, which is discussed in section 2.5.2.

#### 2.5.2. Near-field interactions.

Elongated particles that touch each other have lower bond energies than predicted by the quadrupolar approximation in side-by-side orientation and higher bond energies in end-to-end orientation, see figure 24. Capillary self-assembly is determined by the contact interactions between the particles, therefore near-field interactions are important for all many-particle systems. Uncharged ellipsoidal particles at low Bond numbers preferentially align in side-by-side orientation [45]. The higher stability of the side-by-side orientation over the tip-to-tip orientation is obvious from equation (24) and is qualitatively unchanged by the deviation from the ideal quadrupolar approximation in the near field.

**Figure 25.**
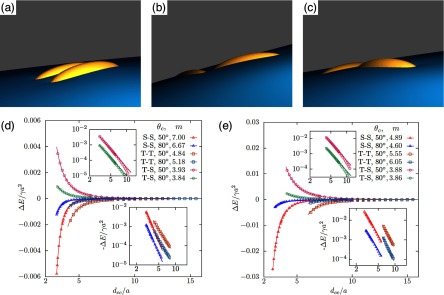

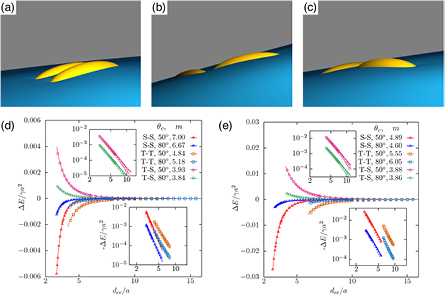

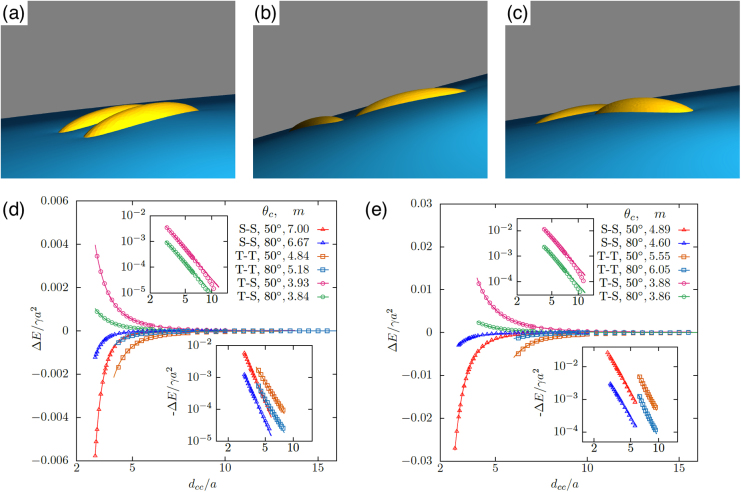
Interface deformations around two ellipsoidal particles with aspect ratios  for contact angle  in (a) side-by-side, (b) tip-to-tip and (c) tip-to-side orientation calculated using triangulated surfaces. The particles attract each other in side-by-side and tip-to-tip orientation and repel each other in tip-to-side orientation. The side-by-side orientation is energetically most stable. (d-e) Interaction energies  for two identical ellipsoidal particles with aspect ratios (d)  and (e)  and contact angles   =   and . The energies are plotted as function of the distance  between between the centers of the ellipsoids for side-by-side (S-S), tip-to-tip (T-T), and tip-to-side (T-S) orientation. The interaction is attractive in S-S and T-T orientation and repulsive in T-S orientation. Inset: fit of the numerical data using ; the fit parameters are given in the figure. Reprinted with permission from [43]. Copyright (2014) American Chemical Society.

**Figure 26.**
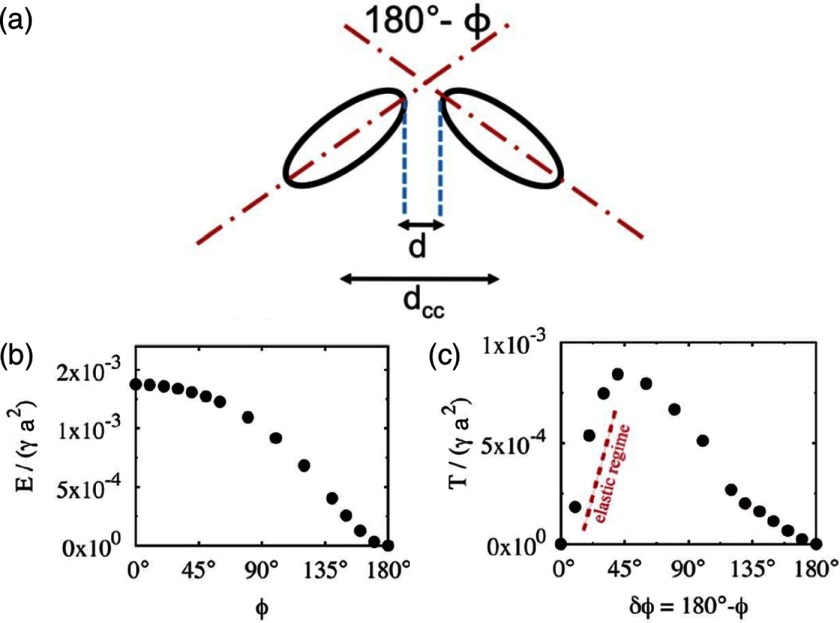

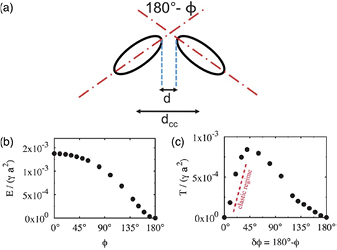

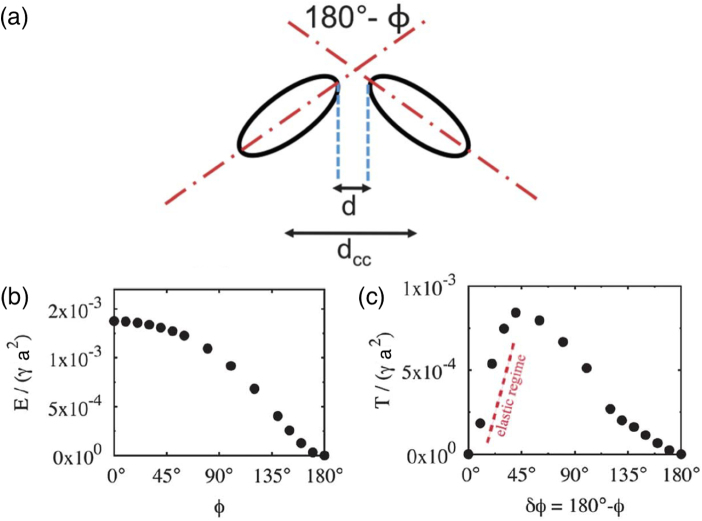
Capillary interaction between two identical ellipsoidal particles. (a) Definition of the parameters. ((b), (c)) Bond-bending energies *E* and torques *T* as function of the deviation from the stable side-by-side orientation for two ellipsoids with  and . A linear-elastic regime for the torques is found for bond angles . Adapted from [76] with permission of The Royal Society of Chemistry.

**Figure 27.**
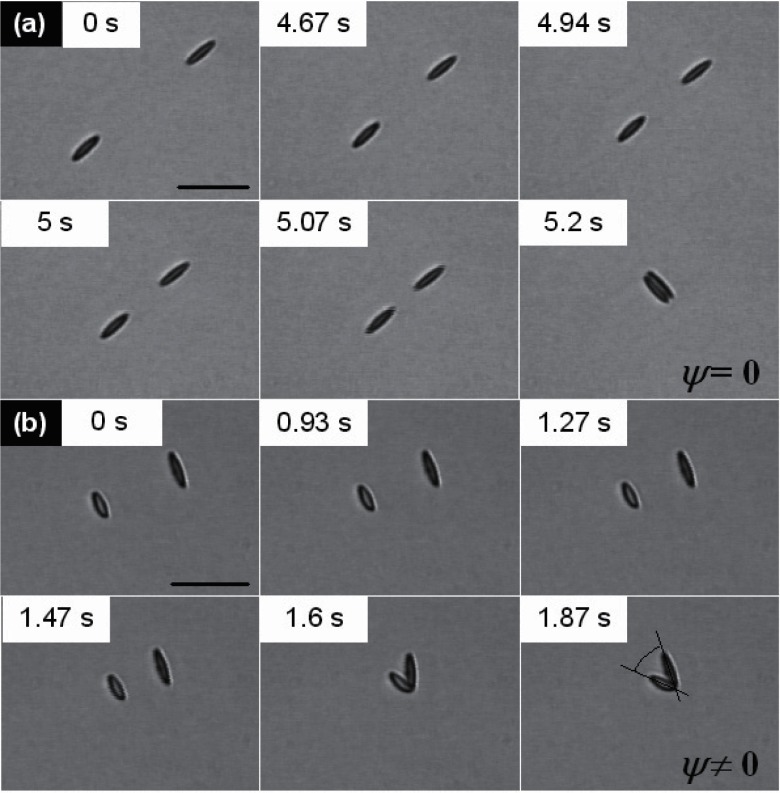

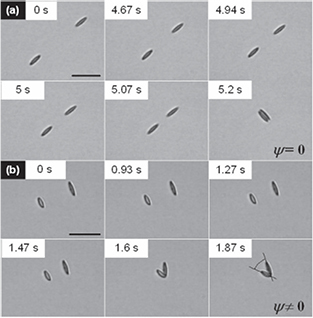

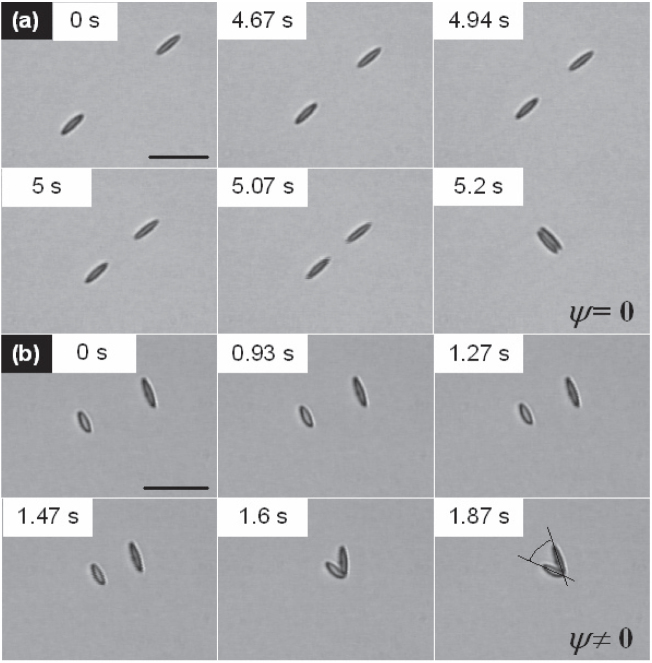
Two ellipsoidal particles approaching side-by-side with (a) equal and (b) dissimilar shapes. Equal particles assemble with their long axes parallel, ellipsoidal particles with different sizes form a capillary arrow. Reprinted with permission from [112]. © EPLA. All rights reserved.

Using triangulated surfaces and energy minimisation, numerical calculations of deformation energies have been applied for various particle shapes, orientations, and contact angles. Figure 25 shows snapshots and interaction potentials for pairs of identical ellipsoidal particles with aspect ratios  and  [43]. The bond energies increase quadratically as function of the bond angle  that quantifies deviations from the side-by-side orientation with . Figure 26 shows the bond-bending energies for two ellipsoids with  and . The capillary torque  on each particle quantifies the resistance to rotation from the equilibrium state. The plot of *T* versus  shows a linear elastic regime with , where  [76]; the torques may reach values up to 10^3^–. A bending modulus for the polymer-like assemblies of particles at the interface can be extracted from many-particle calculations. If two ellipsoids with different aspect ratios interact, the stable assembly is an arrow [112], see figure 27.

**Figure 28.**
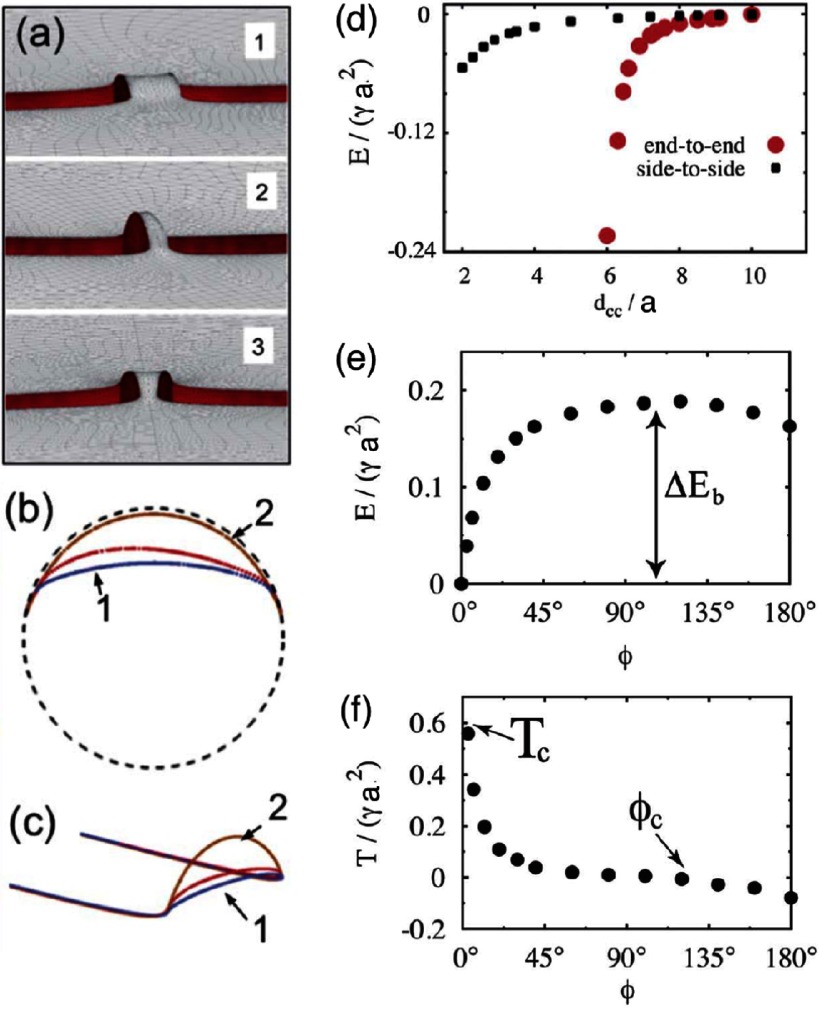

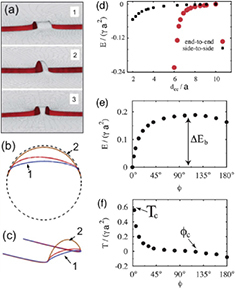

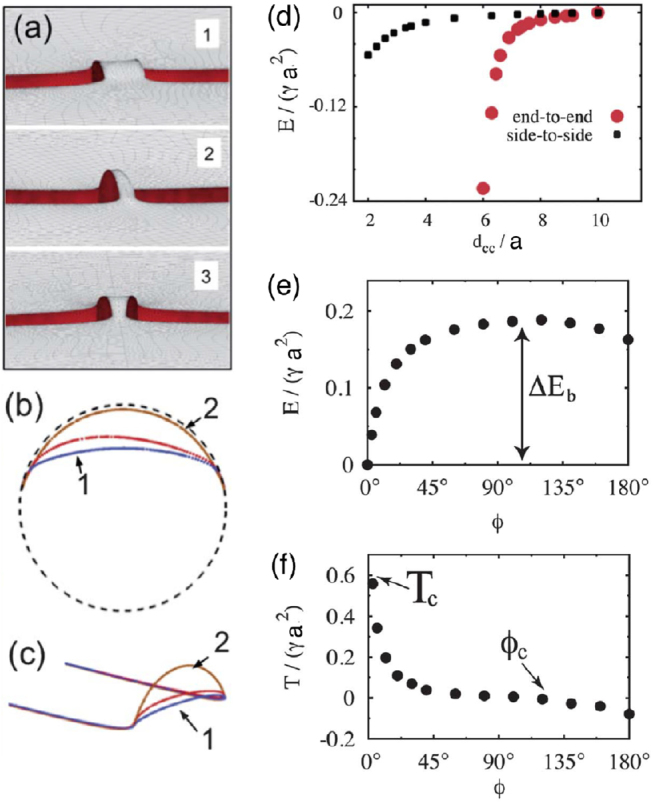
Near-field interaction between two cylindrical particles of aspect ratio  for contact angle . (a) Interface shape for the shortest distance between to cylinders  and  (configuration ‘1’),  and  (configuration ‘2’),  and  (configuration ‘3’). The light (grey) and dark (red) surfaces are the vapor–liquid and liquid-solid interfaces, respectively. Visualization of the solid-vapor interface has been disabled to allow examination of the meniscus between the cylinders. (b) Contact line profiles at the end face of each cylinder. (c) Perspective views of the contact line profiles shown in (b). (d) Capillary energy versus center-to-center separation for ellipsoids. In this figure, the value of the energy for  is taken as reference. (e) Capillary energy versus bond angle for ellipsoids in contact. (f) Capillary torque corresponding to the energy in (e), as a function of the angular deviation from the stable side-to-side configuration. Adapted from [76] with permission of The Royal Society of Chemistry.

Unlike ellipsoidal particles, cylindrical particles preferentially assemble in end-to-end orientation, see figure 28. Because of the flat ends of cylindrical particles, the contact line can adjust its height rather freely if the height of the surrounding interface changes because the contact angle remains unchanged. When two cylindrical particles approach each other, the energy gain for wetting the surfaces overcomes the costs for the deformation of the surrounding interface, thus the interface forms a capillary bridge between the two ends. The bond-bending energies for cylindrical particles around their preferred tip-to-tip state are very high, with restoring torques up to  [76]. Therefore, such chains are usually not distorted. Two cylindrical particles may switch to the metastable side-by-side orientation only for bond angles larger than –, which can be achieved for particulate monolayers under compression or shear stress.

The height of the capillary bridge that forms between two cylindrical particles decreases when the distance between them is increased, compare snapshots for  with  in figure 28; it changes also if the particles are tilted with respect to each other, compare snapshots for  and , and for  and . For cylindrical particles, bond bending is not elastic as for ellipsoidal particles, but shows non-elastic hinging behaviour. Therefore, both bond energies and bond-bending energies are significantly affected by deformations of the surrounding interface and by changes of the wetting of the planar faces of the particles. This importance of wetting energy is a qualitative difference for the capillary interactions between ellipsoidal and between cylindrical particles. The crossover between elastic bond-bending and non-elastic hinging can be further explored using superegg-shaped particles with variable edge curvatures, as discussed in [43].

**Figure 29.**
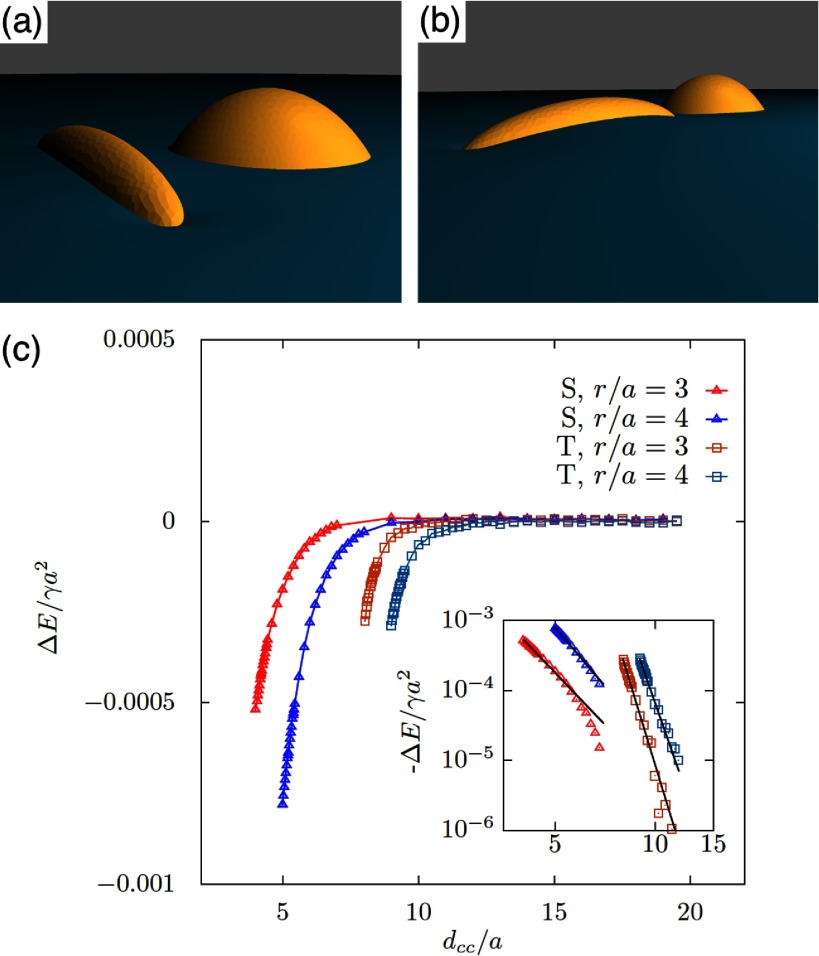

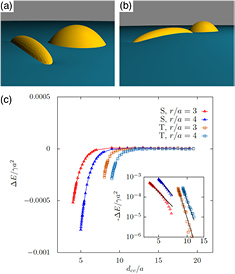

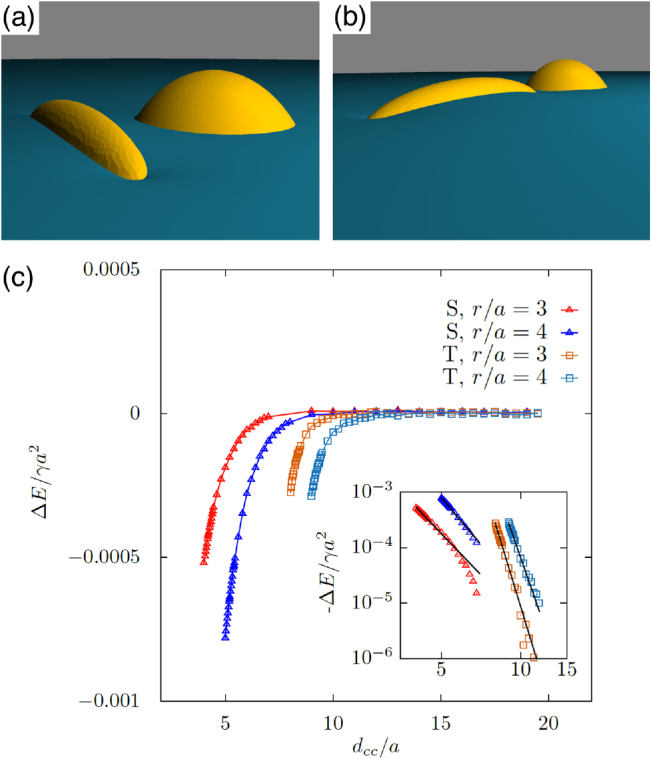
Interaction of a spherical particle with an ellipsoidal particle of aspect ratio . The contact angle  has been used for both particles. The energies are plotted for spherical particles with radii  and  that approach the ellipsoidal particle (a) at the side (b) and at the tip, here shown for a spherical particle with . (c) Interaction energies  as function of  between the centers of mass of the particles. Inset: double-logarithmic plot and the fit of the numerical data to a power-law decay. Reprinted with permission from [43]. Copyright (2014) American Chemical Society.

In general, the presence of one particle changes the contact-line position on the other particle. Figure 7(c) shows the deformation of the initially circular contact line around a spherical particle by a nearby ellipsoidal particle. Figure 29 shows that although the spherical particle at a planar interface by itself does not deform the interface and does not induce interface-mediated attraction, an ellipsoidal and a spherical particle mutually attract each other. The attractive interaction between spherical and ellipsoidal particles is larger for side-on configurations of the spherical particles than for tip-on configurations. In both configurations, however, the attraction between spherical and ellipsoidal particles is significantly weaker than the attraction between two ellipsoidal particles with similar sizes that have equal aspect ratios and surface properties.

### 2.6. Many-particle interactions

Attractive energies for capillary bonds and energy barriers between locally stable configurations are often high, therefore computer simulations show self-assembly in kinetically-trapped dendritic and raft-like structures [114]. Numerical calculations for two-particle, three-particle, and four-particle interactions predict a variety of stable and metastable configurations [77, 115]. Many-particle studies of particles at interfaces can also be extended to polydisperse particle mixtures [116].

**Figure 30.**
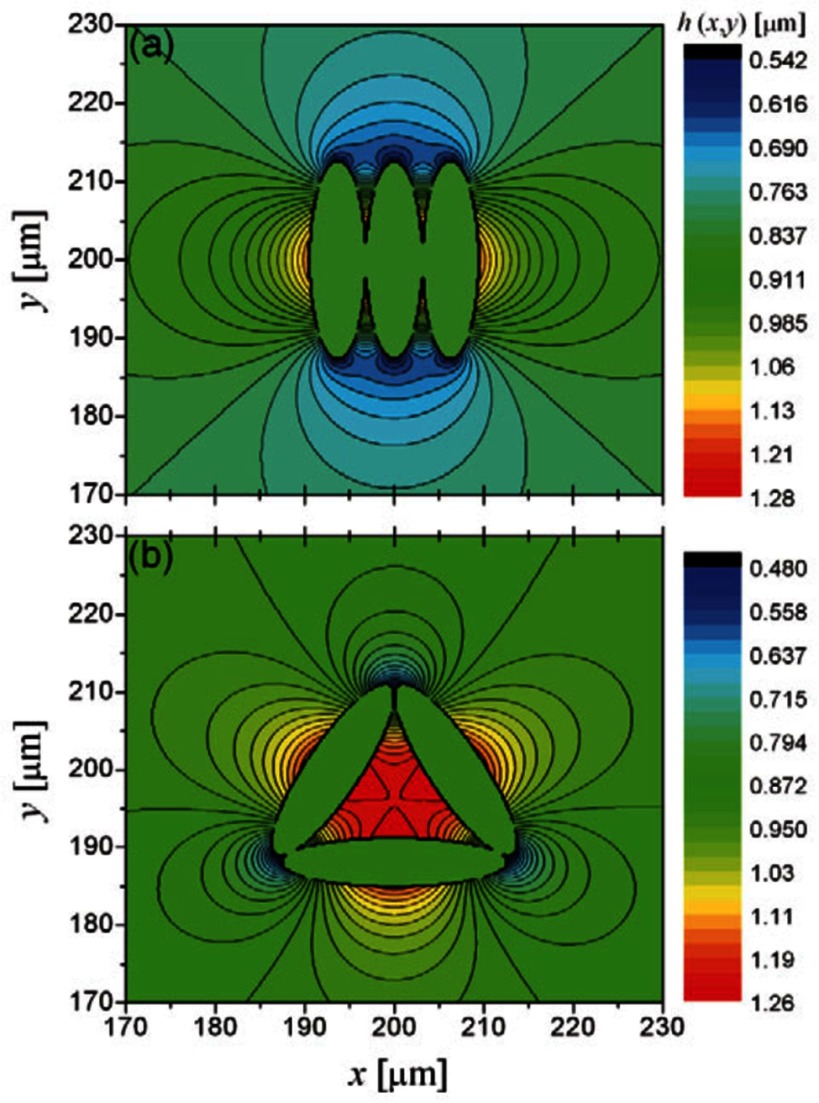

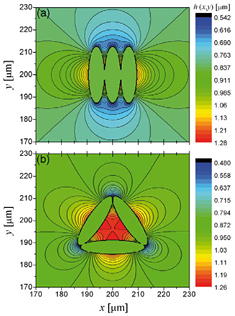

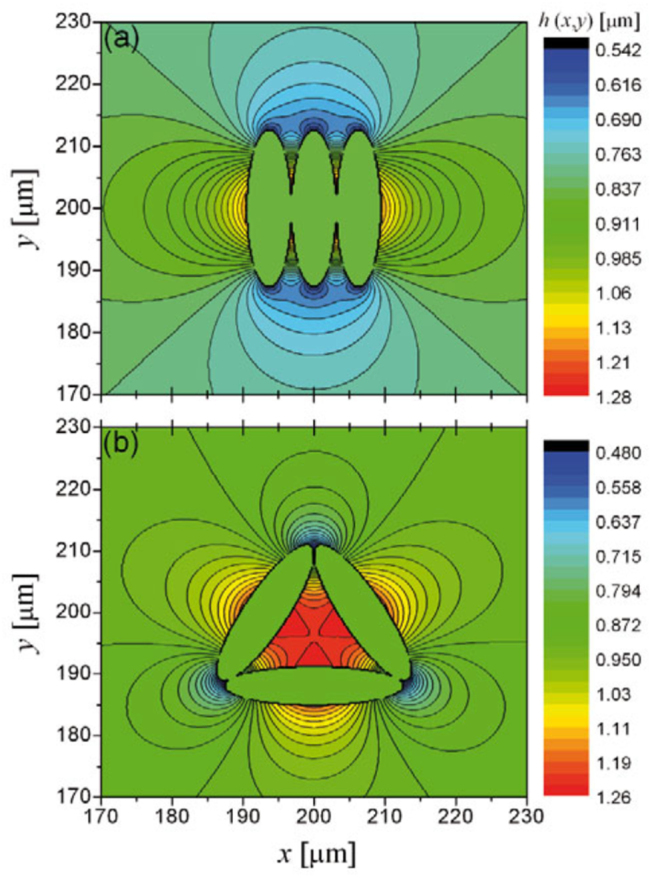
Computed contour plots of  in the case of three contacting equal ellipsoids. The energy of the side-by-side configuration (a) is a bit lower than that of the triangular one (b). The ellipsoidal particles with aspect ratios  have been fabricated by deforming spherical particles with ; , and . Reprinted from [77] with permission. 2011 © EDP Sciences, SIF, Springer-Verlag.

**Figure 31.**
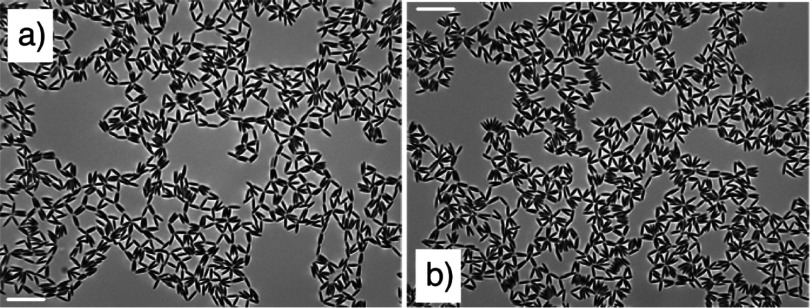

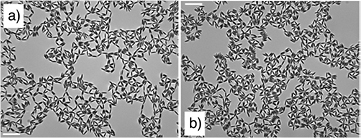

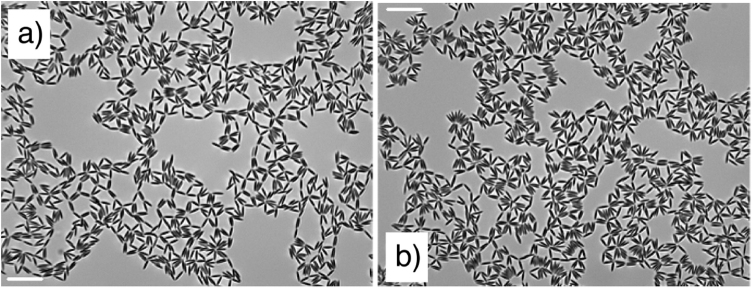
Self-assembly of ellipsoids with low surface charge at higher surface coverage on a water-decane interface. (a) Initial structure. (b) After . The aspect ratio of the ellipsoids is 5.5. The scale bar corresponds to . Reprinted with permission from [113]. Copyright (2009) American Chemical Society.

Figure 30 shows two locally stable configurations of three ellipsoidal particles. The parallel configuration is globally stable with an energy , but the triangular configuration has a similar energy  for , , and ellipsoidal particles with aspect ratio  that have been obtained by deformation of spherical particles with  [77]. For prolate ellipsoidal particles mostly the side-by-side orientation is observed in experiments, hexagonal networks can be stable if an additional repulsive electrostatic repulsion contributes to the particle-particle interaction. Figure 31(a) shows self-assembly of slightly charged micrometer-sized ellipsoidal particles. The structure initially shows many tip-to-tip contacts; after slow relaxation it compactifies slightly and shows less triangular and more chain-like aggregates in side-by-side orientation after  [113], see figure 31(b). The chains of particles in side-by-side orientation can be thought of as ‘colloidal polymers’: worm-like chains or rings with bending elasticity.

**Figure 32.**
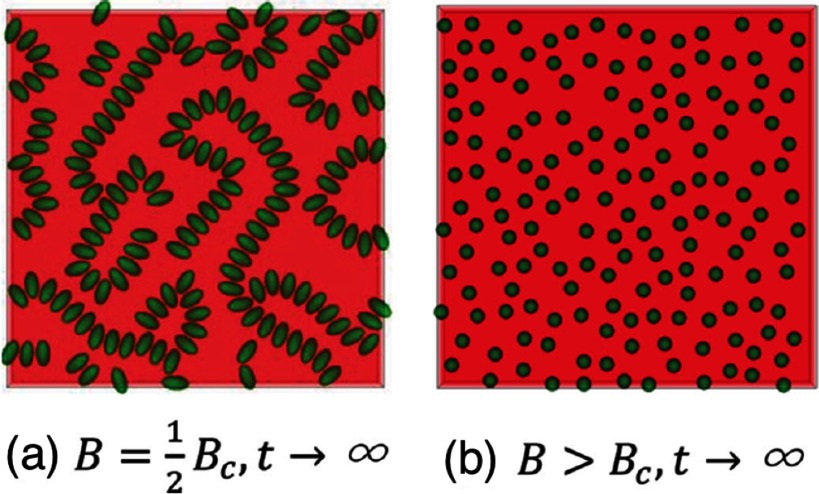

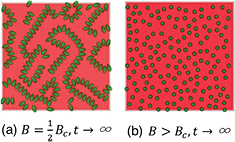

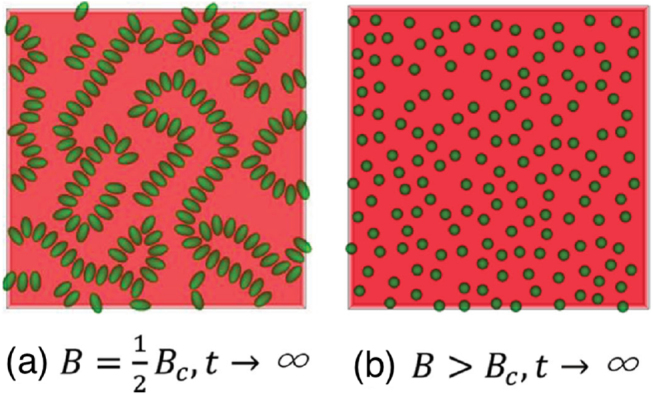
Ellipsoidal magnetic microparticles at interfaces. The neutrally-wetting particles (contact angle ) are initially distributed randomly in their equilibrium orientations with surface fraction . (a) Applying a magnetic field parallel to the interface normal, , causes them to self-assemble due to dipolar capillary interactions. (b) Once the critical dipole field strength *B*_c_ is reached, the particles transition to the vertical state, halting dipolar capillary interactions. The particles randomly redistribute if magnetic dipole-dipole and van der Waals interactions are weak compared with thermal fluctuations. Reprinted with permission from [110]. OA CC BY 3.0.

For non-spherical particles at planar interfaces, particle-particle interactions may affect the orientations of particles and vice versa. For example, cube-like particles at lower interfacial densities assemble on hexagonal and honeycomb lattices in corner-top orientation [97], and at higher interfacial densities on a square lattice in face-top orientation [117]. Figure 32 shows how particle orientations affect interparticle interactions: chains form for prolate ellipsoidal particles that are oriented with their long axes parallel to the interface, while particles disperse homogeneously if they are oriented with their long axes perpendicular to the interface. Particle orientation can be controlled using an external magnetic field [110], see section 2.4.3. A similar tunable (dipolar) interface deformation and therefore tunable capillary interaction can be achieved using magnetic spherical Janus particles [118].

**Figure 33.**
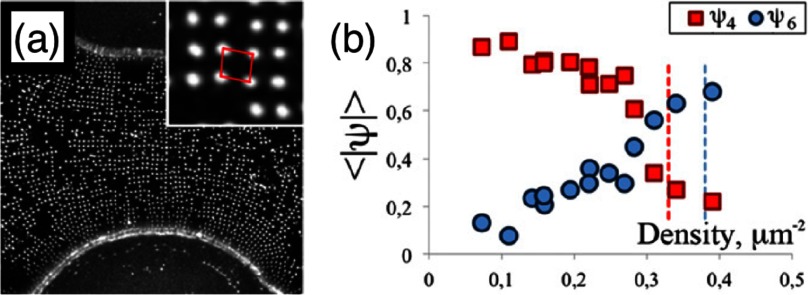

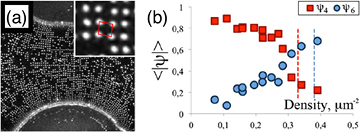

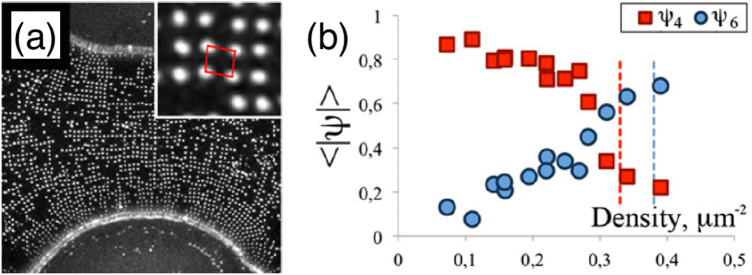
Transition from square to hexagonal packing at high particle densities. (a) Part of a dumbbell-shaped droplet covered with colloidal particles at relatively low density (). The particles organize in a square pattern with a bond-orientational order parameter for four-fold symmertry  and a bond-orientational order parameter for six-fold symmetry . (b) Bond order parameters for four- and sixfold symmetry as a function of particle density for interfaces with deviatoric curvature . The red and blue vertical dashed lines indicate maximum densities for a particle separation of  in a square and a hexagonal lattice, respectively. Reprinted with permission from [61].

Interface curvature alters particle-induced interface deformations and therefore many-particle self-assembly, see section 2.3.2. In order to systematically understand the interaction of many particles at non-planar interfaces, it is important to study model systems with controlled curvatures [61, 118–121]. For example, spherical particles at interfaces with saddle-like shapes induce quadrupolar interface deformations and therefore at low densities assemble on a square lattice [61], see figure 33(a); at high densities, the optimal packing on a hexagonal lattice is observed. The local lattice structure can be investigated using bond-orientational order parameters,

25 where *N*_*j*_ indicates the number of neighbors of a particle. The angle  is the angle between the bond with a neighboring particle *j* and an arbitrary reference axis. Figure 33(b) shows that the lattice order switches continuously from square to hexagonal with increasing particle density.

### 2.7. Particles in ordered fluids

Particles embedded within a nematic liquid crystal (LC) interact with the nematic directors in the vicinity of the colloid and therefore impose boundary conditions on the nematic order parameter at the colloid-LC boundary. This leads to long-ranged anisotropic elastic interactions for spherical and ellipsoidal particles dispersed in nematogenic fluids [122, 123]. Due to varying nature of nematic defects at the colloid-LC boundary, typically elastic multipoles, such as dipoles and quadrupoles, are observed. Large pair interaction strengths render the self-assembled aggregates insensitive to thermal or hydrodynamic fluctuations [124]. Capillary interactions dominate the elastic energies [125]. For microspheres in nematic films, strength and nature of the elastocapillary interactions depend on film thickness and particle size [126]. For small thicknesses of films, giant elastic dipoles occur due to interface distortions. Defects appear around particles, elastic dipoles and nematic elasticity counterbalance the strongly attractive capillary interactions and stabilize particles pairs at finite distance.

**Figure 34.**
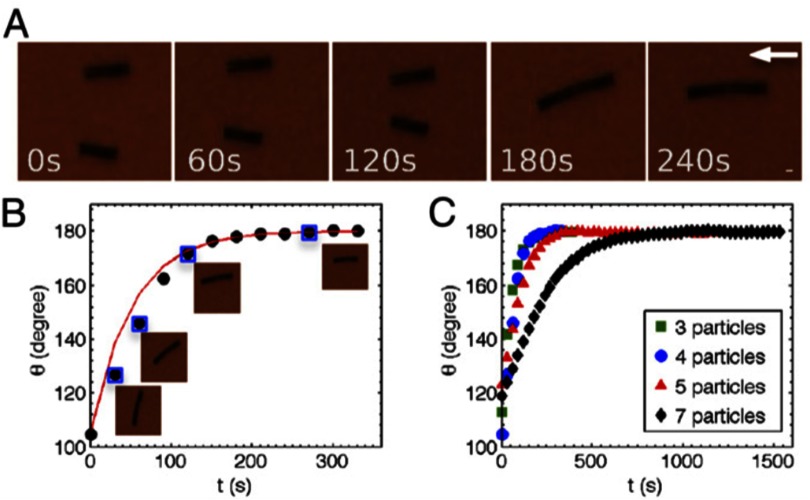

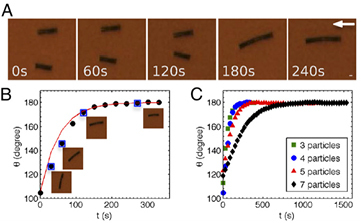

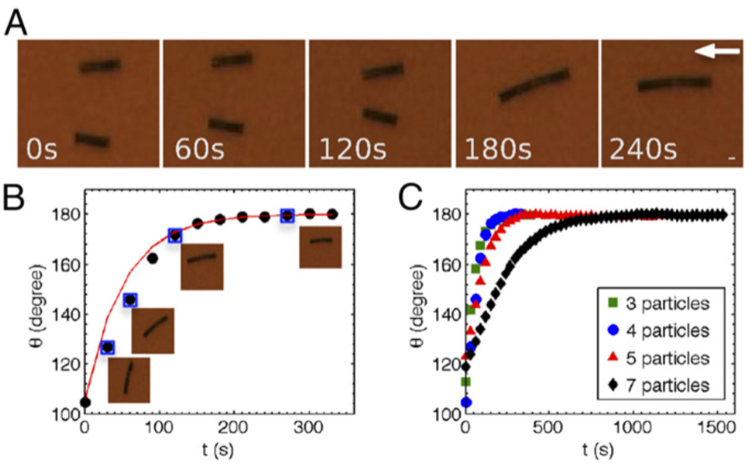
Particles at interfaces of ordered fluids. (a) Upon forming a rigid chain, the aggregate rotates such that the chain axis aligns parallel to the director field. Arrow indicates the direction of LC alignment. The scale bar represented by the arrow corresponds to . (b) Elastic rotation rate of a chain comprising four particles. (c) Rotations observed on the interface for chains of three, four, five, and seven particles in length. Reprinted with permission from [125].

Figure 34 shows how cylindrical nanoparticles first assemble at a nematic interface in their energetically favourable tip-to-tip orientation and then align with the nematic director [125]. The weak elastic interactions are able to manifest themselves at flat nematic interfaces, but under curvature gradients again capillarity dominates [125]. Particle anisotropy shall also play a significant role for defect formation at colloid-LC boundaries and shall thus also control nature and strength of pair-particle interactions. Future experiments and theoretical investigations to elucidate these elusive interplay between capillarity and elasticity are warranted.

### 2.8. Applications

Applications for (engineered) particles at interfaces range from emulsion stabilisation and colloidosomes to suppression of the coffee-ring effect and froth floatation. In addition to capillary forces, also for example Marangoni flows, surfactants, electric charges, or gravity have to be taken into account.

#### 2.8.1. Pickering emulsions.

A classical application is the stabilisation of emulsions. Here, the interactions of particles at interfaces on the mesoscale can be expected to determine macroscopic rheologial properties. In general, the strong adsorption of particles to interfaces leads to thermodynamically stable droplets of oil (water) suspended in water (oil) [1, 3, 127–129]. At low particle concentrations, the mechanism of limited coalescence generates narrow and reproducible droplet-size distributions [117]. Phase inversion of droplet emulsions is obtained by increasing the volume fraction of oil (water). For mixtures for small hydrophilic and hydrophobic particles also sponge-like, bicontinuous phases and non-spherical colloidosomes have been observed [4, 130]. Particles with anisotropic surface functionalization, such as Janus particles, can be used as amphiphilic colloidal surfactants [131, 132]. Particle-stabilised emulsions are used for example in food and cosmetic industries [8–11].

#### 2.8.2. Colloidosomes.

Colloidosomes are solid capsules engineered with controlled permeability and mechanical strength that can have sizes from sub-micrometers to millimeters [133]. The capsules are prepared by self-assembly of colloidal particles to emulsion droplets that are then locked together for example using sintering or electrostatic binding of an oppositely-charged polyelectrolyte. This ensures that the shells remain intact when they are transferred to a different fluid. With the help of centrifugation, the particles are typically transferred into a solvent that is identical to the internal phase. Colloidosomes can be used for encapsulation of drugs, proteins, vitamins, flavors, gas bubbles, and even living cells.

#### 2.8.3. Suppression of the coffee-ring effect.

Particles that are homogeneously dispersed over an entire drop often form ring-like deposits when the drop evaporates, so-called ‘coffee rings’ [134]. Addition of ellipsoidal particles with aspect ratio , obtained by deforming spherical particles with , to droplets that contain the spherical particles has been found to lead to more homogeneous deposition [135]. For droplets that contain ellipsoidal particles only, a strong suppression of the coffee ring effect is already observed for very moderate aspect ratios . Numerical calculations predict capillary attraction between spherical and ellipsoidal particles [43]; the uniform deposition may therefore be caused by capillarity-induced cluster formation. Applications for that a more uniform deposition of the material instead of ‘coffee rings’ may be desired are inkjet printing, fabrication of micro- and nanostructures, and coating.

#### 2.8.4. Froth floatation.

Froth floatation is used to separate minerals from gangue. The separation efficiency depends on both the particle-bubble attachment and the froth stability [80, 136]. While the particle-bubble attachment can be adjusted using collector chemicals that modify the particle’s surface properties, the film stability is affected by particle shape. One complication that decreases froth stability can be particles with sharp edges [79]. Therefore, a systematic understanding of the interaction of non-spherical particles with thin films and potentially also of immersion forces may help to improve froth floatation [80, 98, 102, 136].

## 3. Particles at biological interfaces

**Figure 35.**
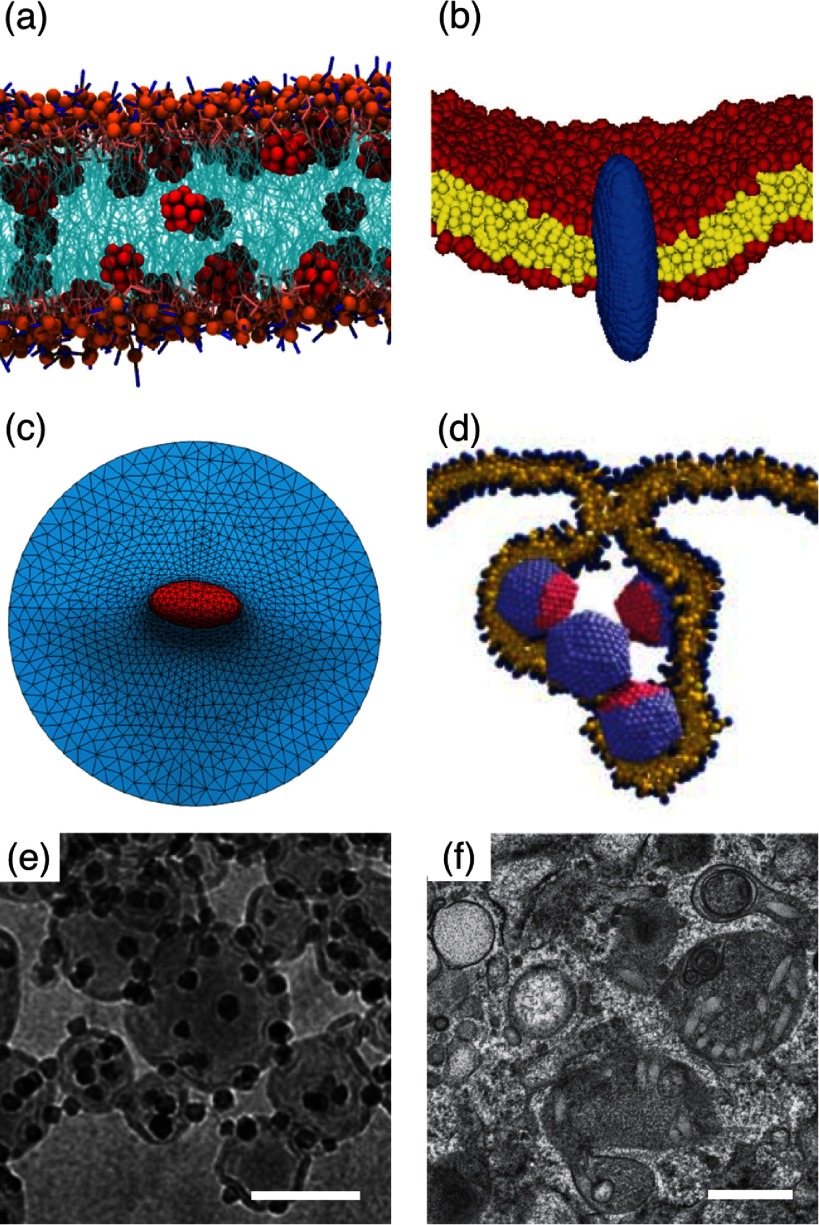

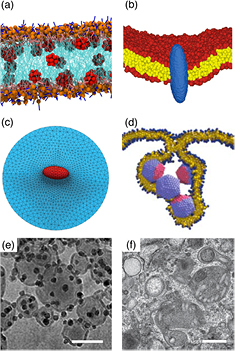

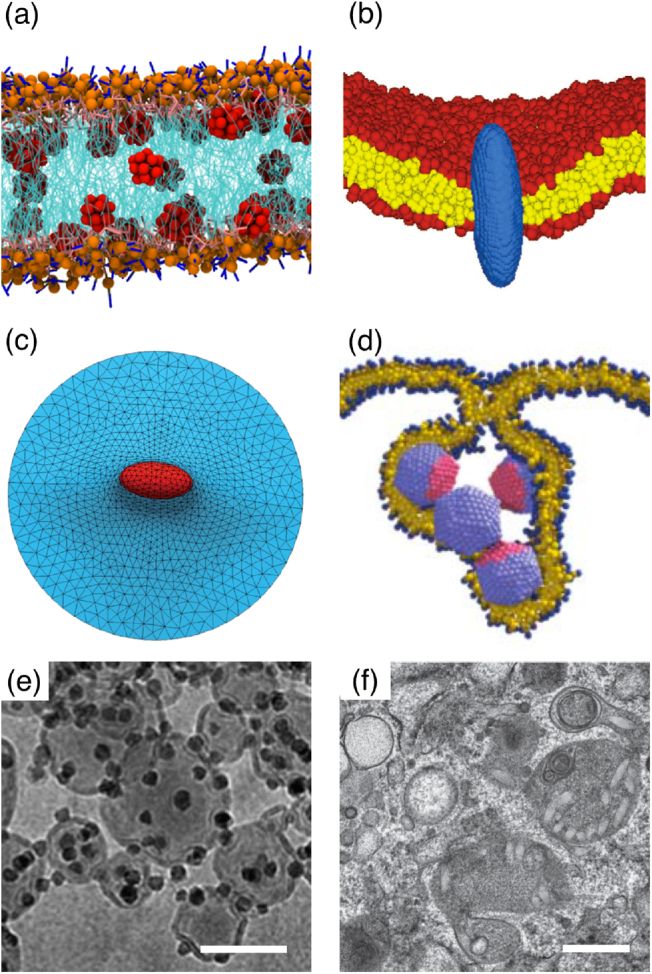
Interaction of nanoparticles with membranes. (a) Incorporation. Fullerenes in a POPC membrane. Adapted with permission from [137]. Copyrighted by the American Physical Society. (b) Penetration. An ellipsoidal nanoparticle passes through a lipid bilayer. Adapted by permission from Macmillan Publishers Ltd: *Nat. Nanotechnol*. [138], copyright © 2010. (c) Wrapping. An ellipsoidal nanoparticle gets wrapped by a lipid bilayer membrane. Reproduced from [139] with permission from the Royal Society of Chemistry. (d) Cooperative wrapping. Capsids bud cooperatively. Adapted by permission from Macmillan Publishers Ltd: Nature, [140], copyright © 2007. (e) Cryo-TEM micrographs of nanoparticles interacting with vesicles. The scale bar corresponds to . Adapted from [51] with permission of The Royal Society of Chemistry. (f) TEM micrographs of nanoparticles internalized in human mesenchymal stem cells. The scale bar corresponds to . Adapted with permission from [141]. Copyright © 2012 Wiley-VCH Verlag GmbH & Co. KGaA, Weinheim.

Particles adsorb at biological interfaces, often called membranes, because of an adhesion strength *w* between the particles and the membranes. In addition to the adhesion strength, the minimal ingredients required to study spherical particles are the bending ridigity *κ* and the tension *γ* of the membranes, and the radius *a* of the particles. For small particles, molecular interactions are important. Coarse-grained or even atomistic computer simulations are used to investigate theoretically translocation through and incorporation within membranes [142–145]. Experimentally, the interaction of small particles with membranes can be investigated using a combination of microfluidic devices, fluorescence microscopy, and electrophysiological measurements [146], by scattering techniques [147], and by quartz crystal microbalance and AFM [148]. For larger particles with radii , wrapping is the dominant mechanism of the interaction between particles and membranes, as will be discussed in detail in this section. Particles that interact with membranes can be engineered and are found in biological systems with a wide variety of sizes, shapes, and surface functionalizations, see figure 35.

Comparing the different energetic contributions, large spherical nanoparticle adhesion and wrapping is determined by two characteristic crossover radii,

26

The radius  that compares the bending energy with the membrane tension characterizes the crossover between the bending-dominated regime for particles with  and the tension-dominated regime for larger particles. While for nanoparticles bending energy is thus the main player, for micrometer-sized particles tension plays the dominant role. Within the bending-dominated regime, small particles with  remain unwrapped, while larger particles get wrapped, determined by the subtle balance of bending and adhesion energy [139, 149–151]. For given particle radius, the threshold adhesion strength  therefore marks the transition between the unwrapped and the complete-wrapped regime.

Adhesion may be mediated by van der Waals interaction [152], hydrophobic interaction [152], electrostatic interaction [153], and specific adhesion via receptor-ligand bonds [154–160]. In biological media, nanoparticles can be surrounded with a corona of proteins that effectively changes their surface properties [161–166]. Throughout this section, we assume that the adhesion can be modeled by a continuous and mostly homogeneous adhesion strength, which is appropriate for many systems, but not always sufficient for systems dominated by receptor-ligand bonds and electrostatic interactions. Typical values for the bending rigidity are , for the membrane tension  [149, 167], and for the adhesion strength  [42]. Instead of the absolute values for energy, tension and adhesion strength, it is often convenient to use dimensionless, reduced values, e.g. , , and .

An overview of both single-particle and many-particle systems at membranes is provided in this section. In sections 3.1–3.4 we focus on various aspects of the interaction of single particles with membranes, while in sections 3.5 and 3.6 we discuss two-particle and many-particle interactions, respectively. Section 3.7 focuses on particles at membranes with spontaneous curvature. We finally discuss applications in section 3.8.

### 3.1. Spherical particle-induced membrane deformation

**Figure 36.**
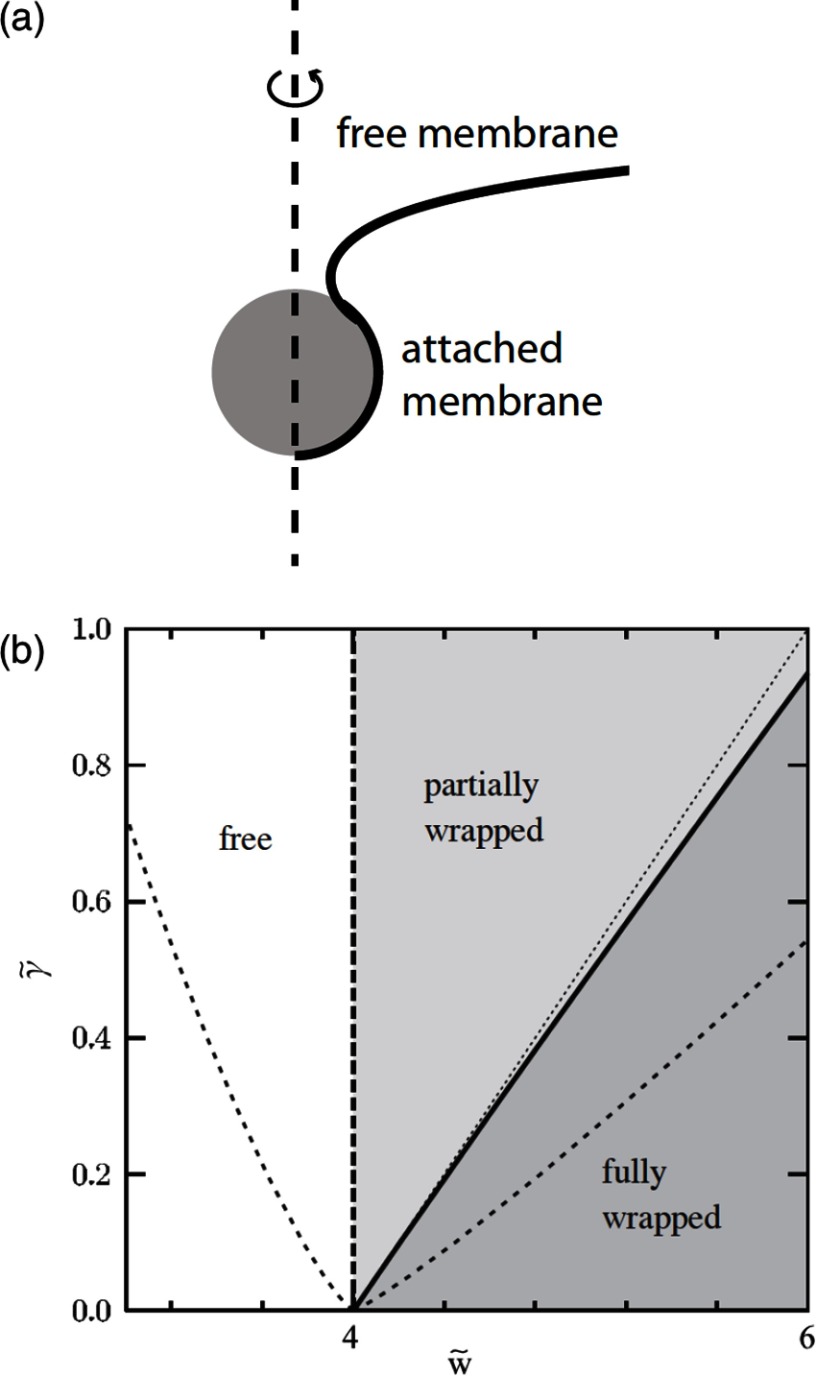

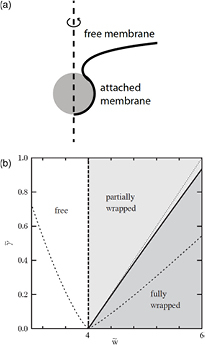

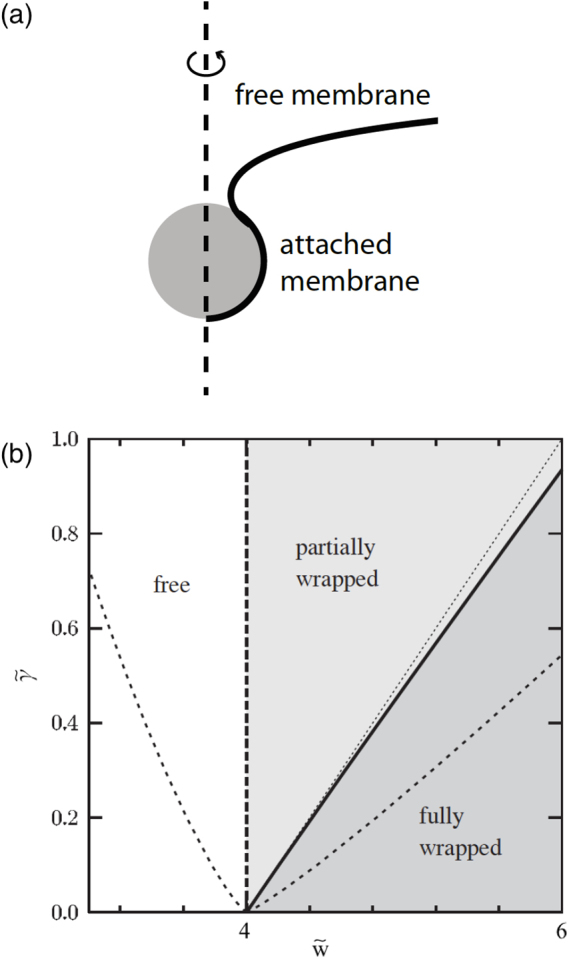
Wrapping of a spherical nanoparticle for various reduced adhesion strengths  and membrane tensions . (a) The membrane deforms in a cylindrically symmetric way around the symmetry axis and the shape can be described by a radial deformation profile. For an infinitely large tensionless membrane, the shape of the free membrane is catenoidal. (b) The wrapping phase diagram shows that the nanoparticles are unwrapped below a critical adhesion strength , and completely enveloped for adhesion strengths beyond the thick solid line. The thin dotted line is the envelopment transition calculated neglecting the deformation energy of the free membrane. The thin dashed lines are the spinodals for spontaneous unwrapping from the complete-wrapped to the free/non-wrapped state and for spontaneous envelopment from the partially wrapped to the fully wrapped state. Adapted with permission from [168]. © EPLA. All rights reserved.

For a spherical particle and an infinitely large tensionless membrane, the membrane surrounding the particle assumes a catenoidal shape, see figure 36(a). This shape is found around all cylindrically-symmetric, ‘conical’ membrane inclusions, which includes curved proteins [140, 169, 170] and attached polymers [171–174]. A catenoid is a minimal surface with vanishing mean curvature at every point and therefore with vanishing bending energy. The deformation energy cost for the membrane adhered to the particle and the adhesion energy gain for contact between particle and membrane can therefore be directly compared with each other in order to predict whether a particle gets wrapped or not. The wrapping transition is fully characterized using the critical particle radius  for wrapping in equation (26). However, in general the deformation energy of the membrane surrounding a particle will contribute to the total deformation energy of the membrane.

A convenient way to characterize wrapping states of particles for various parameters are wrapping diagrams based on energy minimisation, analogous to phase diagrams in thermodynamics [139, 149, 175], see figure 36(b). Spherical particles remain unwrapped below a threshold adhesion strength and get partial-wrapped or complete-wrapped for higher adhesion strengths. Particles at membranes with finite tension can be found in stable partial-wrapped states for adhesion strengths just above those for the binding transitions, see figure 36(b). The stable partial-wrapped states are separated by continuous transitions from the unwrapped states and by discontinuous transitions from the complete-wrapped states. The latter transitions with energy barriers are associated with two spinodals that indicate those values for the adhesion strength beyond which particles transition spontaneously from partial-wrapped to complete-wrapped states and below which particles transition spontaneously from complete-wrapped to unwrapped states.

### 3.2. Membrane deformation by non-spherical particles

Membrane deformations induced by non-spherical particles depend on both particle shape, discussed in this section, and particle orientation, discussed in section 3.4. In general, inhomogeneous surface curvature of particles corresponds to energy barriers for wrapping [139, 176]. For non-spherical particles, membrane tension is therefore not required to stabilize partial-wrapped states; such particles are ideal membrane markers for imaging. Thus not only size and aspect ratio, but also local particle surface curvature matters for wrapping of non-spherical particles [176].

**Figure 37.**
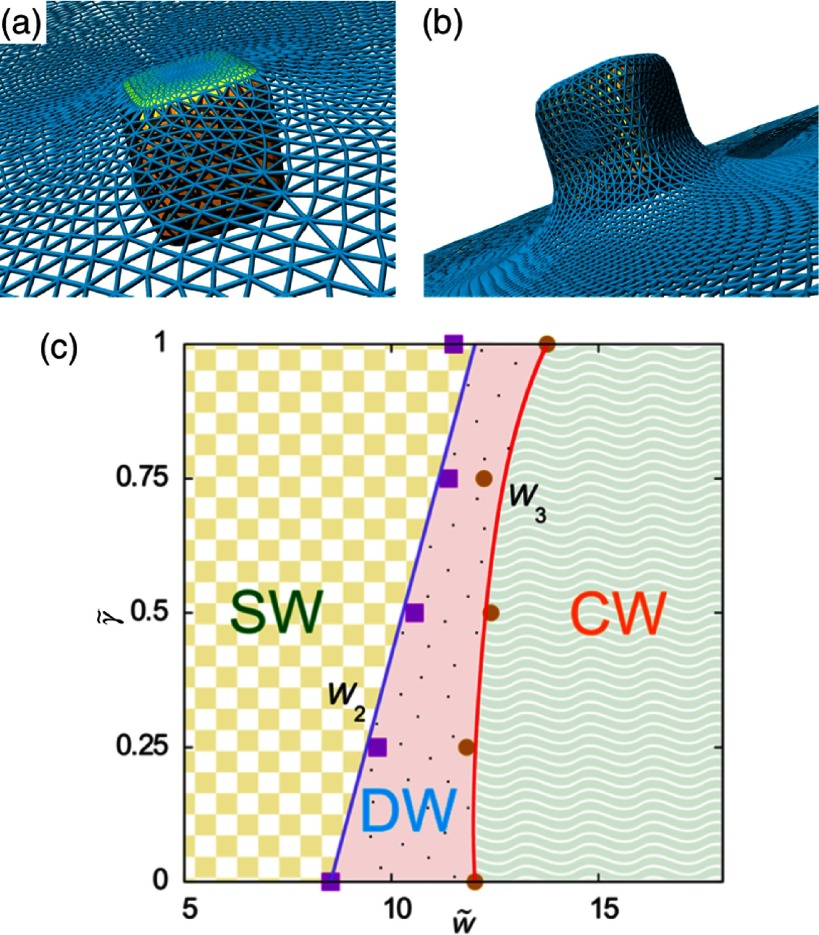

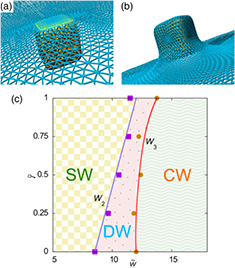

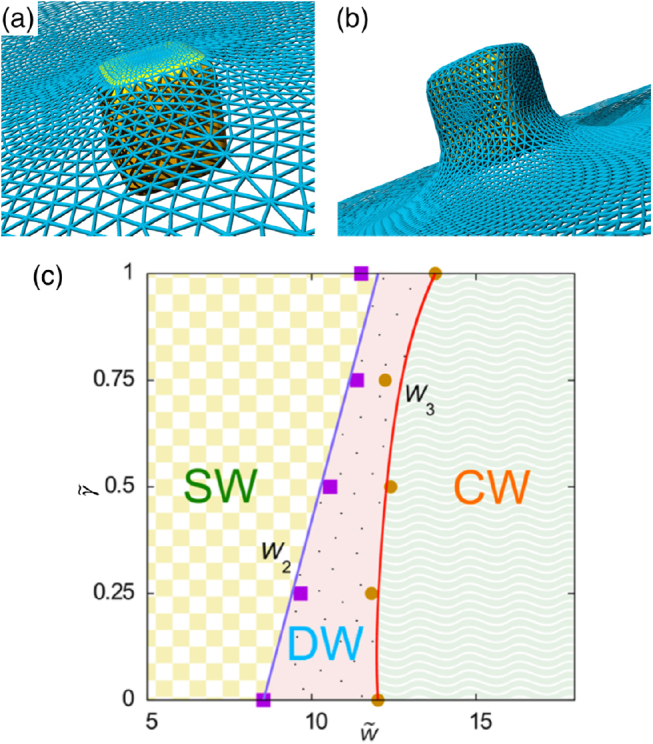
Wrapping of cube-like nanoparticles. (a,b) Membrane deformation for wrapping of Hauser’s cube. The network of edges and triangles describes the membrane shape and is used for the numerical calculations of the curvature energy. Membrane conformations are shown at fixed membrane tension  for two corresponding states at the *W*_2_ phase boundary: (a) a shallow-wrapped state with approximately  of particle wrapped, and (b) a deep-wrapped state with approximately  of the particle wrapped. (c) Phase diagram for wrapping of Hauser’s cube for reduced membrane tension  and reduced adhesion strength , where *A* is the particle surface area. We find a shallow-wrapped (SW), a deep-wrapped (DW), and a complete-wrapped (CW) state, separated by two discontinuous wrapping transitions, labeled as *W*_2_ and *W*_3_. Adapted with permission from [176]. Copyright (2014) American Chemical Society.

The importance of local particle surface curvature can be demonstrated well for Hauser’s cube-shaped particles. The phase diagram in figure 37 shows that no membrane deformation energy costs arise if cube-like particles attach with a flat face to a membrane. The particles therefore adhere already for very small adhesion strengths and are found in shallow-wrapped states. The first energy barriers are encountered between shallow-wrapped and deep-wrapped states when the membrane has to bend around the highly-curved edges to increase wrapping from one face to five faces. The energy barriers between deep-wrapped and complete-wrapped states correspond to the last four edges to be wrapped. Both energy barriers shift to higher adhesion strengths for higher values of the membrane tension.

### 3.3. Field-induced membrane deformation

We discuss here particles within the hydrophobic tail region of lipid bilayers in part 1 and particles at curved membranes in part 2.

#### 3.3.1. Particles within membranes.

**Figure 38.**
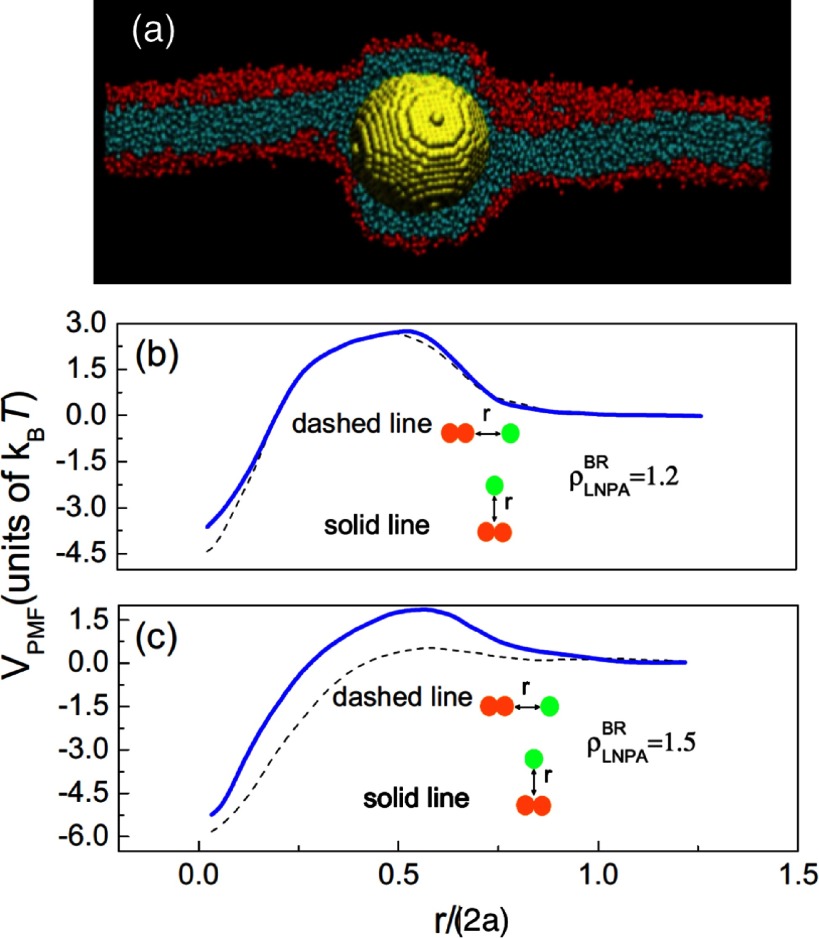

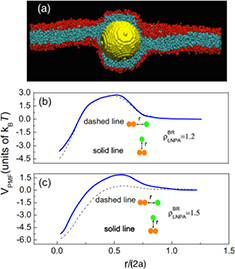

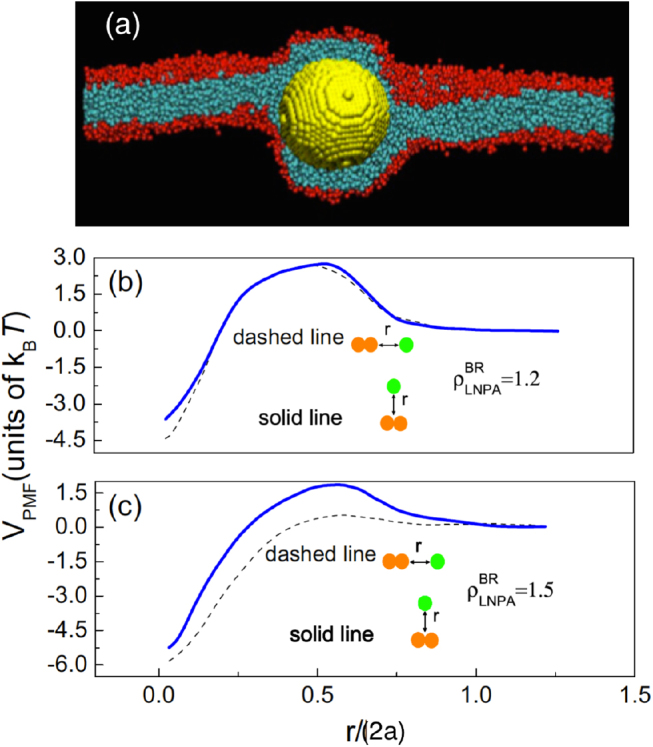
Hydrophobic nanoparticles interacting with membranes. (a) Simulation snapshot of a single nanoparticle radius  incorporated in a lipid membrane for lipid area density . ((b), (c)) Calculated potential of mean force *V*_PMF_ profiles as a third nanoparticle approaches a fixed nanoparticle cluster formed by two identical nanoparticles with radius  at (b) high membrane tension (lipid area density ) and at (c) low membrane tension (lipid area density ). Adapted with permission from [145]. Copyright (2014) the American Physical Society.

Particles with sizes of few nanometers may incorporate themselves in membranes and locally distort both lipid order and membrane thickness [142, 145, 177], see figure 38(a). While lipid order can only be investigated on the molecular scale, thickness variations can also be investigated using continuum models. Here the headgroup positions of the monolayers can be modeled by mathematical surfaces with bending rigidity and monolayer tension, and a confinement potential which maintains the lipid bilayer thickness.

Analogous to integral membrane proteins with hydrophobic mismatch [178–181], particles that locally induce membrane-thickness deformations experience short-ranged deformation-mediated interactions. While the interactions may be repulsive at long distances as predicted by the continuum models, both continuum models and molecular dynamics simulations predict short-ranged attractive interactions and therefore clustering of nanoparticles within membranes. For high membrane tensions, the energy barrier for a third particle approaching a pair of particles is considerably decreased, such that particle aggregation may occur spontaneously [145], see figures 38(b) and (c).

#### 3.3.2. Curved membranes.

**Figure 39.**
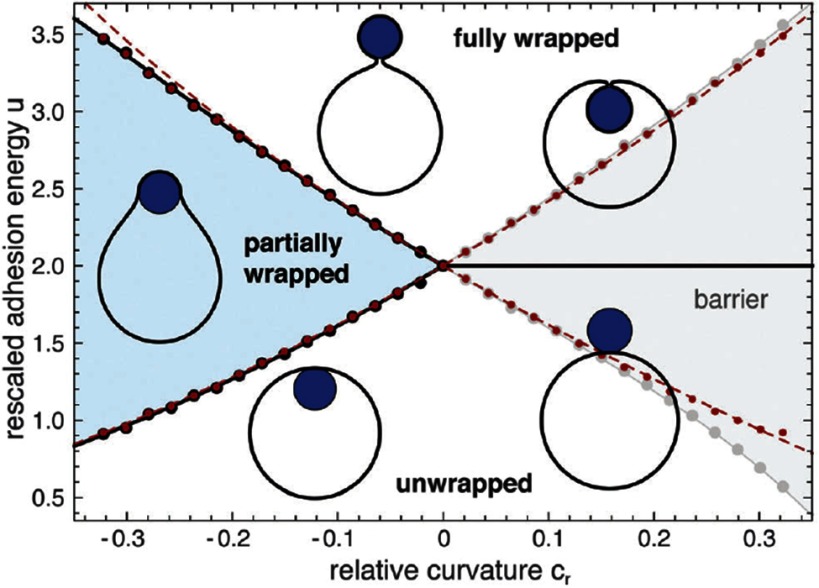

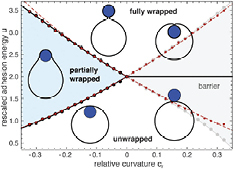

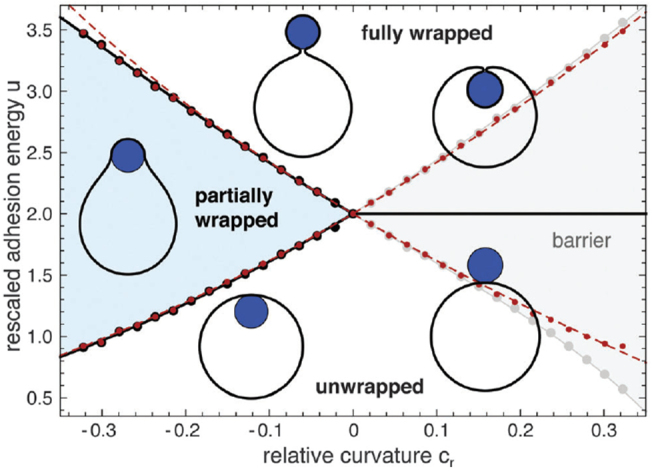
Morphology diagram of stable states of a particle adhering to a vesicle. The wrapping state is plotted for various values of rescaled adhesion energies  and relative curvatures *c*_r_ of the vesicle membrane and the particle surface. The three black lines divide the diagram into three regions in which the particle is either partially wrapped, unwrapped, or fully wrapped. In the grey shaded region, the transitions between the unwrapped state and the wrapped state of a particle outside the vesicle require the crossing of an energy barrier. The red dashed lines are analytical instability lines derived from the stability relations in [42]. Reproduced from [183].—Published by The Royal Society of Chemistry. OA CC BY 3.0.

Not only particle shapes, also membrane curvature prior to wrapping has to be taken into account for the interaction of particles with membranes. This can be demonstrated by calculating entry of particles into and exit of particles out of vesicles with different curvatures, see figure 39. The vesicles are assumed to freely adjust volume but keep a fixed membrane area. Vesicles without particles thus adopt an overall spherical shape that is then locally distorted by binding a nanoparticle. The adhesion strength *w* and the relative curvature of particle and vesicle , for vesicles with radius *R*_v_, are the relevant parameters to characterize this system. They are a subset of the full parameter space, determined in addition by membrane spontaneous curvatures and by osmotic pressures [183].

For particles that enter vesicles from the outside, , wrapping is hindered by an energy barrier. For particles that exit vesicles from the inside, , upon increasing the adhesion strength the particles continuously transit from the free state via stable partial-wrapped states with increasing wrapping fractions to the complete-wrapped state. Partial-wrapped particles can be used as probes for the local membrane curvature [184]. A direct and barrierless transition between the free and the complete-wrapped state is only present for particles at infinitely large planar membranes. With decreasing absolute relative curvature *c*_r_, both the energy barriers for particle entry as well as the stability of partially-wrapped states for particle exit decrease. The adhesion strengths for the wrapping transitions can be well approximated by the instability relations [42, 183]  and , as indicated in figure 39.

### 3.4. Non-spherical shapes, surface heterogeneities, and particle orientations

For non-spherical particles, the orientation of particles at membranes—in addition to particle shapes, sizes, and surface properties—has to be taken into account to determine wrapping states and membrane deformations. Elongated particles can be oriented in rocket and in submarine orientation, with their long axes perpendicular and parallel to the membrane, respectively. Janus particles preferably bind with their most adhesive side to the membrane. Changing the orientation of particles in magnetic fields can be used to probe the elastic properties of membranes and cells.

#### 3.4.1. Elongated particles.

**Figure 40.**
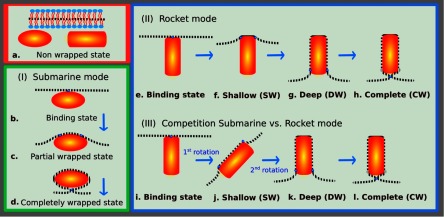

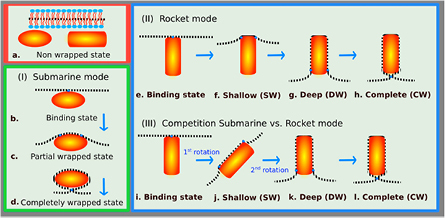

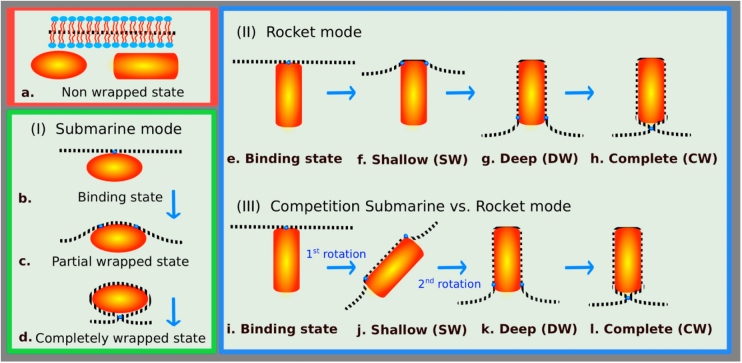
Modes of entry for nanoparticle uptake by membrane wrapping: (I) submarine mode with the long axis of the particles oriented parallel to the membrane, (II) rocket mode with the long axis oriented perpendicular to the membrane, and (III) competition between submarine and rocket mode as observed for rod-like particles with high aspect ratios. The complete-wrapped particle is connected by an infinitely small catenoidal neck to the membrane; the particle orientation in this state is irrelevant. Reprinted with permission from [176]. Copyright (2014) American Chemical Society.

The orientations of elongated particles at membranes crucially depend on particle shapes, membrane elastic properties, and membrane-particle interactions, see figure 40. Table 4 summarizes wrapping states and transitions for various particles shapes and membrane elastic parameters based on the results of [139, 149, 176, 185].

**Table 4.** Shape dependence of particle wrapping, based on the results of [139, 149, 176, 185]. The membrane can be characterized by bending rigidity only, ‘*κ*’, or by bending rigidity and membrane tension, ‘*κ* and *γ*’; the binding transition can occur at finite or vanishing adhesion strength *w*; the particle can be in submarine or rocket orientation; transitions can be continuous (cont.) or discontinuous (discont.) and may involve reorientation (reorient.). The binding transition for ellipsoids is independent of the membrane tension and is given in [139, 185].

Particle shape	Membrane	Binding transition	Shallow-wrapped state	Deep-wrapping transition	Deep-wrapped state	Envelopment transition
Spherical	*κ*	cont., for	—	—	—	binding
Spherical	*κ* and *γ*	cont., for	yes	—	—	discont.
Ellipsoidal	*κ*, *κ* and *γ*	cont., indep. of *γ*	yes, submarine	discont., reorient.	yes, rocket	cont.
Ellipsoidal^a^	*κ*, *κ* and *γ*	cont., indep. of *γ*	yes, submarine	—	—	discont.
Cube-like	*κ*, *κ* and *γ*	at vanishing *w*	yes	discont.	yes	discont.
Sphero-cylinder	*κ*, *κ* and *γ*	at vanishing *w*, rocket	yes, submarine	discont., reorient.	yes, rocket	discont.
Rod-like	*κ*, *κ* and *γ*	at vanishing *w*, rocket	yes, submarine	discont., reorient.	yes, rocket	discont.
Rod-like^a^	*κ*, *κ* and *γ*	at vanishing *w*, rocket	yes, submarine	—	—	discont.
Rod-like^b^	*κ*, *κ* and *γ*	at vanishing *w*, rocket	yes, rocket	discont.	yes, rocket	discont.

a Fast wrapping at high adhesion strength, such that a bound ellipsoid cannot reorient to rocket orientation.

b Rocket mode for supereggs with blunt tips and small aspect ratio (e.g.  and ).

Reprinted with permission from [176]. Copyright (2014) American Chemical Society.

For prolate ellipsoidal particles, the particles bind to membranes with their points of lowest curvature at the sides of the particles. In these shallow-wrapped states, they are in the so-called ‘submarine orientation’. For fast wrapping, if reorientation does not occur, the particles remain in submarine orientation until they reach the complete-wrapped state [139]. For slow wrapping, the particles are able to reorient to their minimal-energy states at every time, they transit from submarine orientation to the so-called ‘rocket orientation’ at deep wrapping. The rocket state is energetically preferable at deep wrapping, because only one of the pointed tips needs to be wrapped. From a stable deep-wrapped state, ellipsoidal particles then continuously transition to the complete-wrapped state [176].

For rod-like particles, energy minimisation predicts that the particles readily bind with their blunt tips in rocket orientation at very small adhesion strengths. If the edges of the particles are sharp and their aspect ratios are small, the particles remain in rocket orientation until they reach complete wrapping; the transitions between shallow-wrapped and deep-wrapped, as well as between deep-wrapped and complete-wrapped states are discontinuous. For high aspect ratios or round edges of the particles, rod-like particles bind with their blunt tips in rocket orientation only with a very small fraction of their surface area. They then rotate to submarine orientation in the shallow-wrapped state, and back to rocket orientation in the deep-wrapped state [176, 186], see figure 40. In particular, the theoretical prediction for the rotation from submarine orientation to rocket orientation is in agreement with experimental observations for budding of filamentous viruses [28, 29].

The dynamics of wrapping of spherical nanoparticles is determined by the typical time scales for the relevant processes, such as membrane deformation [170], receptor or protein diffusion that may be hindered by a cortical cytoskeleton [187–191], and potential metabolic remodeling of the cytoskeleton [192–194]. After initial contact between nanoparticles and membranes, the deformed area of the membrane surrounding the particles increases until half wrapping and then again decreases. While the membrane deformation is catenoid-like for small and for large wrapping fractions of particles, for finite membrane size the highest deformation energy costs are expected for about half-wrapped particles. Formation of a defect in the neck towards the end of the wrapping process can induce the separation of bent and flat membrane and completes wrapping [186].

**Figure 41.**
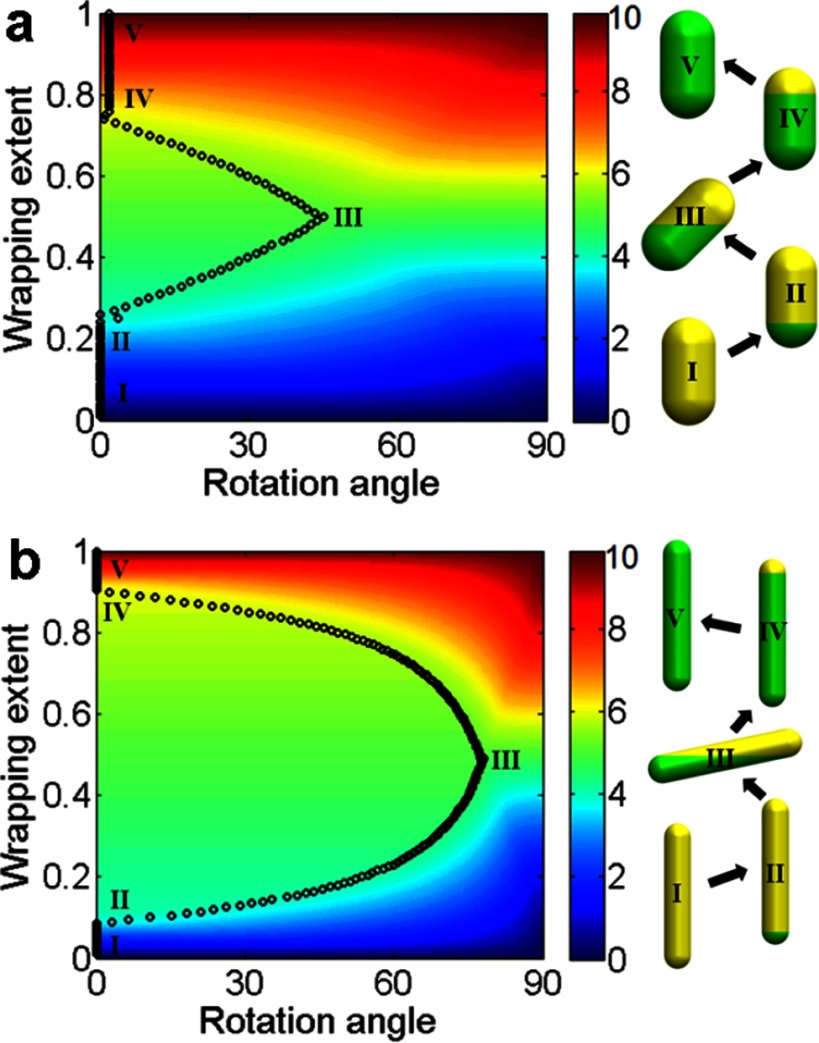

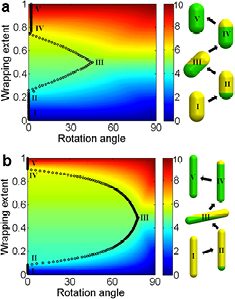

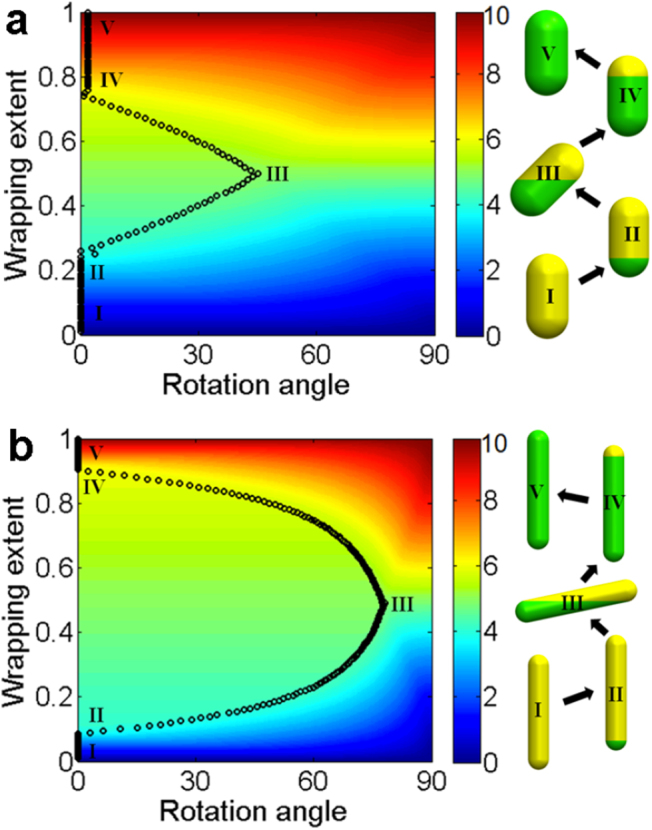
Endocytic pathways for spherocylindrical nanoparticles with aspect ratios (a)  and (b)  predicted by local energetics. The nanoparticles take a general laying-down-then-standing-up sequence during endocytosis. The heat maps show the curvature energy for various particle orientation angles and wrapping fractions. Nanoparticle wrapping at the turning points (I–V) along the endocytic pathways predicted by local free-energy analysis is schematically shown on the right: green-shaded areas are wrapped, while yellow-shaded areas are naked. Reprinted with permission from [186]. Copyright (2013) American Chemical Society.

The reorientation dynamics for elongated particles can be calculated using molecular dynamics simulations for nanoparticles that interact with initially flat lipid bilayer membranes [186, 195]. Also local free-energy analysis and incremental changes of the nanoparticle orientation in the direction of lowest energy at each time step allow to predict the wrapping pathway [186]. In figure 41, the corresponding curvature-energy landscapes are displayed. A spherocylindrical nanoparticle that is initially oriented in the unfavourable rocket orientation first reorients towards submarine orientation. However, although energetically most favourable until half wrapping the particle may never actually reach submarine orientation. Beyond half wrapping, the particle turns back to the then favourable rocket orientation, in agreement with the energetics discussed at the beginning of this section.

#### 3.4.2. Janus particles.

We are not aware of systematic experimental studies for the interaction of lipid-bilayer membranes with nanoparticles that have anisotropic surface functionalization. However, micrometer-sized spherical particles that are half coated with ligands preferably orientate with the ligand-coated side towards the membrane during phagocytosis [196]. Furthermore, viruses have been modeled as partially adhesive particles [140]. The reorientation of the malaria parasite in tip-first orientation at the beginning of the invasion process may also be due to an adhesive gradient on the parasite surface [22]. Analytical calculations for partial-wrapped particles, e.g. Janus particles, at non-spherical vesicles show that membrane curvature-induced forces pull the particles to regions with preferred membrane curvature [197].

#### 3.4.3. Magnetic particle rheology.

In analogy to magnetic particles with switchable orientation at fluid interfaces, micrometer-sized magnetic particles can also be attached to cell membranes with an underlying cytoskeleton [198]. Such microrheological measurements reveal glassy behaviour for various cell types [198–201]. For red blood cells with their cortical spectrin cytoskeleton responsible for the shear elasticity of the complex cell membrane [202], the values of the elastic parameters obtained using magnetic particle microrheology agree well with those used for computer simulations for cell stretching and blood flow [37].

### 3.5. Long-range membrane-mediated interactions

Membrane deformations induced by partial-wrapped particles lead to membrane-mediated interactions that minimise the sum of both membrane deformation energy and particle-membrane attachment energy [203]. Several recent studies are discussed in this section. However, much more is known—and this knowledge may partially be transferred to nanoparticles—about the related systems of lipid bilayer-mediated interactions between curved inclusions, both for membrane-deformation mediated interactions [48, 140, 169, 170, 204], as well as for membrane-fluctuation mediated Casimir interactions [205–206].

#### 3.5.1. Far-field interactions.

Membrane-mediated interaction between two parallel and long cylindrical particles at distance *d* attached to the same side of membranes under lateral tension is repulsive [208],

27 where  is a characteristic reciprocal length, and *a*_cyl_ is the cylinder radius. The interaction between two cylindrical particles attached to opposite sides of the membrane is attractive,

28

Here, the length of the cylinders is contained in the parameters for the bending rigidity, adhesion strength, and membrane tension for these effectively one-dimensional calculations. Results for stronger membrane deformations can be found in [209].

**Figure 42.**
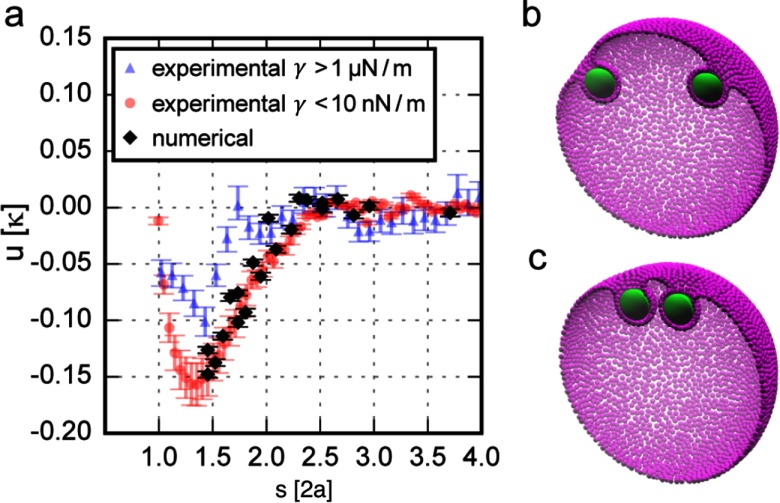

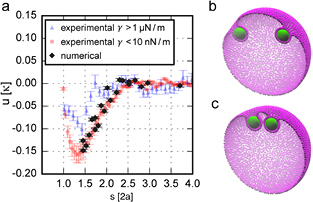

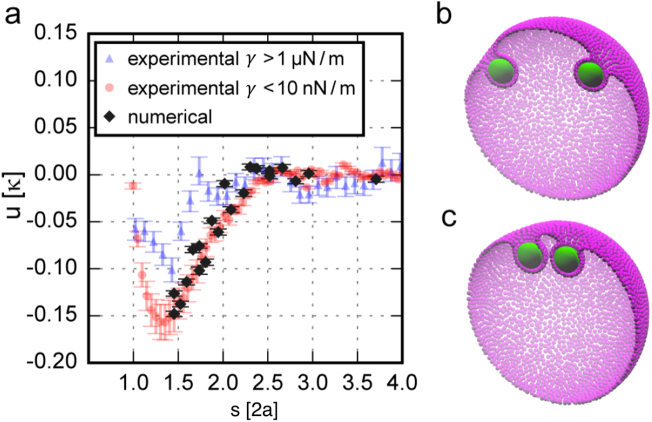
Interaction potentials between two partial-wrapped particles. (a) The numerical results are displayed together with the experimental results, which are rescaled by the bending rigidity  [207] and the particle radius . ((b)–(c)) Simulation snapshots for two particles (green) adhered to a coarse-grained vesicles (magenta) at separations (b)  and (c) . The membrane vertices are plotted using small spheres. Adapted with permission from [44]. CC BY 4.0.

Only few studies are available for far-field interactions between spherical nanoparticles that are partially attached to lipid bilayer membranes. Figure 42 shows both numerical calculations and experimental data. Membrane-mediated particle attraction is found for distances below , with an attractive energy well of  for an interparticle distance of about  [44]. For typical lipid bilayer bending rigidities , this corresponds to binding energies . The figure shows larger attraction strengths for membrane tensions  compared with membrane tensions .

Many computer simulation studies for membrane-mediated interactions between partial-wrapped nanoparticles, as shown in figure 42, rely on finite-element calculations. Here, the bending energy is discretized on triangulated surfaces [210–213]. Large systems that require at the same time fine discretization in high-curvature regions around particles are computationally expensive, therefore membrane-mediated interactions between the particles have so far mostly been studied in the near field. However, long-ranged membrane-mediated interactions between partial-wrapped nanoparticles might be similar to long-ranged interactions between curved inclusions. For example, for two spherical-cap inclusions attached to the same side of planar membranes, the repulsive membrane-mediated interaction is [169]

29 where  and  are polar angles that determine the sizes of the spherical caps, *a* is their radius, and *d* is the distance between the centers of the caps. Strongly-curved inclusions at very close distances experience an attractive interaction [48].

#### 3.5.2. Near-field interactions.

Interactions between two (spherical) nanoparticles have been shown to be attractive, see e.g. figure 42. This is surprising on the first view, because catenoid-like membrane deformations around partial-wrapped nanoparticles at large distances require only very small deformation-energy costs. If two spherical-cap inclusions or two particles approach each other on a planar membrane the ideal catenoid-like deformations cannot form any more because of the boundary conditions. Therefore, the membrane-mediated interactions because of deformation-energy are expected to be repulsive [169, 204]. However, for spherical particles the attractive membrane-mediated interactions because of membrane-particle adhesion energy dominate and lead to an overall attraction [203].

For two partial-wrapped particles in the near field, not only the interparticle distance, but also the orientation of the pair of particles with respect to the membrane has to be taken into account. For example, in [46] the connecting line between two particles adsorbed to a vesicle is parallel to the membrane at large distances and reorients to perpendicular orientation at small distances. The attractive interaction is significantly higher for this particularly stable tubular arrangement [214].

### 3.6. Many-particle interactions

**Figure 43.**
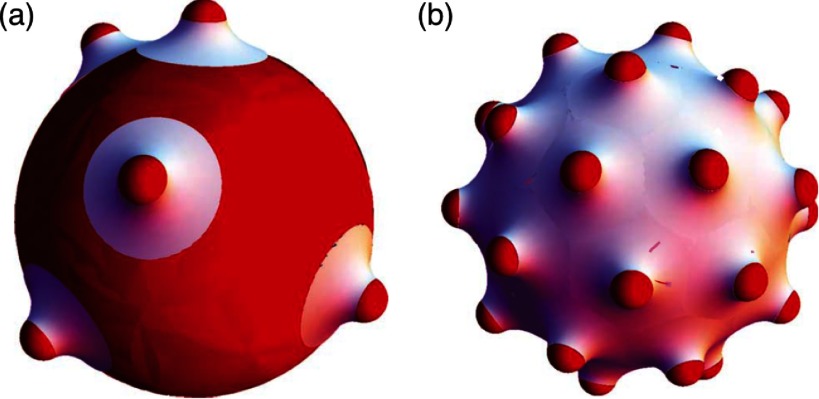

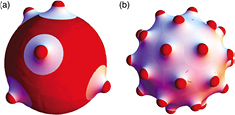

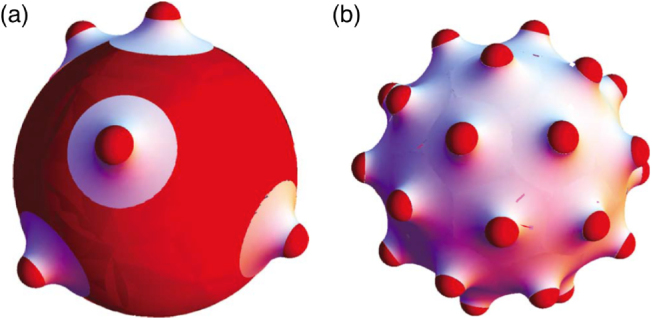
Spherical-cap inclusions on vesicles. (a) Inclusions, indicated by small red caps, on a vesicle at low density. White membrane patches indicate catenoidal deformations with vanishing bending energy around the inclusions. (b) An inclusion-decorated vesicle with vanishing total bending energy at optimal inclusion density. Reprinted with permission from [170]. Copyright (2009) by the American Physical Society.

Many-particle interactions are important for membrane-mediated interactions, which has been demonstrated in several cases for integral membrane proteins with hydrophobic mismatch and for spherical-cap inclusions [140, 170, 182, 215]. It is therefore essential to not only consider pair interactions between particles. We start our discussion with the ‘inclusion case’, i.e. with membrane deformation-mediated interactions only, where the membrane area attached to each particle is fixed. While two weakly-curved spherical-cap inclusions that are attached to the same side of the membrane repell each other as discussed in section 3.5.1, many inclusions have been observed to aggregate and to induce bud formation [140, 170, 215–217]. A membrane that is curved prior to adhesion of inclusions or that gets curved by cooperative budding screens the repulsive interaction and can therefore lead to effective attraction. Figure 43 highlights catenoidal membrane deformations around inclusions on vesicles; the size of the catenoidal ‘halo’ shrinks with increasing background curvature, thereby screening the repulsive interaction between the inclusions. These catenoidal patches reduce the total bending energies of the vesicles, which vanish at optimal inclusion density. In comparison, on planar membranes curved inclusions always increase the deformation energy.

A particle dimer switches from a linear aggregate on the membrane to a tubular aggregate for increasing wrapping fraction [46, 47], compare also section 3.4 for the orientation of elongated particles. On a vesicle, the wrapping fraction can be tuned by adjusting the reduced volume of the vesicle, , where *V* is its actual volume and *V*_sph_ is the volume of a spherical vesicle with the same membrane area. A small reduced volume also allows more than two particles to join the tube. The energy gain of such tubular assemblies compared with single, complete-wrapped particles strongly depends on the ratio of the range *ρ* of the particle-membrane interaction potential and the particle radius *a*. The finite potential range leads to a higher adhesion energy gain for tubular arrangements compared with single-particle buds, because some fraction of the tubular necks between particles are inside the interaction range; for example the energy gain per particle is about  for  [214].

**Figure 44.**
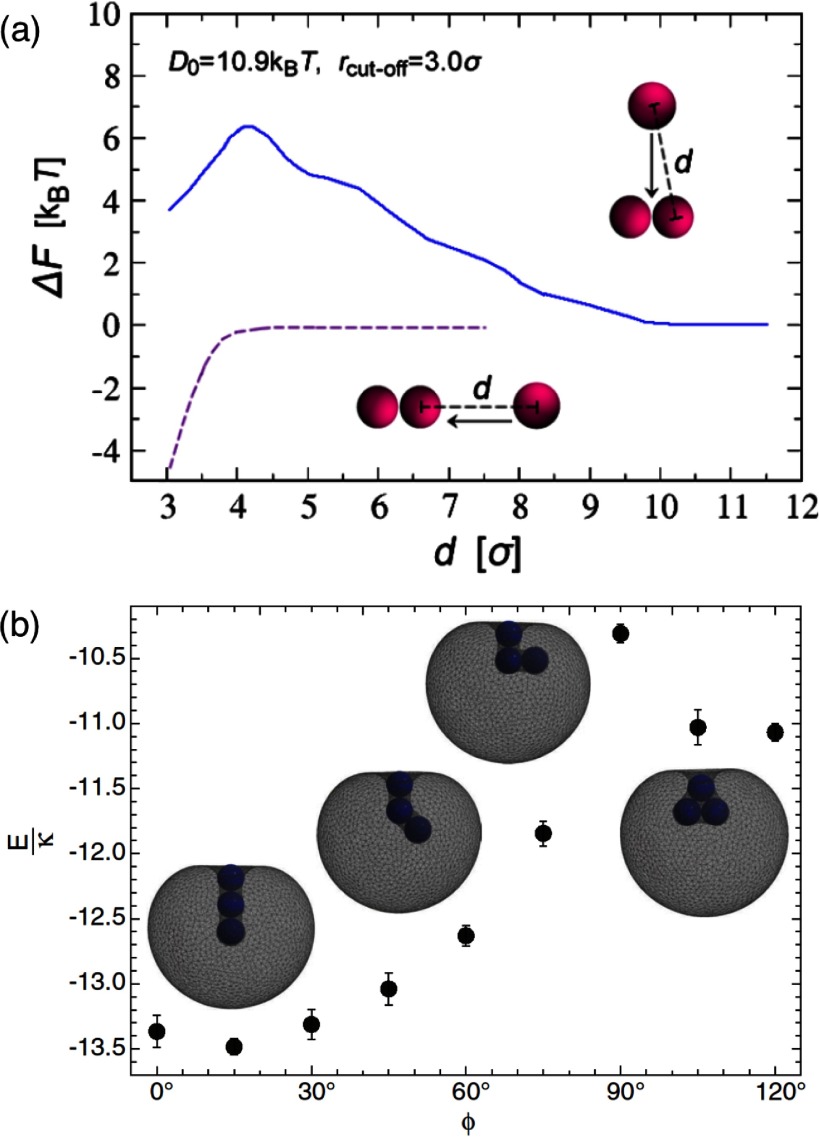

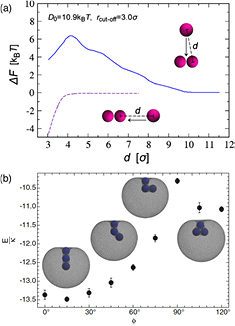

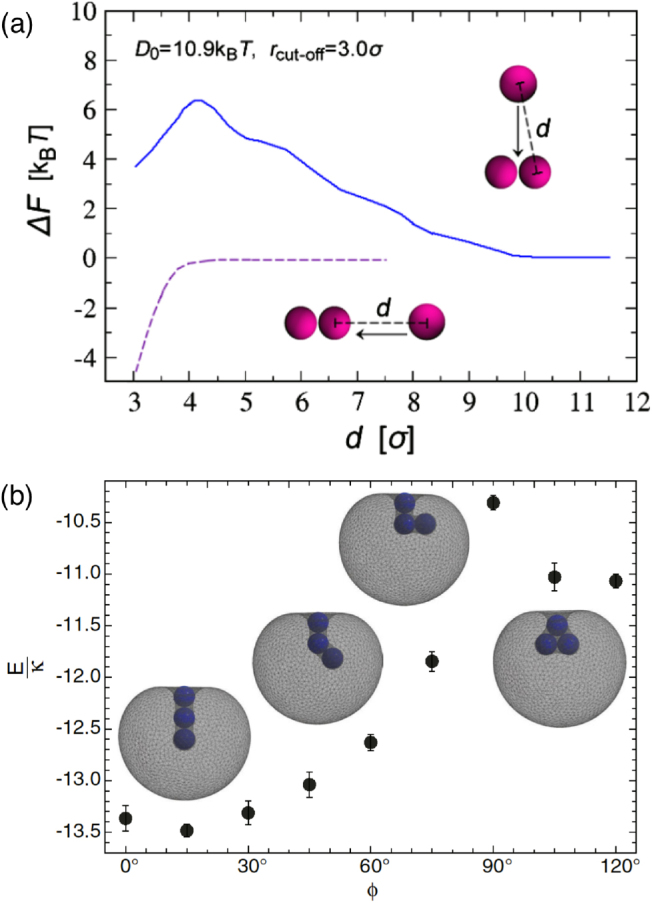
Membrane-mediated interactions between three spherical particles. (a) Total energy as a function of the separation when a third particle approaches a fixed dimer along the direction of the dimer’s axis (dashed line) and perpendicular to it (full line). In both cases  and  has been used, where *σ* is the diameter of spherical hard beads used to model the membrane. Reprinted with permission from [203]. Copyright (2012) by the American Physical Society. (b) Rescaled total energy  of a vesicle with three adsorbed particles bound together by membrane tubes as a function of the angle *ϕ* between the particles for the reduced volume  and the rescaled adhesion energy . The membrane area of the vesicle is . Here,  corresponds to a linear arrangement of the particles. The four snapshots depict minimum-energy conformations at the angles , , , and . Reprinted with permission from [46]. Copyright (2012) by the American Physical Society.

Simulation snapshots and energies for several configurations of three particles attached to membranes are shown in figure 44. If the particles are all located in the plane of the membrane, a third particle that attaches to form a linear aggregate gains membrane-mediated binding energy of few  and attaches without an energy barrier. In contrast, a particle that attaches from the side to the existing particle aggregate experiences an energy barrier and has a few  higher energy in the (metastable) bound state compared with the unbound state. Bending energy has been shown to favour compact aggregation, adhesion energy linear aggregation [203]. Also for tubular aggregates, the linear-tube configuration is preferred over the more compact, triangular configuration [46], see figure 44(b). Linear aggregates in the plane of the membrane have been experimentally observed for colloidal particles bound to giant unilamellar vesicles (GUVs) [218], tubular aggregates for the interaction of viruses with cells and GUVs [219].

**Figure 45.**
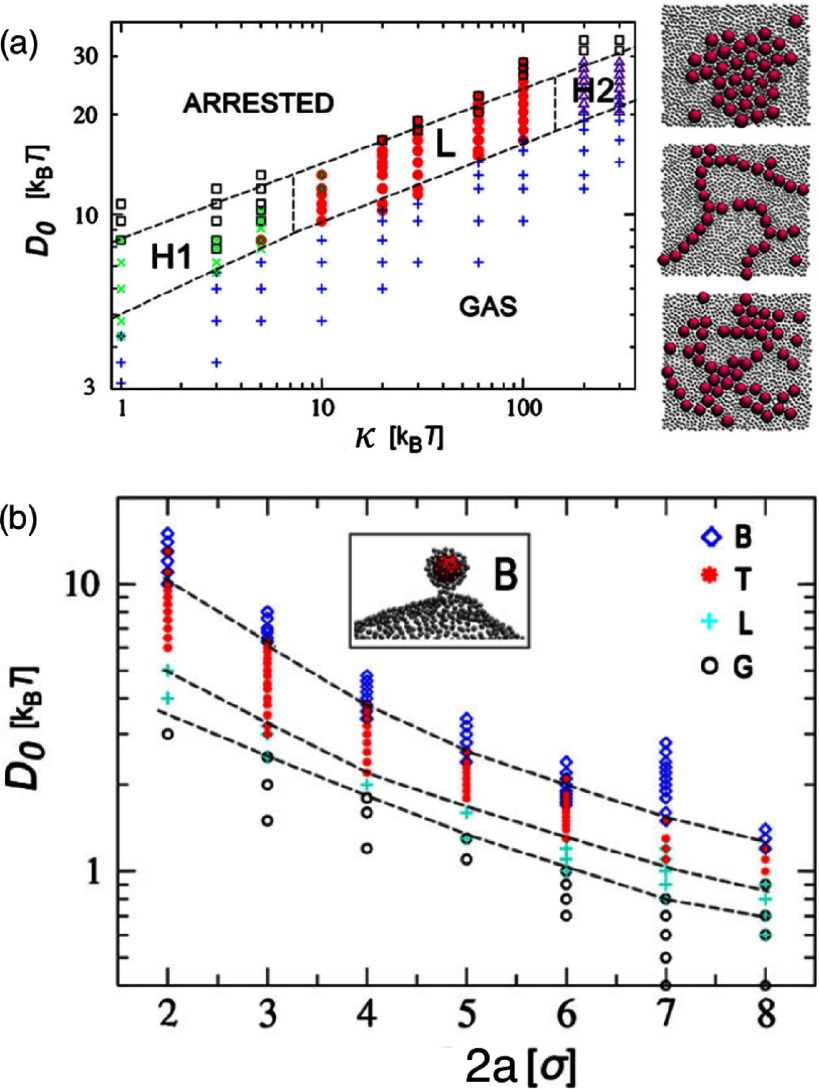

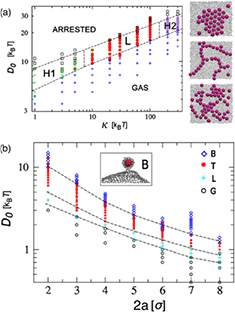

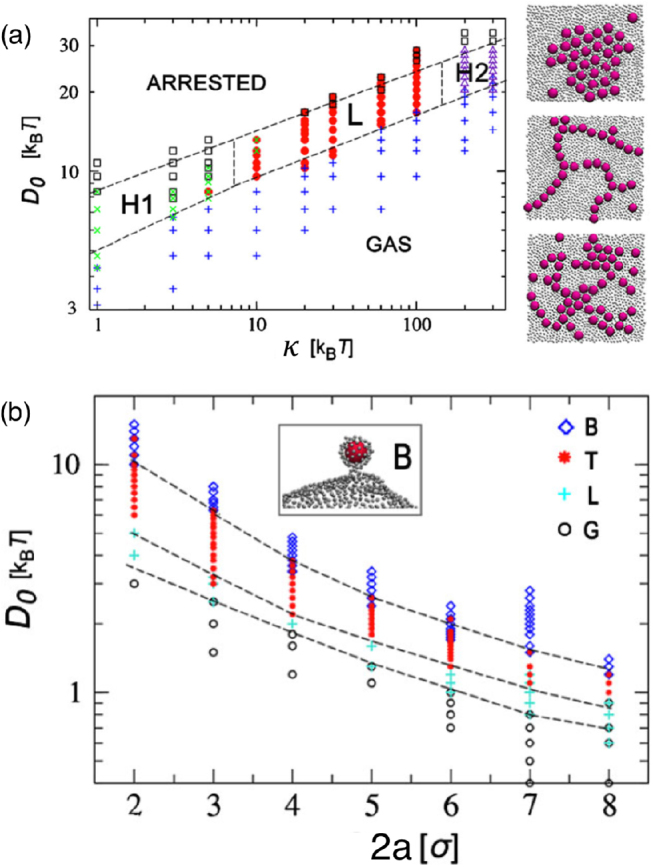
Membrane-mediated interactions between many particles. (a) Phase diagram for nanoparticle self-assembly in terms of membrane bending rigidity *κ* and particle-membrane binding energy *D*_0_, which replaces the adhesion strength *w* in the simulations. The snapshots show typical aggregates in the H1, the L, and the H2 phase (top to bottom). Adapted with permission from [203]. Copyright (2012) by the American Physical Society. (b) Phase diagram in terms of nanoparticle diameter  and *D*_0_. With increasing *D*_0_, the gaseous phase G with non-attached particles is followed by the linear aggregation phase L, the tube-formation phase T, and the single-particle bud phase B. The radius of the vesicle is  and the particle surface fraction is 0.15. Adapted with permission from [47]. Copyright (2012) by the American Physical Society.

Figure 45(a) shows a phase diagram for many-particle systems in terms of bending rigidity *κ* and particle-membrane binding energy *D*_0_ [203]. In the limit of small bending rigidities, partial-wrapped particles form a hexagonal cluster phase where the membrane penetrates in-between the particles. In the limit of high bending rigidities, the particles are barely attached to the membrane. They deform the membrane only weakly, therefore they also interact only weakly and are found in loose, mostly hexagonal aggregates. Linear aggregates are observed inbetween both limites for biologically relevant bending rigidities . Figure 45(b) shows a phase diagram in terms of particle diameter and particle-membrane binding energy. Free unbound nanoparticles are found at low adhesion strengths and for small particle radii, then linear aggregates, tubular aggregates, and single-particle buds are observed with increasing adhesion strength [47]. This sequence of configurations is consistent with the reorientation reported for elongated nanoparticles in [176], see section 3.4.

### 3.7. Membranes with spontaneous curvature

Biological membranes are often not symmetric, the two monolayers usually consist of different lipids. This asymmetry can be modeled with the help of a spontaneous curvature *c*_0_ of the membrane. If the directions of spontaneous curvature and the curvature of the particle surface coincide, the spontaneous curvature facilitates complete wrapping compared with a symmetric membrane [42]. At the same time, the direct transition between free and complete-wrapped states is replaced by a discontinuous transition. For a spontaneous curvature opposite to the curvature of the particle surface, the complete-wrapped state is shifted to higher adhesion strengths. Furthermore, a new regime with partial-wrapped states is found. With increasing spontaneous curvature, also the regime of adhesion strengths where partial-wrapped states are stable increases.

**Figure 46.**
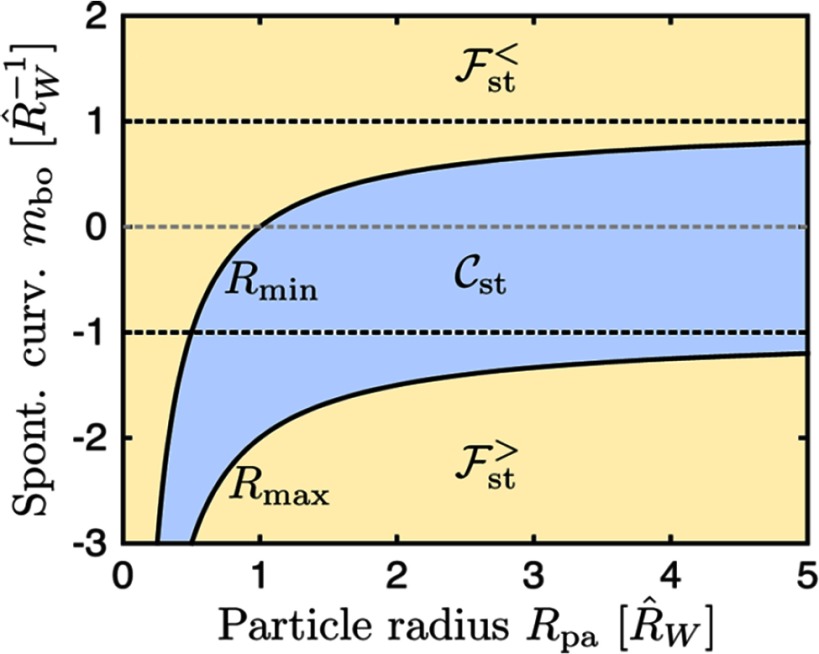

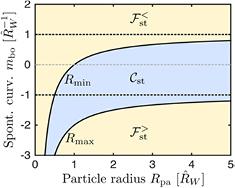

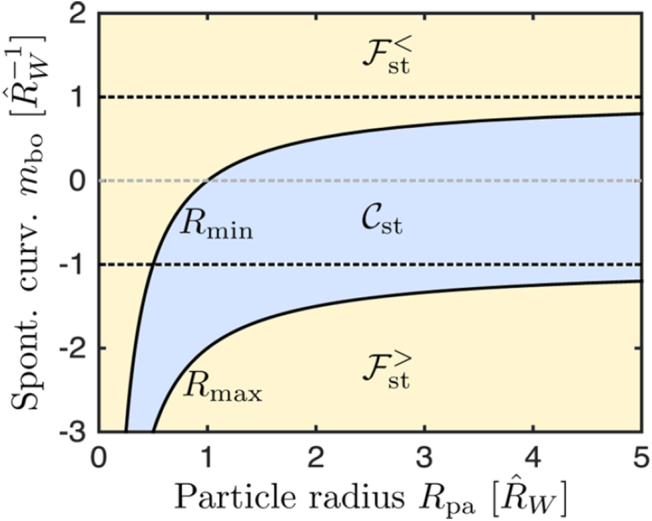
Engulfment regimes for weakly curved mother membranes as a function of particle radius  and spontaneous curvature  of the membrane segment bound to the nanoparticle. Both quantities are given in units of the modified adhesion length . The direction of negative spontaneous curvature coincides with direction of the curvature of the particle surface. Complete-wrapped states  (blue) are found between the two regimes with non-wrapped states,  and  (yellow). The phase boundaries are given by  for  and  for . Reprinted with permission from [42]. Copyright (2015) American Chemical Society.

For fixed adhesion strengths, finite spontaneous curvatures lead to size selectivity for wrapping of nanoparticles, see figure 46. For vanishing or small spontaneous curvatures, all particles with radii beyond a threshold radius get wrapped, see equation (26). With decreasing spontaneous curvatures, in addition to the lower threshold radius for complete wrapping, also an upper threshold radius beyond the that particles remain unwrapped is found [42]. The regime with stable complete-wrapped states thus narrows to a small window in the particle radius for high negative values of the spontaneous curvature. Such a preferred radius for particle wrapping has been observed in experiments [220, 221]. Size selectivity by spontaneous membrane curvature is therefore an alternative to receptor-based models for cellular uptake that are usually used to motivate an upper limit for the particle radius to achieve complete wrapping [154].

### 3.8. Applications

On the one hand, particle-membrane systems can be used for applications in therapeutics and diagnostics. On the other hand, for numerous other applications in industry, potential toxic effects have to be considered. Furthermore, nanostructured surfaces can be rationalised as nanoparticles bound to a substrate, which opens an entire new field for applications. For basic research, particle-membrane systems can serve as model systems to understand biological processes, such as viral budding, malaria invasion, and phagocytosis.

#### 3.8.1. Drug delivery.

Nanoparticles are potential drug-delivery vectors and tools for diagnosis [222–226]. For diagnosis, in particular non-spherical nanoparticles with enhanced stability of partial-wrapped states can serve as membrane markers for imaging [141]. For drug delivery, rough hydrophobic particles have been proposed to have a high drug-carrying capacity and high loading efficiency [227]. Such particles can prolong the time of release of a drug payload, which can be therapeutically advantageous. Nanoparticles may even cross the blood-brain barrier via transcytosis and may therefore be applied for drug delivery in the brain [228, 229]. Recently hollow shell-shell nanocontainers with stimuli-responsive properties that could allow for controlled drug release have been suggested for drug delivery [230].

#### 3.8.2. Nano-Toxicity.

**Figure 47.**
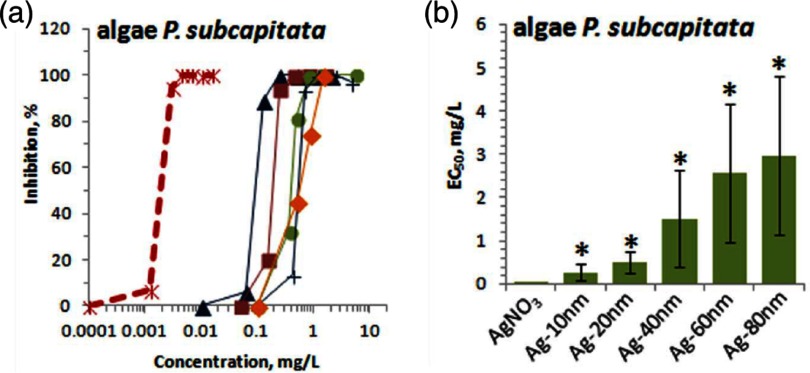

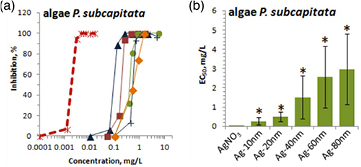

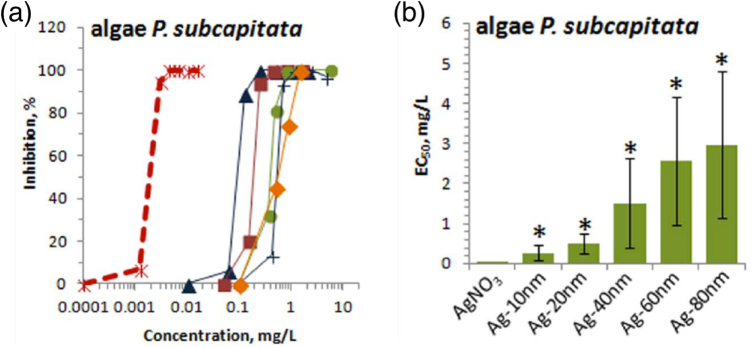
Dose-response curves and respective  values of Ag formulations to test algae. (a) Dose-response curves ((red) , (blue,triangle) Ag nanoparticles with size , (brown) , (green) , (blue, +) , (orange) ) and (b)  values for algae P. subcapitata, a very sensitive test organism. Reprinted with permission from [19]. CC BY 4.0.

For studies of potential toxic effects, not only single-particle properties, such as composition and size, but also particle concentrations play an important role [19]. Toxicity can be quantified by  values that refer to the concentration of particles that induce a response of cells or organisms halfway between the base line and the maximum after  exposure time to nanoparticles. While  leads to growth inhibition for algae already at concentrations of ,  nanoparticles lead to growth inhibition only at concentrations between  and , see figure 47. Most other organisms are less sensitive to nanoparticles and growth inhibition occurs only at higher particle concentrations compared with algae. In general, the  values increase significantly with particle size, which means that the toxic effect is reduced. For mammalian cells they range from  for  Ag particles to  for  Ag particles, for *E. coli* bacteria from  for  Ag particles to  for  Ag particles.

#### 3.8.3. Nanostructured surfaces.

**Figure 48.**
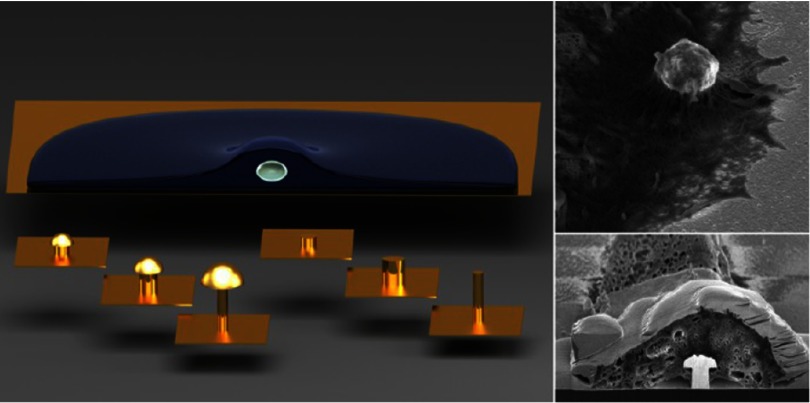

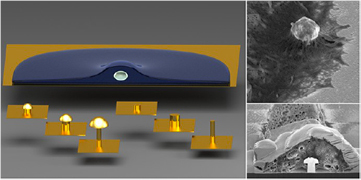

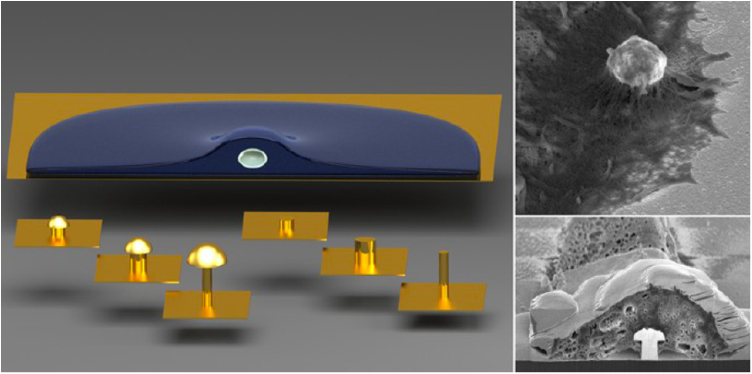
A cardiomyocyte cell interfacing with cylindrical and mushroom-shaped gold nanopillars. Focused ion-beam cuts of cells that engulf mushroom-shaped pillars show the shape of the lipid-bilayer membranes. Reprinted with permission from [231]. Copyright (2014) American Chemical Society.

Multi-electrode arrays allow to electrically couple excitable cells to electronic devices, see figure 48. The challenge is to achieve an optimal coupling, which requires to minimize the cleft between the cell and the electrode and to achieve tight attachment of the cell to the substrate in general. While thin electrodes pierce cell membranes [232], micro-electrodes get wrapped [231, 233]. A systematic study of different micro-electrode shapes reveals that mushroom-shaped pillars are engulfed more than cylindrical pillars without caps [231]. Furthermore, cells have been found to preferably engulf pillars in their center compared with their edge. Quantitative evaluation of focused ion-beam cuts allows to extract normal cytoskeletal stresses between few  for cylinders with caps and several hundred  for cylinders without caps.

#### 3.8.4. Viral budding, malaria invasion, and phagocytosis.

**Figure 49.**
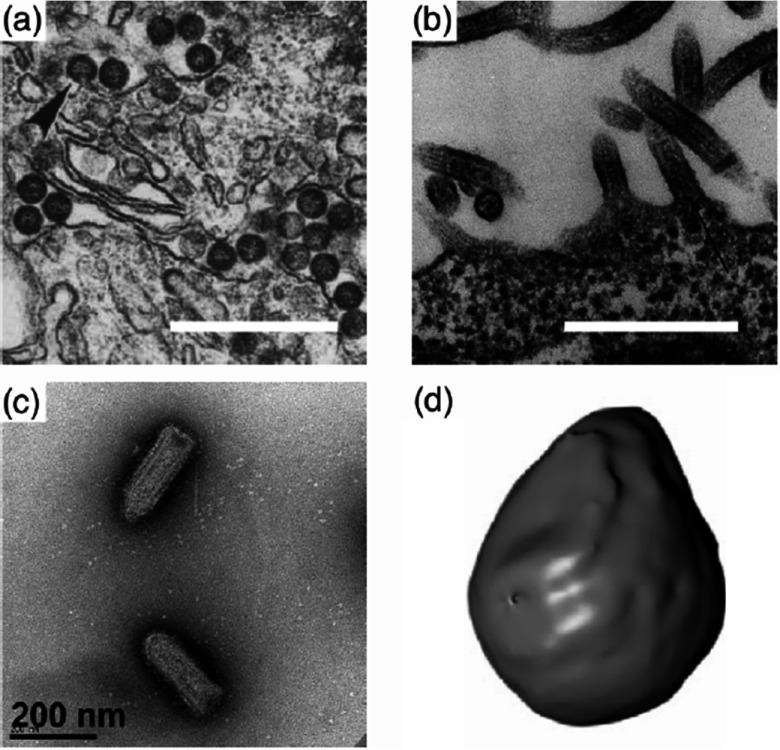

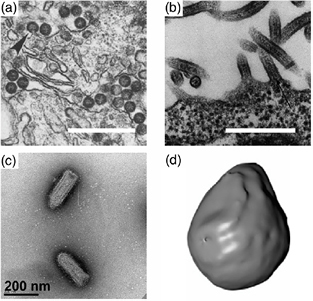

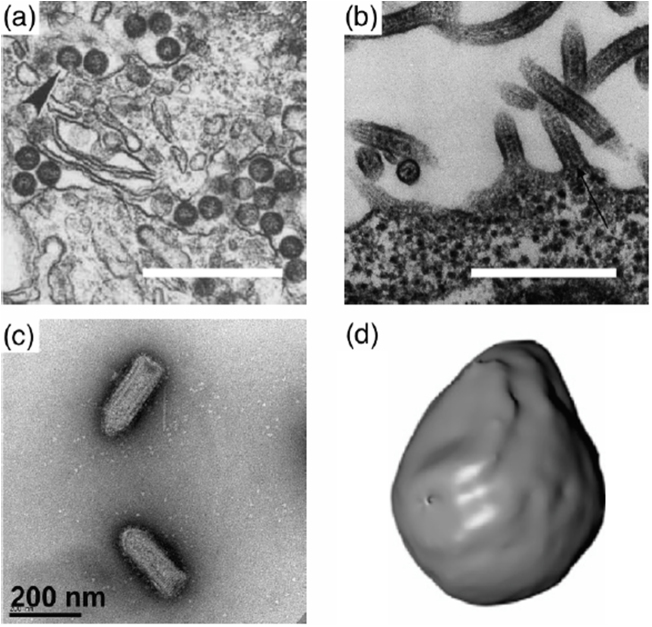
Viruses and parasites. (a) Rift valley feaver viruses in primary rat hepatocytes. The length of the scale bar corresponds to 600 nm. Adapted from [234], with permission from Elsevier, Copyright (1987). (b) Virus-like particles as seen in nucleocapsids of Ebola virions, about to bud. The length of the scale bar corresponds to 500 nm. Reprinted with permission from [28]. CC BY 4.0. (c) Vesicular stomatitis virus (VSV). The length of the scale bar corresponds to 200 nm. Reprinted with permission from [235]. CC BY 4.0. (d) An isosurface-rendered malaria parasite. Reprinted with permission from [22]. CC BY 4.0.

**Figure 50.**
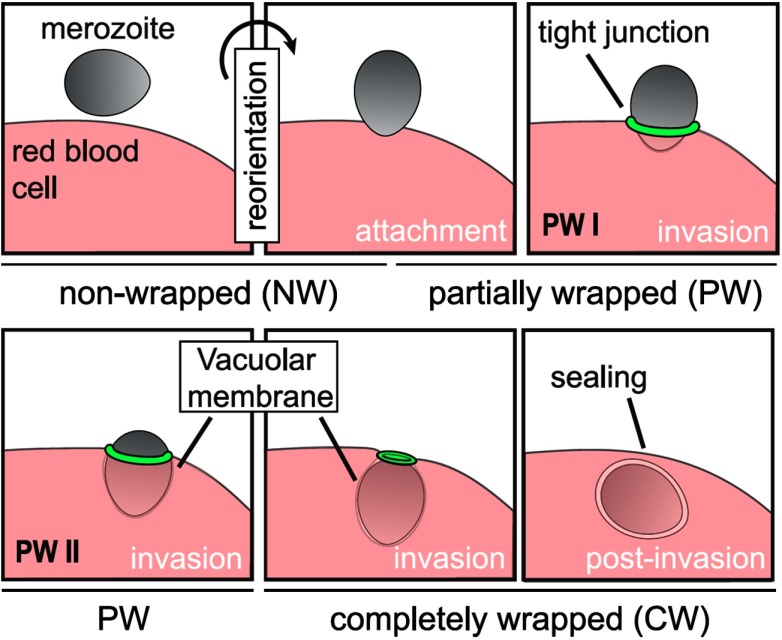

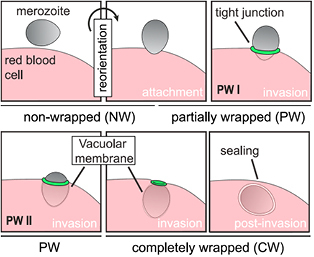

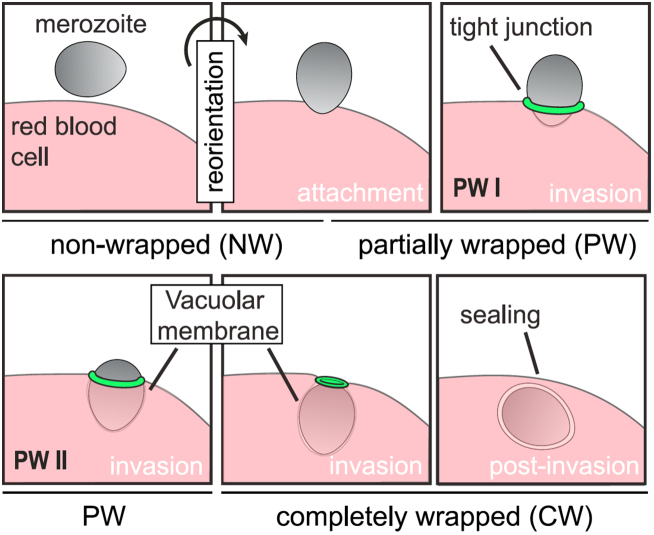
The stages of merozoite invasion. Schematic representation depicting different wrapping phases of the merozoite from reorientation through to invasion and postinvasion. Reprinted with permission from [22]. CC BY 4.0.

Passive endocytosis of particles with a homogeneous adhesion strength between particles and membranes is often modified by specific adhesion and active processes for biological systems. Figure 49 shows viruses with different shapes and a malaria parasite that have similar sizes as the particles discussed earlier. One specific example for active wrapping is the invasion of the malaria parasite into an erythrocyte, see figure 50. Reorientation of the parasite, possibly because of a gradient of adhesive molecules, is followed by invasion. During invasion, a tight junction forms and reseales the membrane, which completes the formation of the parasitophorous vacuole.

**Figure 51.**
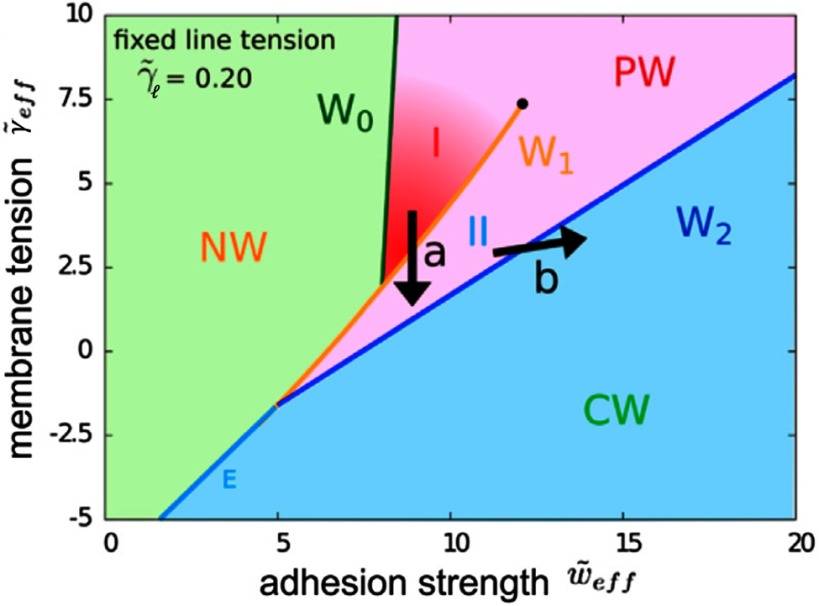

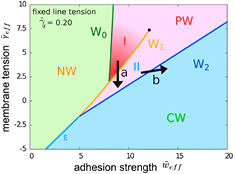

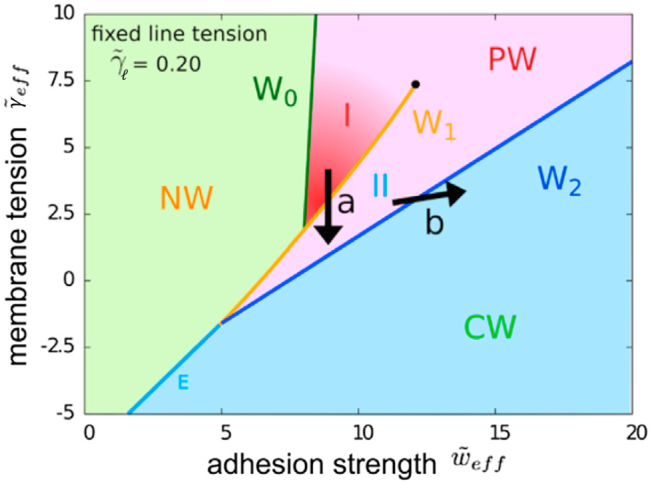
Wrapping phase diagram for a tip first-oriented merozoite for fixed reduced line tension  and several values of effective adhesion strength and effective membrane tension: non-wrapped merozoites (NW), partial-wrapped merozoites with small (PW I) and high wrapping fractions (PW II), and complete-wrapped/fully invaded merozoites (CW), see figure 50. The transition *W*_0_ is a continuous transition, whereas the transitions *W*_1_, *W*_2_, and *E* are associated with energy barriers. The transition *W*_1_ ends at a critical point where the difference between PW I and PW II vanishes. The spontaneous curvature *c*_0_ can be combined with the membrane tension and the adhesion strength to an effective membrane tension, , and an effective adhesion strength, , respectively. The critical point is indicated by a black point. Here, , , , where  and *a* is the radius of a sphere with the same surface area as the meozoite. Modified with permission from [22]. CC BY 4.0.

For the egg-like shape of the malaria parasite, line tension at the tight junction, and anisotropic adhesion of the parasite leads to a complex phase diagram for passive endocytosis with stable non-wrapped, shallow-wrapped, deep-wrapped, and complete-wrapped states [22], see figure 51. As indicated in figure 51, secretion of unstructured membrane from the parasite and favourable spontaneous curvature of the erythrocyte membrane may help the parasite to overcome energy barriers for wrapping and to finally invade the cell. In addition, also motor forces have been proposed to assist invasion [22].

**Figure 52.**
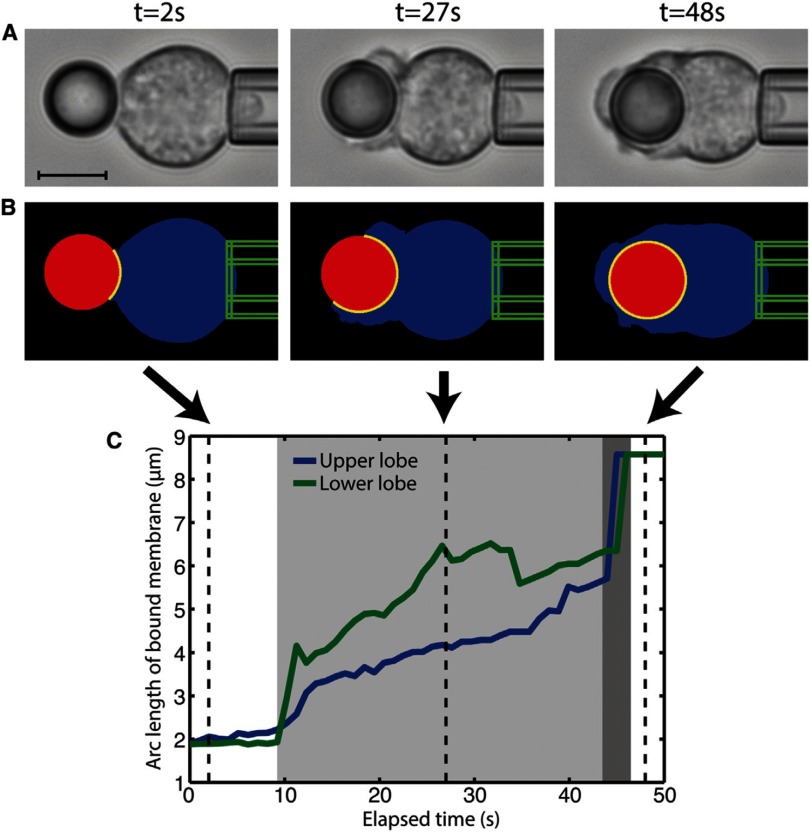

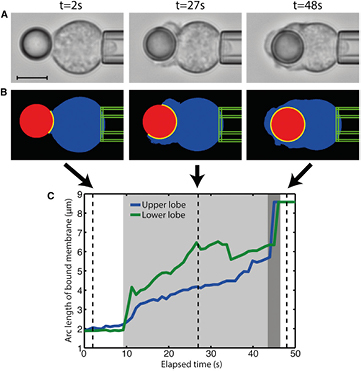

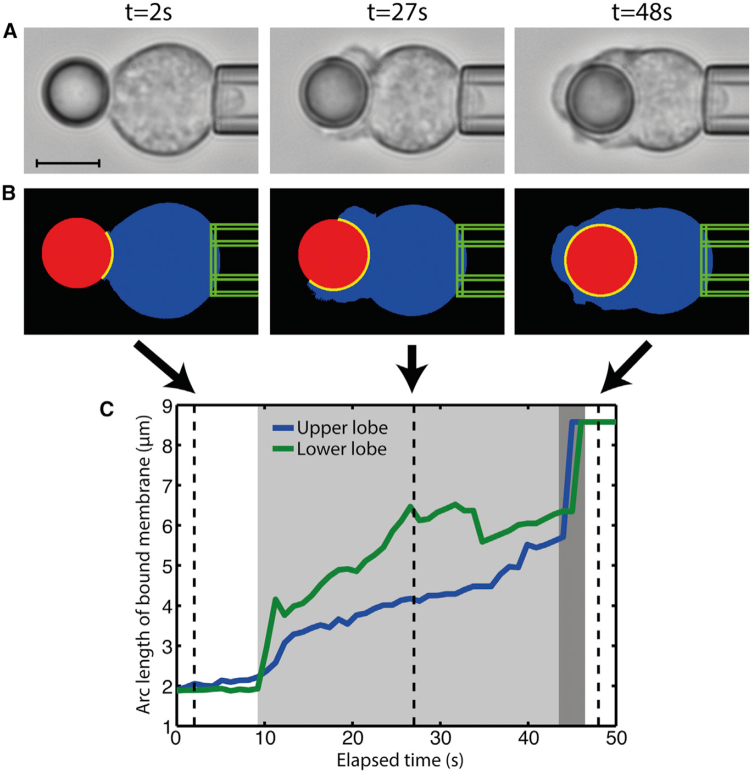
Typical time-lapse movie and image analysis of a neutrophil engulfing an IgG-coated bead with radius . The data has been taken from [236]. (A) Raw images of three frames at various stages of engulfment. At , the bead has been released onto the cell, with a contact area of . At this point engulfment has not yet started. At  the bead is approximately half-engulfed, with the lower lobe noticeably ahead of the upper lobe. At  engulfment is complete, the bead is entirely within the cell, and the phagosome is fully formed. The length of the scale bar corresponds to . (B) The same frames as in panel A after automatic image analysis: (blue) cell, (red) bead, (green) outline of the pipette, (yellow) membrane attached to the bead. (C) Engulfment as function of time. For both the upper and lower lobes, after engulfment begins at  there is an initial slow stage (light gray) followed by a much quicker second stage (dark gray). Engulfment is complete by . Reprinted from [237], Copyright (2014), with permission from Elsevier.

Another active biological ‘wrapping’ process is phagocytotic uptake, see figure 52. Here, the growth of the phagocytotic cup with its actin cytoskeleton is the major dynamic process [238]. Quantification using microscopy and image analysis shows fast uptake at the beginning, followed by a plateau with weakly increasing wrapping fraction, and again fast uptake of the particle towards the end of the process [237]. Various mechanisms for phagocytotic uptake have been suggested, among them receptor diffusion and directed motion [239], a zipper-like mechanism based on membrane fluctuations and membrane adhesion to the particle [240], and hindered uptake by increase and relaxation of membrane tension [241].

## 4. Conclusions

Multi-phase fluid systems and cellular biological systems abound with interfaces. Nano- and microparticles naturally collect at such interfaces, because their localization at the interfaces lowers their interaction energy with the environment. We have considered here two types of interfaces: fluid-fluid interfaces governed by interfacial tensions, and biological interfaces controlled by curvature elasticity. Particles at both types of interfaces show many common behaviours. The interface attraction depends on particle size, shape, and surface properties. Particles can orient in different ways at interfaces, they deform the interfaces around them, and these interface deformations lead to interface-mediated interactions and collective behaviour.

Although systems and concepts for particles at fluid and biological interfaces are very similar, and for instance SDS stabilisation of droplets can drive the system from fluid interfaces towards membranes [242], there is a major difference. In many cases, particle sizes at fluid interfaces are in the micrometer range, while particle sizes at lipid bilayer membranes are in the nanometer range. This implies that different experimental techniques are required in both cases, where the nanometer scale makes particles at membranes more difficult to observe and more difficult to manipulate. Therefore, we have a better knowledge about particles at fluid interfaces, and there is an urgent need for systematic and well-controlled experiments for particles at membranes. Nevertheless, a unified numerical and theoretical description of both systems is possible—at least for particles above a threshold diameter of about —on the basis of continuum models for two-dimensional surfaces and their deformations embedded in three-dimensional space.

From the technological point of view, micrometer-sized particles at fluid interfaces can be used in many ways to control and tailor interface properties. Such applications range from emulsion stabilisation through an effective reduction of the interface tension to the design of solid shells and surfaces with controlled optical properties. From a biological point of view, nanoparticles are interesting in nanomedicine as biomarkes and drug carriers, but their effect on cells also has to be assessed because of their potential nanotoxicity. In addition, there is a large range of biological particles, such as viruses and parasites, the interaction of which with cells is also highly desirable to be controlled.

The interaction of single hard particles with interfaces is by now reasonably well understood. Therefore, we believe that future research should move towards particle-mediated interactions and collective properties for many particles at both fluid and biological interfaces, as well as towards soft particles. For example, size and concentration effects for the interaction or nanoparticles with liposomes and polymersomes have been studied using electron microscopy and scattering [49–51]. Using force measurements, nanoparticle-mediated adhesion between elastic gels has been measured [243]. The hard particles can serve as ‘glue’ between soft interfaces. Soft particles, e.g. microgel and polymeric particles, add further complexity by allowing the particle shape to adjust in response to the interaction with a fluid or biological interface [160, 244–248].

Finally, dynamical behaviour of particles at fluid and biological interfaces awaits further characterization. For instance, dynamics of wrapping of non-spherical particles by biological interfaces has been discussed in section 3.4.1, but calculations that include Brownian motion are missing. Colloids adsorbed to stabilised oil–water interfaces show different diffusion properties than colloids in bulk [242], systematic studies could be used to measure the viscosity of lipid bilayers. Furthermore, nanoparticles in combination with superresolution microscopy can be used as probes to study dynamics in biological cells [249–251].

To conclude, particles are already widely used for applications, but a systematic understanding of the interactions of engineered nano- and microparticles with soft and biological matter is often lacking. Furthermore, our understanding of basic biological processes on the cellular scale, such as phagocytosis and blood-stage malaria, will benefit from a detailed understanding of particles at biological interfaces. We expect that in the future more systematic studies for particles at fluid and biological interfaces will allow the engineering of even better tailored nano- and microparticles for applications and to achieve a more thorough understanding of cellular uptake and invasion mechanisms. Examples for areas where rational design of interface-particle systems may improve applications include targeted drug delivery for particles at biological interfaces, and a better control of the rheological properties of emulsions for particles at fluid interfaces.

## Supplementary Material


